# Complete Mitochondrial Genomes of *Metcalfa pruinosa* and *Salurnis marginella* (Hemiptera: Flatidae): Genomic Comparison and Phylogenetic Inference in Fulgoroidea

**DOI:** 10.3390/cimb43030099

**Published:** 2021-09-30

**Authors:** Min Jee Kim, Keon Hee Lee, Jeong Sun Park, Jun Seong Jeong, Na Ra Jeong, Wonhoon Lee, Iksoo Kim

**Affiliations:** 1 Department of Applied Biology, College of Agriculture and Life Sciences, Chonnam National University, Gwangju 61186, Korea; minjeekim3@korea.kr (M.J.K.); dlrjsgml0803@naver.com (K.H.L.); jungsun5009@naver.com (J.S.P.); jjszzang1234@naver.com (J.S.J.); ioveskfk@naver.com (N.R.J.); 2 Experiment and Analysis Division, Honam Regional Office, Animal and Plant Quarantine Agency, Gunsan 54096, Korea; 3 Team of Protected Area Research, National Institute of Ecology, Seocheon 33657, Korea; 4 Department of Plant Medicine and Institute of Agriculture & Life Sciences, Gyeongsang National University, Jinju 52828, Korea; wonhoon@gnu.ac.kr

**Keywords:** flatid planthopper, *Metcalfa pruinosa*, *Salurnis marginella*, complete mitochondrial genome, phylogeny

## Abstract

The complete mitochondrial genomes (mitogenomes) of two DNA barcode-defined haplotypes of *Metcalfa pruinosa* and one of *Salurnis marginella* (Hemiptera: Flatidae) were sequenced and compared to those of other Fulgoroidea species. Furthermore, the mitogenome sequences were used to reconstruct phylogenetic relationships among fulgoroid families. The three mitogenomes, including that of the available species of Flatidae, commonly possessed distinctive structures in the 1702–1836 bp A+T-rich region, such as two repeat regions at each end and a large centered nonrepeat region. All members of the superfamily Fulgoroidea, including the Flatidae, consistently possessed a motiflike sequence (TAGTA) at the *ND1* and *trnS_2_* junction. The phylogenetic analyses consistently recovered the familial relationships of (((((Ricaniidae + Issidae) + Flatidae) + Fulgoridae) + Achilidae) + Derbidae) in the amino acid-based analysis, with the placement of Cixiidae and Delphacidae as the earliest-derived lineages of fulgoroid families, whereas the monophyly of Delphacidae was not congruent between tree-constructing algorithms.

## 1. Introduction

In Hemiptera, ~1670 mitochondrial genomes (mitogenomes) are available, and for the suborder Auchenorrhyncha, which is composed of two infraorders, Cicadomorpha and Fulgoromorpha, 982 mitogenomes are available (as of August 2021). In Auchenorrhyncha, the sequences of 558 mitogenomes are available for the infraorder Fulgoromorpha, which consists of the monotypic superfamily Fulgoroidea and comprises 13,428 species of 2350 genera in 21 families, but approximately half of these are from the same species (e.g., *Lycorma delicatula* in Fulgoridae) [1,2]. Therefore, complete or near complete mitogenome sequences are available for a total of 20 species (excluding nine unknown species) of 22 genera from 10 subfamilies and eight families. Therefore, mitogenome sequences are available for a limited number of Fulgoroidea, presenting only one genus in many subfamilies. These circumstances hamper phylogenetic scrutiny, reconstruction and evolutionary genomic comparison at various levels of taxonomic groups and, thus, require additional mitogenome sequences from a diverse taxonomic group.

The mitogenome sequences in Fulgoroidea have been used in several studies of evolution, including those involving mitogenomic announcement, illustration of intraspecies diversity, genomic characteristics, diversity of rearrangement, and phylogenetic inference [2,3,4,5,6,7,8,9,10,11,12,13,14,15,16,17,18,19,20,21,22,23]. 

**Figure 1:**
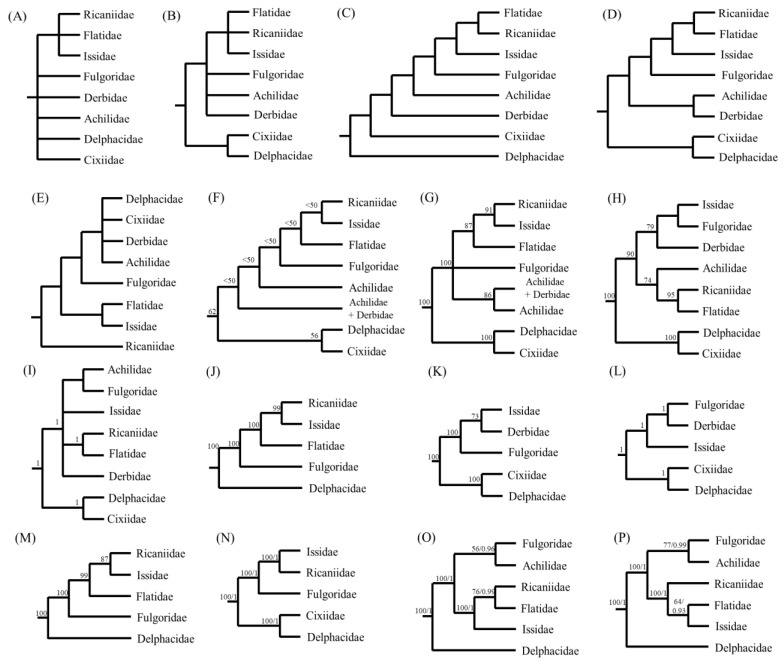
Alternative hypotheses of the familial relationships in Fulgoroidea. Trees are simply redrawn, and branch lengths are not to scale. (**A**) Muir [24] based on the number of spines on the second segment of the hind tarsi. (**B**) Asche [25] based primarily on adult morphological characteristics, including features of the female genitalia. (**C**) Emeljanov [26] based on adult and larval morphology. (**D**) Bourgoin [27] based on adult female genitalia. (**E**) Chen and Yang [28] based on larval metatarsi. (**F**,**G**) Urban and Cryan [29] based on *18S rDNA*, *28S rDNA*, *Histone3*, and *Wingless* using the Parsimony method and Bayesian inference (BI) method, respectively. (**H**,**I**) Song and Liang [30] based on *18S rDNA, 28S rDNA, 16S rDNA*, and *CytB* using the Maximum Likelihood (ML) and BI methods, respectively. (**J**) Zhang et al. [11] based on 13 protein-coding genes (PCGs) of mitochondrial genomes (mitogenomes), using the Neighbor-Joining method. (**K**,**L**) Song et al. [15] based on 13 PCG, 22 tRNA, and two rRNA of mitogenomes, using the ML and BI methods, respectively. (**M**) Huang and Qin [13] based on 13 PCGs of mitogenomes using the ML method. (**N**) Yu and Liang [16] based on 13 PCGs of mitogenomes using the ML and BI methods. (**O**) Wang et al. [21] based on 13 PCGs of mitogenomes using the ML and BI methods. (**P**) Xu et al. [22] based on 13 PCGs of mitogenomes using the ML and BI methods.

For the illustration of phylogenetic relationships among fulgoroid families, a diverse set of morphological characteristics has been scrutinized, including the number of spines on the second segment of the hind tarsi [24], primarily for adult morphology, including the female genitalia [25], adult and larval morphology [26], adult female genitalia [27], and larval metatarsi [28]. Additionally, multiple genes, such as *18S rRNA, 28S rRNA*, *Histone3*, *Wingless* [29], *18S rRNA*, *28S rRNA*, *16S rRNA*, and *CytB* [30] have been used to infer phylogenetic relationships in the Fulgoroidea. Furthermore, mitogenome-based analyses have also been performed in several studies with varying degrees of ingroup diversity, mainly using 13 protein-coding gene (PCG) sequences [11,13,15,16,21,22]. These studies have greatly improved our understanding of the phylogenetic relationships of fulgoroid families, but additional studies are still required, particularly those that investigate conflicting relationships and include a diverse taxonomic group (Figure 1).

The flatid planthopper, *Metcalfa pruinosa* (Hemiptera: Flatidae), is a polyphagous species that causes economic and aesthetic damage to diverse types of plants and is one of the most common species in eastern North America [31,32]. However, it was accidentally introduced to European countries, Russia, and Australia [33,34] and was also recorded in South Korea in 2005 [35]. *Salurnis marginella* (Hemiptera: Flatidae) is distributed in several Asian countries, and it invaded South Korea in 2013 from southwest China [36,37]. Currently, the damage caused by the species is negligible due to its small population size, but ecological adaptations to South Korean ecosystems are seriously concerning [38]. 

In this study, we determined the complete mitogenome sequences from two individuals of *M. pruinosa* and one of *S. marginella*. The two mitogenome sequences of *M. pruinosa* represent two haplotypes on the basis of the DNA barcoding sequences [39]. Currently, a mitogenome sequence is available for only for a single genus and species of Flatidae (*Geisha distinctissima*) [5]. Therefore, these three newly sequenced mitogenomes were incorporated into 42 available mitogenome sequences, representing eight families, to address several phylogenetic issues, including the phylogenetic position of Flatidae and familial relationships in Fulgoroidea. Furthermore, the genomic sequences were compared to those of other species of Fulgoroidea to better understand the evolutionary characteristics of Fulgoroidea, including gene rearrangement dynamics. Finally, intraspecies sequence divergence of each gene, including the DNA barcoding region, was categorized to identify suitable genes as molecular markers for population-level studies. 

## 2. Materials and Methods

### 2.1. Next-Generation Sequencing for M. pruinosa

Each individual of *M. pruinosa* found to possess different haplotypes (H1 and H3) from DNA barcoding sequencing [39] was subjected to next-generation sequencing, whereas *S. marginella* was sequenced by the Sanger method. The individual with a H1 haplotype was collected from the Korean locality of Gimhae, Gyeongsangnam-do Province, Republic of Korea, located in the south eastern region of Korea (35°16′41.6′′ N, 128°42′57.9′′ E) and that with a H3 haplotype was collected from Montpellier in France (43°36′36.0′′ N, 3°52′23.9′′ E). *S. marginella* was collected in Gimje, Jeollabuk-do Province, Republic of Korea, located in the southwestern region of Korea (35°48′41.3′′ N, 126°54′01.3′′ E). These specimens were deposited as voucher specimens at the Chonnam National University, Gwangju, Korea, under accession nos. CNU8050 and CNU8035 for *M. pruinosa* and CNU8352 for *S. marginella*. 

For library construction, approximately 40 ng of genomic DNA were isolated and randomly sheared using the Covaris System (Woburn, MA, USA) to generate inserts of ~300 bp fragments. Library construction was performed using the TruSeq Nano DNA Kit (Illumina, San Diego, CA, USA) following the manufacturer’s guidelines. Quality and DNA size of libraries were assessed using Agilent 2100 BioAnalyzer (Agilent Technologies, Palo Alto, CA, USA). Libraries were quantified by qPCR using the CFX96 Real Time System (BioRad, Hercules, CA, USA). After normalization, sequencing of the prepared library was conducted on the Illumina MiSeq platform using a Mid Output v2 kit to produce 150 bp paired-end reads (Illumina, San Diego, CA, USA).

Quality analysis of the raw sequence data was performed using FastQC software [40]. Adapter sequence reduction and trimming of low quality 5′- and 3′-ends of the reads were performed using Skewer ver. 0.2.2. [41]. Base-calling errors or insertions/deletions (indels) were corrected from the filtered set of reads using the alignment-based error correction tool Karect [42]. Consequently, ~1.45 Gb of nucleotides from 2.4 million reads for the Gimhae sample and ~1.63 Gb of nucleotides from ~2.87 million reads for the Montpellier sample were obtained. The Phred quality score (Q) indicated that base call accuracy was 86% for the Gimhae sample and 87.2% for the Montpellier sample from the Q30 score.

### 2.2. Assembly and Gap Filling

The two *M. pruinosa* mitogenomes were assembled from the Illumina reads using a baiting and iterative mapping approach with the software MITObim ver. 1.9 [43]. The assembled mitogenomes were remapped with the whole genome sequence reads using Bowtie2 [44] before conducting manual curation. Mismatch calling and correction of the assembled sequences were conducted using GATK [45]. Finally, primarily annotation of PCGs, tRNAs, rRNAs, and the A+T-rich region of each mitogenome was carried out using MITOS WebServer [46] (http://mitos.bioinf.uni-leipzig.de/index.py, accessed on 9 September 2021). 

For gap filling, two long overlapping fragments (LF1 and LF2) encompassing *COI* to *CytB* and *CytB* to *COI* were amplified, then each five (gap 1–gap 5) and two short fragments (SFs) (gap 2 and gap 3) for H1 and H3 haplotypes, respectively, were individually amplified using the primers designed in this study (Table S1). PCR was conducted using AccuPower^®^ PCR PreMix (Bioneer, Daejeon, South Korea) under the following conditions: denaturation for 5 min at 94 °C; 30 cycles of 1 min denaturation at 94 °C; 1 min annealing at 48–52 °C; 1 min extension at 72 °C; and a final extension of 7 min at 72 °C. Except for gap 2, the remaining gap regions were cloned after PCR amplification for sequencing. Cloning was carried out using a T-Blunt^TM^ PCR Cloning Kit (SolGent Co., Daejeon City, South Korea) and DH5α competent cells (Real Biotech Co., Banqiao City, Taiwan). The resultant plasmid DNA was isolated using an AccuPrep^®^ Plasmid Mini Extraction Kit (Bioneer Co., Daejeon City, South Korea). DNA sequencing was conducted using the ABI PRISM^®^ BigDye^®^ Terminator v3.1 Cycle Sequencing Kit and an ABI PRISMTM 3100 Genetic Analyzer (PE Applied Biosystems, Foster City, CA, USA). All products were sequenced from both directions.

### 2.3. S. marginella Sequencing by the Sanger Method

For *S. marginella*, a hind leg was used to extract DNA using a Wizard Genomic DNA Purification Kit (Promega, Madison, WI, USA) according to the manufacturer’s instructions. Four primer sets that amplify four long overlapping fragments (Table S2; LF1, LF2, LF3, and LF4) were designed using previously reported mitogenome sequences of *G. distinctissima* [5] and the two current *M. pruinosa*, all of which belonged to the family Flatidae: LF1, LF2, LF3, and LF4 amplified *COI* and *ND3* (~3.7 kb), *COIII* to *ND4* (~3.7 kb), *ND5* to *srRNA* (5.3 kb), and *lrRNA* to *COI* (~3.8 kb), respectively. Amplification of the LFs was conducted using LA Taq^TM^ (Takara Biomedical, Tokyo, Japan) under the following conditions: 96 °C for 2 min, 30 cycles of 98 °C for 10 s and 48 °C for 15 min, and a final extension step of 72 °C for 10 min. Thereafter, these amplicons were used as templates to amplify 30 overlapping SFs using AccuPower^®^ PCR PreMix (Bioneer, Daejeon, South Korea) under the following conditions: initial denaturation for 5 min at 94 °C, followed by 35 cycles of 30 s at 94 °C, 1 min at 48–52 °C, and 1 min at 72 °C, and a final 7 min extension at 72 °C. The primers for SFs were also designed using *G. distinctissima* and the two *M. pruinosa* (Table S2). Individual SF sequences were assembled manually into the complete mitogenome using SeqMan (DNASTAR, Madison, WI, USA). 

### 2.4. Gene Annotation

Annotations were performed using MITOS WebServer (http://mitos.bioinf.uni-leipzig.de/index.py, accessed on 9 September 2021) with the search mode set as default, Mito/Chloroplast set as the searching source, and the genetic code of invertebrate mitogenomes set for tRNA isotype prediction [46]. Overall, 21 tRNA genes were identified, and the boundaries were delimitated based on these parameters. However, *trnS*_1_, which has a truncated dihydrouridine (DHU) arm, was detected using a hand-drawn secondary structure in conjunction with an alignment of the predicted *trnS_1_* regions of co-familial species *G. distinctissima* [5], and the anticodon was given particular consideration [7,11]. Start and stop codons of PCGs were further confirmed by alignment against mitochondrial (mt) PCGs of the fulgoroid species [4,5,7,14]. The nucleotide sequences of the PCGs were translated based on the invertebrate mitochondrial DNA (mtDNA) genetic code. Sequence data were deposited into the GenBank database under accession nos. MK303326 and MN417319 for H1 and H3 haplotypes of *M. pruinosa*, respectively, and MT628542 of *S. marginella*.

### 2.5. Comparative Genome Analyses

For the comparative analysis, 42 fulgoroid mitogenome sequences, which represent 27 species (including unidentified species) of 20 genera in 10 subfamilies of eight families, were downloaded from the GenBank database. The mitogenome sequences lacking generic names and a substantial genic sequence were excluded from genomic comparison and phylogenetic analysis. Further, among the 81 mitogenome sequences of *L. striatellus* reported by Sun et al. [2], only two representing each haplotype group were included. These sequences, along with the three mitogenome sequences obtained in the present study, were compared for several genomic characteristics. The A+T content of each gene, whole genome, and codon position of the PCGs were calculated using DNASTAR (Madison, USA). Codon usage was determined by MEGA 6 [47], and the gene overlap and intergenic space sequences were hand counted. The genetic distance at each taxonomic category was calculated using unrooted pairwise distance using PAUP ver. 4.01b10 [48]. These values were plotted using boxplots implemented in JMP software ver. 11.1.1 (SAS Institute, Cary, NC, USA). 

Compositional skewness, which measures the relative number of As to Ts [AT skew = (A − T)/(A + T)] and Gs to Cs (GC skew = [(G − C)/(G + C)]), was calculated to determine the base composition of nucleotide sequences [49]. Ka and Ks, along with the Ka:Ks ratio, were estimated to determine the degree of genetic divergence of the whole genome, PCGs encoded in each strand, each individual PCG in fulgoroid species, and each PCG in each family using a model that averages parameters across 14 candidate models [50] with the KaKs Calculator ver. 1.2 [51]. *Thrips imaginis* in the order Thysanoptera [52] was used as a criterion sequence for calculations. Subsequently, a series of one-way analyses of variance (ANOVAs) and Tukey’s honestly significant difference multiple range tests were performed using JMP software ver. 15.10 (SAS Institute) to detect the statistical difference of the mean Ka/Ks values obtained by pairwise comparisons. 

### 2.6. Phylogenetic Analysis

For the phylogenetic reconstruction of the superfamily Fulgoroidea, the nucleotide sequence of each PCG was aligned based on the codons using RevTrans ver. 2.0 [53]. The well-aligned blocks within each PCG were selected using GBlocks 0.91b 9 [54], with the maximum number of contiguous non-conserved positions set to 11. Gap positions were excluded within the final blocks. Each of the 11 aligned PCGs (excluding ND1 and ND3, which are unavailable in some species) was then concatenated to generate the nucleotide (NU) sequences of the PCG dataset (7716 bp excluding gaps for the NU sequence dataset). For amino acid (AA) sequence-based analysis, the NU sequences of the 11 PCGs were recorded into AA sequences using RevTrans ver. 2.0 [53], and these were concatenated into a single data matrix (2271 AAs including gaps for the AA dataset).

PartitionFinder2 was used to search for the optimal partitions and the corresponding optimal models of substitution using the ‘greedy’ search [55,56,57], with the inclusion of the evolutionary models available in RAxML [58] and MrBayes [59]. As a result, five partition schemes for the NU data matrix were obtained, providing three different substitution models (GTR + I + G for subset 1, 2, and 4; TVM + Gg for subset 3; and HKY + G for subset 5), and two partition schemes for the AA data matrix were obtained, providing two different substitution models (MTART + I + G + F for subset 1 and MTZOA + I + G + F for subset 2). These partition schemes and substitution models for each data matrix were applied for each phylogenetic analysis. 

To reconstruct the phylogeny of the Fulgoroidea, we used both the maximum likelihood (ML) and Bayesian inference (BI) algorithms using RAxML ver. 8.2.10 [58] and MrBayes ver. 3.2.7 [59], respectively, implemented within the CIPRES Portal ver. 3.1 [60]. For BI analysis, two independent runs of four incrementally heated Markov and Monte Carlo chains (one cold chain and three hot chains) were simultaneously run for 10 million generations, with tree sampling conducted at every 500 generations. The first 25% of the sampled trees were discarded as burn-in. Partitioned analyses were conducted with each partition unlinked in each parameter (revmat, statefreq, shape, pinvar, and tratio). An average split frequency of less than 0.01 was used to represent the convergence of the two simultaneous runs. For ML analysis, the RAxML algorithm was applied, which uses a “rapid” bootstrapping approach and searches for the best-scoring tree [58]. Confidence values for BI trees were obtained from the Bayesian posterior probabilities (BPPs), and those for ML trees were determined with 1,000 bootstrap (BS) iterations. *Durgades nigropicta* and *Populicerus populi* from another infraorder Cicadomorpha, which has traditionally been known as the sister group to Fulgoroidea in Auchenorrhyncha, were selected as outgroups [61,62]. The phylogenetic trees were visualized using iTOL ver. 4 [63].

## 3. Results and Discussion

### 3.1. General Mitochondrial Genome Features

The three mitogenomes contained 37 typical genes (13 PCGs, 22 tRNA genes, and two rRNA genes) and one non-coding A+T-rich region, found in animals [64]. The mitogenome sizes of the two *M. pruinosa* haplotypes (H1 and H3) and *S. marginella* were 16,312, 16,314, and 16,126 bp, respectively, which were well within the range previously reported for complete mitogenomes of Fulgoroidea, and the same is true for the number of codons of 3656, 3656, and 3637, respectively, excluding termination codons (Table 1; Table S3). The A/T nucleotide composition of the whole genome was 76.61%, 76.66%, and 75.73% in the *M. pruinosa* H1, *M. pruinosa* H3, and *S. marginella* mitogenomes, respectively, indicating biased A/T nucleotides. The A/T content among *M. pruinosa* genes and region was the highest for *lrRNA*, 79.53% and 79.53%; followed by *srRNA*, 77.96% and 77.96%; the A + T-rich region, 77.46% and 77.54%; tRNAs, 76.82% and 76.82%; and PCGs, 75.78% and 75.79%, in H1 and H3, respectively. However, this trend was detected only in the *M. pruinosa* mitogenomes in the Fulgoroidea, presenting a diverse pattern in the Fulgoroidea, even in the co-familial species *S. marginella* and *G. distinctissima* (Table 2).

The biased A/T content was reflected in the form of codon usage. *M. pruinosa* H1, *M. pruinosa* H3, and *S. marginella* PCGs had TTA (leucine), ATT (isoleucine), TTT (phenylalanine), and ATA (methionine) as the four most frequently used codons with frequencies of 35.61%, 35.64%, and 34.78%, respectively, among the 64 available codons and the frequency of the co-familial species *G. distinctissima* was the lowest at 32.01% (Table S4). A similar pattern was also found in all sequenced Fulgoroidea, ranging in frequency from 30.14% (*Magadhaideus luodiana*) to 40.45% (*Saccharosydne procerus*) (Table S4). These four codons comprised A or T nucleotides, indicating the biased usage of A/T nucleotides in Fulgoroidea.

The two haplotypes of *M. pruinosa* shared an identical start codon, and *S. marginella* used different codons to those of *M. pruinosa* in *COI*, *ND3*, and *ND5* (e.g., ATC in *M. pruinosa* vs. ATG in *S. marginella* for *COI*). All fulgoroid species, including the three current mitogenomes, had the canonical start codon ATN, but one species in Delphacidae (*Ugyops* sp.) used the atypical codon CTG for *COI* (Table 2; Table S3). Previously, the insect orders Lepidoptera and Coleoptera have often shown non-canonical start codons, such as GCA in Lepidoptera [65] and AAT/AAC in Coleoptera for *COI*, but CTG is highly unconventional. GTG, which is not typical, but is a canonical start codon in insects, was also used in several fulgoroid species for *ND5* and *ND1* (Table S3). Examples of the GTG start codon in insects are found in *Drosophila formosana* in Diptera for *ND5* [66] and *Saturnia jonasii* in Lepidoptera for *COII* [65]. 

**Figure 2:**
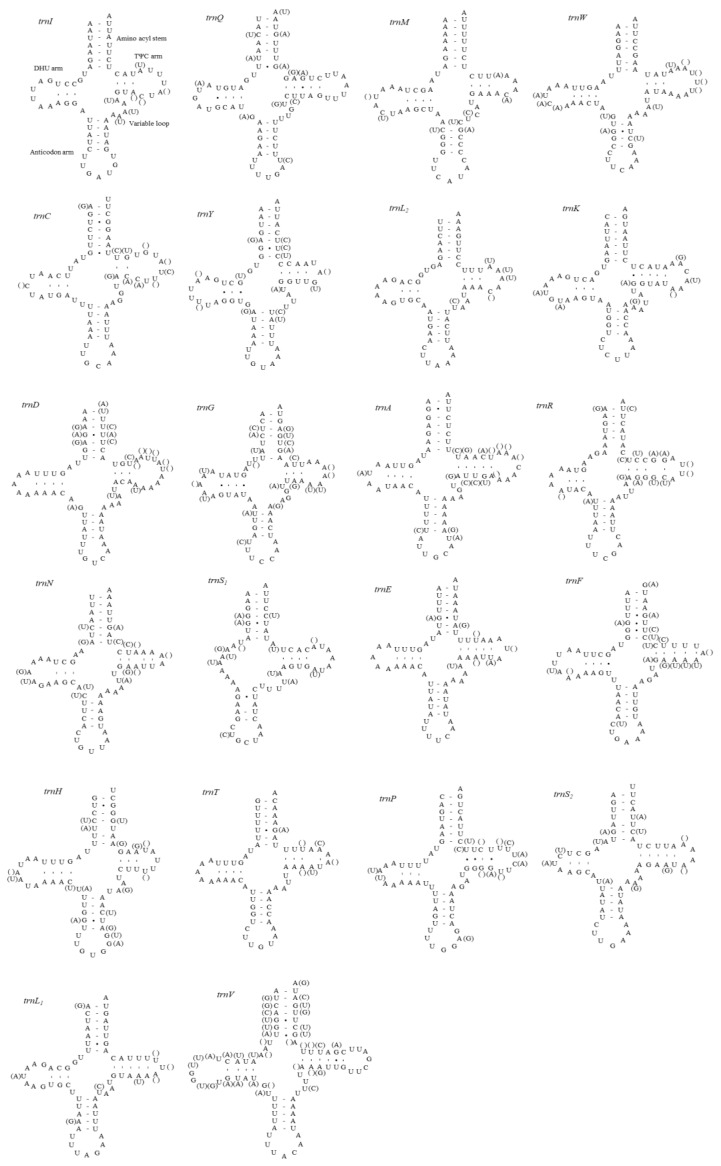
Predicted secondary cloverleaf structure for the 22 tRNA genes of *Metcalfa pruinosa* haplotypes and *Salurnis marginella*. Hyphens (-) indicate Watson–Crick base-pairing, and centered dots (•) indicate G–U base pairing. The arm of tRNAs (clockwise from top) is the amino acid (AA) acceptor, TΨC (T), anticodon (AC), and dihydrouridine (DHU) arm. Within parentheses are sequences for *S. marginella*.

A total of 22 tRNA genes (one specific for each amino acid and two for leucine and serine) were identified for each mitogenome sequenced in this study (Figure 2). All tRNAs except *trnS_1_*, which lacked the DHU loop, were shown to be folded into the expected cloverleaf secondary structures. In other fulgoroid species, which are available for their tRNA structure *trnS_1_*, were also truncated at the DHU loop (11 mitogenomes in 10 species) [6,9,16,21,22]. This incomplete *trnS_1_* structure has been detected in the mitogenomes of other animals, including insects [67]. The lengths of *trnS_1_* of the 11 fulgoroid species, including those of current studies, were nearly always the shortest among tRNA isotypes, ranging in size from 54 to 63 bp, due to incomplete formation of the DHU loop, whereas the longest tRNA was variable as *trnV*, *trnK*, *trnW*, or *trnD*, ranging in size from 71 to 73 bp (Table S5). The postulated tRNA cloverleaf structure of *M. pruinosa* (H1 and H3) and *S. marginella* harbors an invariable 7 bp in the aminoacyl stem, 5 bp in the anticodon stem, and 7 bp in the anticodon loop, whereas the DHU arm and the TΨC arm, particularly within the loops, were variable in length (Figure 2). A total of 51 and 45 unmatched base pairs were detected in the *M. pruinosa* (H1 and H3) and *S. marginella* tRNAs, respectively, but 31 and 24, respectively, were G-U pairs, which form a weak bond in the tRNAs. The remaining mismatches were found either in the aminoacyl stem (nine in *M. pruinosa* and *S. marginella*), DHU stem (zero in *M. pruinosa* and one in *S. marginella*), anticodon stem (eight in *M. pruinosa* and *S. marginella*), or TΨC stem (three in *M. pruinosa* and *S. marginella*), indicating that the amino-acyl stem has the highest mismatch. In each tRNA the number of mismatches ranged from one in *M. pruinosa* to three in *S. marginella* tRNAs. 

### 3.2. Compositional Skew

**Figure 3:**
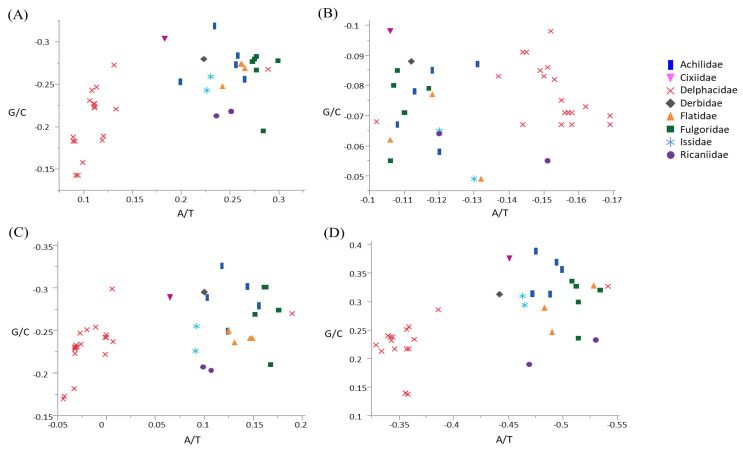
Scatter plot of A/T and G/C skews of the fulgoroid species, including *Metcalfa pruinosa* and *Salurnis marginella*. (**A**) Whole mitochondrial genome, (**B**) 13 protein-coding genes (PCGs), (**C**) nine majority-strand encoded PCGs (*ND2*, *COI*, *COII*, *ATP8*, *ATP6*, *COIII*, *ND3*, *ND6*, and *CytB*), and (**D**) four minority-strand encoded PCGs (*ND5*, *ND4*, *ND4L*, and *ND1*). X-axis and Y-axis indicate A/T skewness and G/C skewness, respectively.

Skewness was calculated to infer the compositional bias of base in terms of whole mitogenome, whole PCGs, major-strand PCGs, and minor-strand PCGs in Fulgoroidea (Figure 3; Table S6). The three mitogenomes and co-familial species *G. distinctissima* in Flatidae (measured from the major strand) was slightly A-skewed (ranged from 0.242 to 0.265) and moderately C-skewed (−0.274–−0.248) in the whole genome (Figure 3; Table S6). Within fulgoroid families and subfamilies, all species of Delphacinae in Delphacidae, excluding another subfamily, Asiracinae, were obviously less A-skewed (0.089–0.133) compared to other families, including the Flatidae (0.183–0.299), suggesting a different evolutionary force even within fulgoroid families (Figure 3). Variability in skewness has also been found in other taxonomic groups of Hemiptera. For example, the superfamily Membracoidea in the infraorder Cicadomorpha in Auchenorrhyncha showed variable degrees of A- or T-skewness and G- or C-skewness [68], whereas the superfamily Aphidoidea in another suborder Sternorrhyncha showed A- and C-skewness in the whole genome [69], as seen in Fulgoroidea (Figure 3). 

When the degree of base bias was calculated in different strands of Fulgoroidea species, including current *M. pruinosa* and *S. marginella*, the major strand that encodes for nine PCGs (*ND2*, *ND3*, *ND6*, *COI*, *COII*, *COIII*, *ATP6*, *ATP8*, and *CytB*) exhibited a slight to moderate A-skewness in all species that were not of the subfamily Delphacinae (0.065–0.190), whereas all members of Delphacinae were slightly T-skewed (−0.044–0) (Figure 3). On the other hand, the minor strand, which encodes for four PCGs (*ND1*, *ND4*, *ND4L*, and *ND5*), evidenced a profound T-skewness in species that were not of the subfamily Delphacinae, including *M. pruinosa* and *S. marginella* (−0.541–−0.442), but all members of Delphacinae were less T-skewed (−0.386–−0.329). Similarly, whole PCGs showed different evolutionary bias in Delphacinae compared to other families. However, with regard to GC-skewness, the major strand PCGs of all fulgoroid species evidenced moderate to strong C-skewness, whereas those of the minor strand exhibited moderate to strong G-skewness, without an obvious difference between species that were not of the subfamily Delphacinae and those that were. Therefore, the degree of bias differed between the two strands and among taxonomic groups, although such strand-based inequalities in base frequencies have yet to be fully understood. 

### 3.3. Genetic Divergence Inferred from the Ka/Ks Ratio

**Figure 4:**
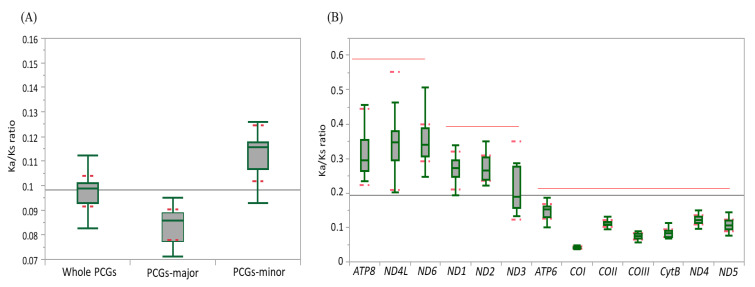

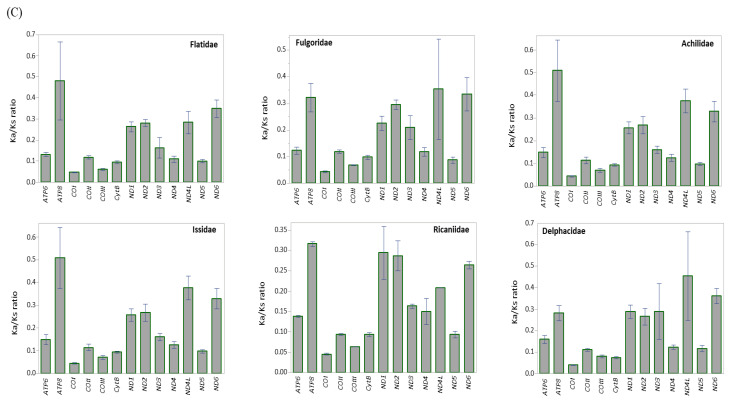
The mean scores of the Ka:Ks ratio of 13 protein-coding genes (PCGs) in 45 mitochondrial genomes of Fulgoroidea. (**A**) Whole PCGs, major-strand encoded PCGs, and minor-strand encoded PCGs. Dotted lines indicate standard deviation. (**B**) Each individual PCG. Dotted and un-dotted horizontal lines indicate standard deviation and statistically identical groups, respectively. (**C**) Each family. Vertical lines indicate standard deviation.

Ka and Ks were estimated to infer the extent of genetic divergence in terms of whole PCGs, major-strand PCGs, minor-strand PCGs, and individual PCGs in Fulgoroidea (Table S7). The ratios of Ka:Ks were well below 1 for all species, including *M. pruinosa* and *S. marginella* in whole PCGs (0.1122 at maximum in *Sivaloka damnosus* in Issidae), major-strand PCGs (0.0953 at maximum in *Pyrops candelaria* in Fulgoridae), minor-strand PCGs (0.1566 at maximum in *S. damnosus* in Issidae), and individual PCGs (Figure 4; Table S7), suggesting that the PCGs in Fulgoroidea are under purifying selection. The mean Ka:Ks ratio was higher in the order of minor-strand PCGs, whole PCGs, and major-strand PCGs in Fulgoroidea, and the ANOVA test (Table S8) showed a significant difference among them at the level of *p* < 0.0001. Individual gene analysis provided three statistically different groups in the mean Ka:Ks ratio: *ATP8*, *ND4L*, and *ND6* as the highest group; *ND1*, *ND2*, and *ND3* as the next highest group; and the remaining genes as the lowest group (Figure 4B). Such different divergence patterns among PCGs might be useful for different purposes, depending on the degree of necessary variability, such as higher variability for species delimitation and conserved variation for the discrimination of higher taxonomic groups. 

At the familial analysis, Flatidae, which comprises *M. pruinosa* and *S. marginella*, also showed a similar pattern to that of the whole fulgoroid species, in that *ATP8*, *ND4L*, and *ND6* showed the highest mean Ka:Ks ratio, *ND1*, *ND2*, and *ND3* showed the next highest ratio, and the remaining genes showed the lowest mean Ka:Ks ratio, but no group of genes differed with statistical significance (Figure 4C; Table S8). Similar patterns were also observed in Fulgoridae, Achilidae, and Issidae, but Ricaniidae included *ND1*, *ND2*, and *ATP8* as the highest group, instead of *ND4L* and *ND6*, without statistical significance, and Delphacidae included *ND3* along with *ND4L* and *ND6* as the highest group, instead of ATP8, with statistical support only in *ND4L* and *ND6* (Figure 4C; Table S8). These results indicate that the evolution of fulgoroid families is largely consistent but also has distinctions in certain families, such as Ricaniidae and Delphacidae. 

### 3.4. Individual Gene Divergence within Species 

One of the main utilities of mtDNA sequences includes the property of *COI* sequences as a “DNA Barcode” [70]. Further, this region has been recommended for early insight into the patterning of genomic diversity within species [71]. In fact, the barcode region in *COI* has been used extensively to understand geographic variation and the origin of invasion of species [72,73,74,75]. Nevertheless, low variability and low numbers of haplotypes have necessitated additional sequence regions that can be used independently to barcode regions or in combination with barcode regions for the detection of population structures and hidden lineages [72,75,76], including introduced species [39,77,78,79]. 

**Figure 5:**
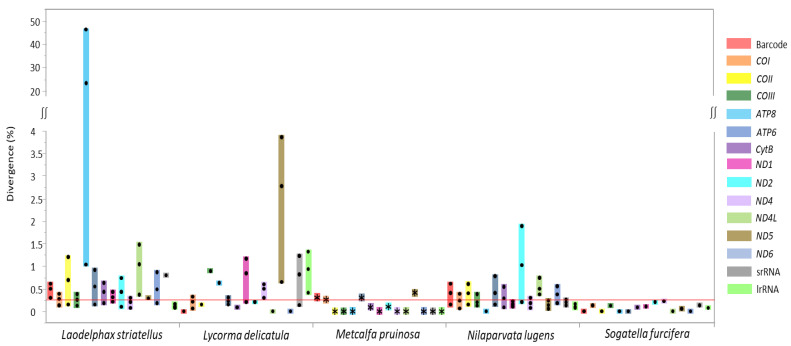
Boxplot distribution of intraspecies genetic divergence for 12 protein-coding genes (excluding *ND3* gene), the barcode region, and two rRNAs in fulgoroid species available for multiple genome sequences. Dots in the colored bars indicate maximum, average, and minimum divergence (%), respectively; and the reddish horizontal line represents the median of sequence divergence (%) in Fulgoroidea.

In order to quantify sequence divergence at each species, a pairwise comparison of each gene, including the DNA barcoding region, was performed from the species available for multiple genome sequences (six sequences for *L. striatellus*, three for *Lycorma delicatula*, eight for *Nilaparvata lugens*, and two for *Sogatella furcifera*), including two *M. pruinosa* haplotypes (Figure 5). In the case of *M. pruinosa*, *CytB*, *ND2*, *COI*, DNA barcoding region, *ATP6*, and *ND5* provided variation in the order of the lowest to highest of average median value (0.089–0.417), with the exclusion of *ND3* due to unavailability in some other species (Figure 5). Therefore, *COI*, which includes the DNA barcoding region, was not the most variable region. Similarly, other species also provided genes with higher variability than the DNA barcoding region, with varying numbers of genes (Figure 5). These more variable genes might be informative for intra-specific analysis for diverse purposes. 

### 3.5. Intergenic Spacer Regions and Potential Motif Sequences

**Figure 6:**
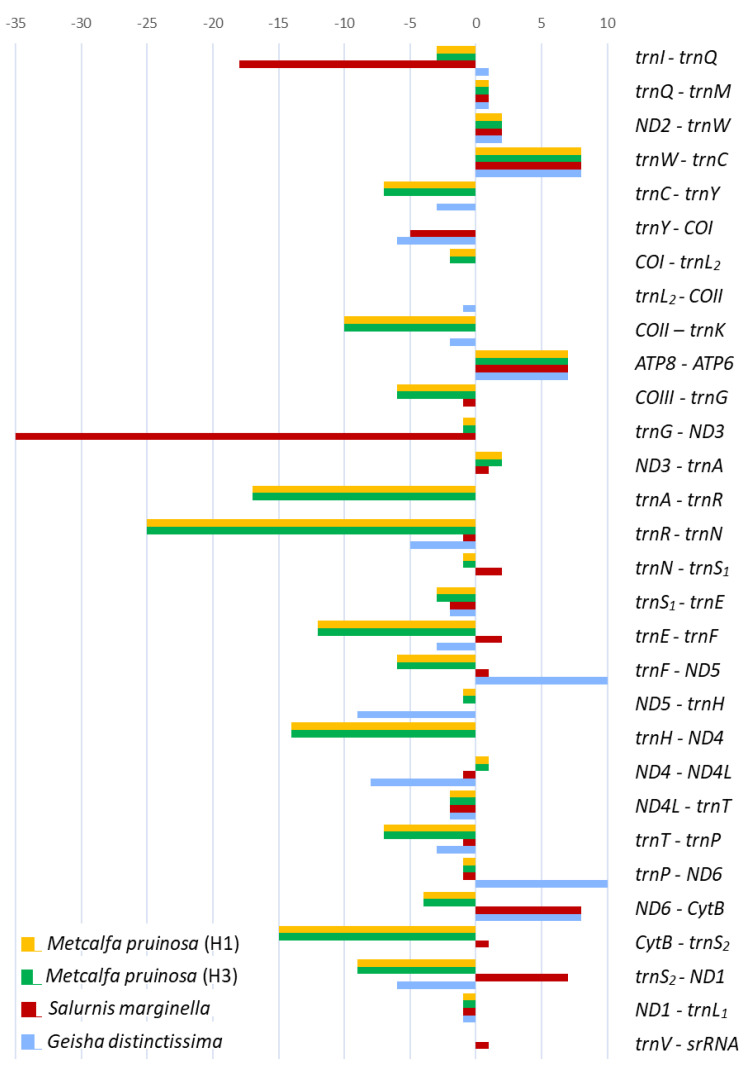
Lengths of overlapping sequences (negative values) and intergenic spacer sequences (positive values) of mitochondrial genomes of Flatidae, including *Metcalfa pruinosa* and *Salurnis marginella*.

The genes of the two *M. pruinosa* haplotypes and *S. marginella* are interleaved with 147 and 68 bp, which are spread over 21 and 11 regions, ranging in size between 1 and 25 and 1 and 35 bp, respectively (Figure 6; Table S9). The majority of intergenic spacer sequences (ISSs) are short (1–2 bp), but five and two locations in *M. pruinosa* and *S. marginella*, respectively, have ISSs that are longer than 10 bp. The longest, 25 bp, located between *trnR* and *trnN* in the two *M. pruinosa* haplotypes, which both have two identical poly-runs of adenine nucleotides, is composed of 84% A/T nucleotides. The next longest, 17 bp, ISS is located between *trnA* and *trnR* and also has poly-running adenine nucleotides, which also have higher A/T nucleotides. In *S. marginella*, the longest 35-bp-long ISS is located between *trnG* and *ND3*, but it has interspersed poly-running TAA sequences composed of 91.4% A/T nucleotides and the next longest, 18 bp, is located between *trnI* and *trnQ*. On the other hand, the co-familial species *G. distinctissima* does not have ISSs that are longer than 10 bp in the 51 bp of ISSs that are interspersed at 13 locations, indicating that there is no consistent location and length of ISSs among Flatidae species. 

In other fulgoroid families, the total length of ISSs tended to be longer than those of Flatidae, providing 55–1,090 bp with a few exceptions (Table S9). In the case of *Peltatavertexalis horizontalis* in Achilidae, one spacer located between *lrRNA* and *trnV* was exceptionally long at 1,008 bp. Except for this extreme case, all members of Delphacinae in Delphacidae were obviously longer than any other members of Delphacidae, ranging from 178 to 856 bp, with an exception of 131 bp found in *L. striatellus* collected in China [4], indicating different genomic evolution in Delphacinae than other fulgoroid families. 

**Figure 7:**
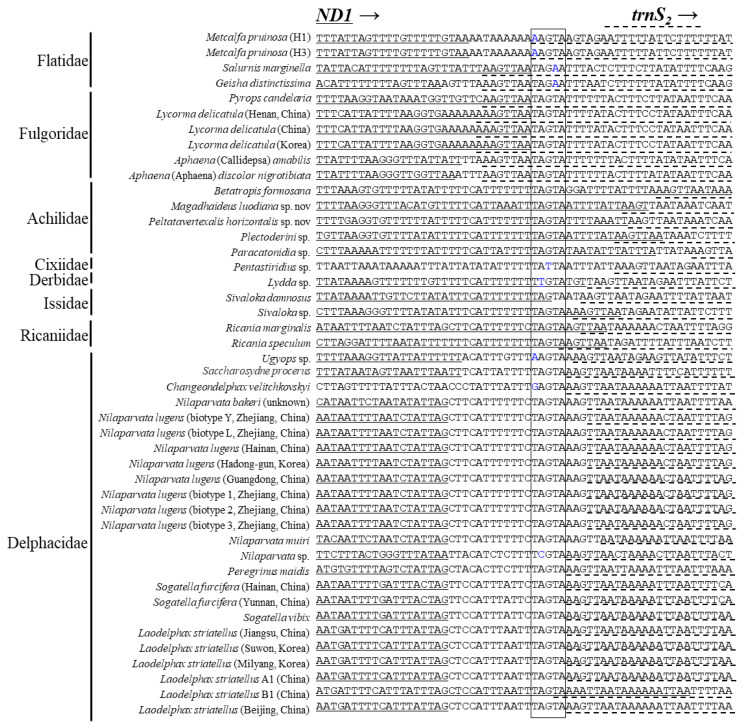
Alignment of the junction between the 3′-end of *ND1* and 5′-end of *trnS*_2_ in fulgoroid species. The boxed nucleotides indicate the conserved pentanucleotide (TAGTA) detected in the fulgoroid species. Underlined and dotted nucleotides indicate the adjacent partial sequences of *ND1* and *trnS_2_*, respectively. Arrows indicate the transcriptional direction. Blue-colored nucleotides indicate variation from conserved pentanucleotides.

It has been reported that an intergenic spacer region located at the *ND1* and *trnS_2_* junction contains a conserved motif sequence as TTAGTAT in Lepidoptera and TAGTA in Coleoptera with a slight modification in some species [65,80,81]. Such motif sequences have been signified for their role as binding sites for the transcription termination peptide of mtDNA (mtTERM) because the sequence signals the termination of the transcription of PCGs encoded on the major strand (*CytB*) in the circular mtDNA [82,83]. We searched the homolog in the Fulgoroidea genomes and found the motiflike sequence, TAGTA, at the *ND1* and *trnS_2_* junction (Figure 7). However, only the members of Delphacidae had a consistent length of ISS and contained the motiflike sequence within a well-aligned frame between the two genes, but a slight modification was also present in some species. On the other hand, the remaining family members lacked the ISS but had the motiflike sequence at the 3′-end of *ND1* or at the 5′-end of *trnS_2_*, with a slight modification in some species. A functional study for its role as mtTERM in Fulgoroidea will be necessary for further decisive conclusions on the role of the sequence.

### 3.6. A+T-rich Region Structure

Located between the *srRNA* and *trnI*, the A+T-rich region of fulgoroid species was mostly longer than that for other insect orders (e.g., ~350 bp in Lepidoptera), with the size well over 1000 bp, with only a few exceptions (Table 1). The two *M*. *pruinosa* haplotypes were 1788 (H1) and 1790 bp (H3), presenting a unique length difference in the whole genome, including genic regions and ISSs between them (Table 1). The *S*. *marginella* A+T-rich region was slightly longer at 1836 bp but was well within the range found in other fulgoroid species (Table S3). 

**Figure 8:**
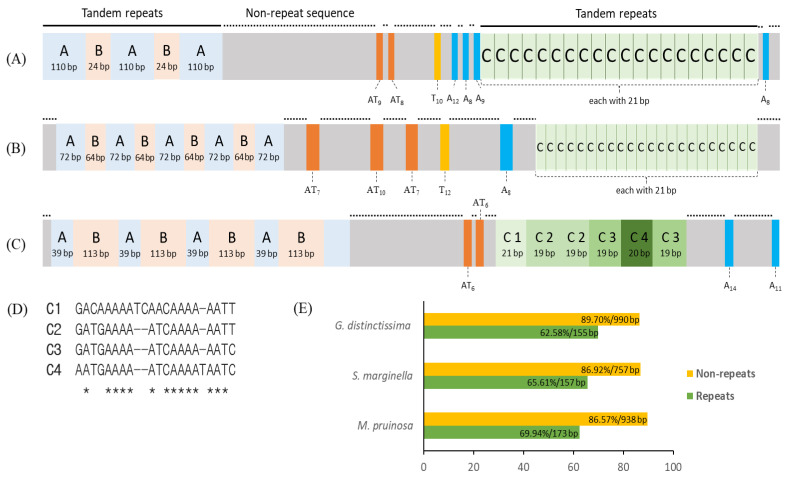
Schematic map of the A+T-rich region in Flatidae. (**A**) *Metcalfa pruinosa* H1 and H3, (**B**) *Salurnis marginella*, and (**C**) *Geisha distinctissima*. (**D**) An alignment of the repeat region C1-C4 of *G. distinctissima*, and (**E**) A/T content of each Flatidae species. Lines on the bars, longer repeat regions; dotted lines on the bars (on the gray boxes), nonrepeat sequences; orange boxes, the poly-AT repeats; yellow boxes, poly-T repeats; blue boxes, poly-A repeats; and *, consensus sequences.

The A+T-rich region of the two *M. pruinosa* is structured into four regions: one repeat region; a large nonrepeat region; another repeat region; and a short nonrepeat region from the 3′-end of *srRNA* to the 5′-end of *trnI* (Figure 8A). The first repeat region is composed of tandem triplicated repeats, each of which consists of two units (named repeat units A and B) that have an identical sequence and length, but the third copy lacks the repeat unit B. The second repeat region (named repeat unit C), which is located closer to *trnI*, is repeated 20 times, each with an identical 21 bp. A large nonrepeat region contains intermittent variable length poly-AT, poly-T, and poly-A that are scattered in the region. At the end of the A+T-rich region abutting to the 5′-end of *trnI*, one short, nonrepeat sequence, which contains one poly-A, was located. A similar structure was also found in *S*. *marginella* and *G. distinctissima*, in that they are all composed of four regions, but the copy number and repeat length differ between them (Figure 8B,C). In particular, the repeat C in *G. distinctissima*, composed of six copies, contains indels and substitutions among them, providing four different versions (Figure 8D). One of the common interpretations for the occurrence of such tandem repeats in animal mitogenomes is slipped-strand mispairing, in concert with unequal crossing over during DNA replication, resulting in an expanded repeat [84,85,86]. 

Due to the similarity in structural arrangement in the A+T-rich region among Flatidae species, the possibility of a common origin of each repeat unit was considered by sequence alignment, but no reasonable alignment was obtained (data not shown), suggesting an independent origin of each repeat unit in each species. Furthermore, an alignment of each repeat unit to its own mitogenome sequences, including ISSs, did not yield any fruitful results (data not shown), suggesting that the origin of the repeat units is probably elsewhere in the mitogenome. This inference is further supported by the substantially lower A/T composition of the repeat units than that of the nonrepeat regions (62.58–65.61% vs. 86.57–89.70%; Figure 8E), along with other genic and non-genic regions in the genome in each species (Table 1). In contrast to mitochondrion-to-nucleus transfer of DNA, such as nuclear-encoded mt pseudogenes [87,88,89,90], nucleus-to-mitochondrion transfer of DNA has not critically been examined in animals, including insects, and no citable example could be found. However, such an event can casually be exemplified in plant mitogenomes [91,92,93]. Therefore, the potential origin of the repeat units from chromosomal DNA cannot completely be excluded in insects, including Flatidae species. If no chromosomal sequences of any Flatidae species are available from a homology search of NCBI through BLAST, this may be limiting, particularly due to the lower ratio of query cover in many repeat units (e.g., 26% in repeat B in *G. distinctissima* and 29% in repeat A in *M. pruinosa*; Table S10). Nevertheless, several repeat units showed a substantial sequence homology to the chromosomal DNA originating from a diverse organism, where even their lengths were shorter, hinting at the potential origin of the repeat unit from other organisms during the long evolutionary history. For example, the repeat units A, B, and C of *M. pruinosa* showed 94.12% sequence identity to the chimpanzee *Pan troglodytes*, 100% to the radish *Raphanus sativus*, and 100% to the tapeworm *Spirometra erinaceieuropaei*, respectively, and a similar spectrum of homology was obtained from the repeat units of other Flatidae species (Table S10). As some repeat units showed higher sequence homology to parasites (e.g., tapeworms and bacteria), genomic transfer to mitogenomes via parasites can also be considered, although it is not decisive. Therefore, the evolutionary mechanism responsible for the incorporation of such repeat sequences in the A+T-rich region may require further close analysis with chromosomal genomic sequences. 

Previously, the detailed analysis of the A+T-rich region of the order Hemiptera classified the region into three compositional types and found several Fulgoromorpha species, including *G. distinctissima* and species of Cicadomorpha to have two repeat regions separated in the region [94], as we have shown for *G. distinctissima* in Figure 8C. Further, the stem-and-loop structure has often been detected in other Hemiptera species [94], but we could not casually locate such a structure in any species of Flatidae. Li and Liang [95] further scrutinized the A+T-rich region registered in the GenBank database, which covers 11 infraorders of Hemiptera, including Fulgoromorpha, and reported one longer poly-A stretch (23 bp) closer to the 3′-end of *srRNA*. However, in Flatidae, only *M. pruinosa* and *G. distinctissima* had such a poly-A stretch (Figure 8). Therefore, generalization of the structural arrangement in the A+T-rich region requires further data accumulation of Fulgoromorpha, particularly for Flatidae. 

### 3.7. Gene Arrangements 

**Figure 9:**
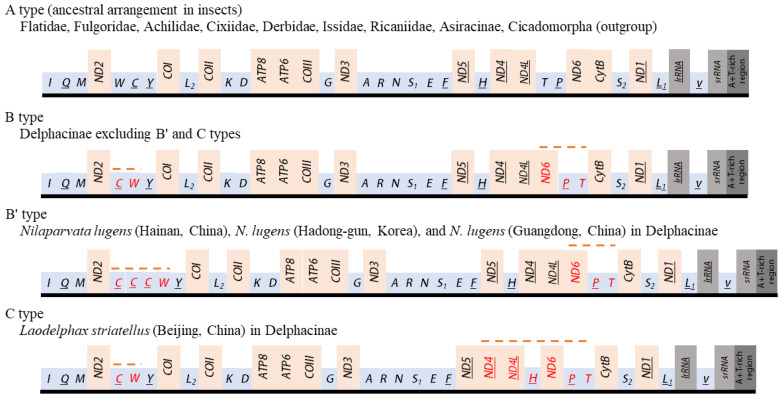
Schematic illustration of the available mitochondrial gene arrangements in Fulgoroidea. Gene sizes are not drawn to scale. Gene names that are not underlined indicate a forward transcriptional direction, whereas underlining indicates a reverse transcriptional direction. tRNAs are denoted by one-letter symbols in accordance with the IUPAC-IUB single-letter amino acid codes. Dotted lines above the gene names indicate rearranged genes relative to ancestral arrangement in insects.

The orientation and gene order of Flatidae species, including *M. pruinosa* and *S. marginella*, were identical to those of the species of Ricaniidae, Issidae, Achilidae, Fulgoridae, Derbidae, subfamily Asiracinae of Delphacidae, and the two outgroup species, which belong to another infraorder, Cicadomorpha (A type; Figure 9). This arrangement has been hypothesized to be ancestral for insects and found in diverse insect orders [64]. However, members of the Delphacinae in Delphacidae represented by nine species in seven genera are highly interesting in that members of the subfamily uniquely presented two different arrangements (B and C types). The C type arrangement was found only in one mitogenome of *L. striatellus* collected from Beijing, China [4], whereas other individuals of the same species [8,18,19], including 81 sequences of the species [2], along with the remaining species in the subfamily, all possessed the B type (Figure 9). Furthermore, among the eight *N. lugens* mitogenomes reported from China and South Korea each, two and one sequences from China and South Korea, respectively [8,10,17,19], presented a triplicated *trnC* at the *ND2* and *trnY* junction with the same rearrangement to B type (named B’ type), indicating intra-specific changes in gene arrangement or duplication (Figure 9). 

Compared to the insect ancestral type [64], two regions had rearranged genes in the B type. One rearranged region involved the translocation of *ND6* to a position upstream of *ND4L* (underlining indicates a gene inversion) and a translocation between *trnT* and *trnP* without gene inversion, resulting in *ND6*-*trnP*-*trnT*, instead of ancestral *trnT*-*trnP*-*ND6* arrangement at the *ND4L* and *CytB* junction. Another region involves transposition between *trnW* and *trnC*, resulting in the *trnC*-*trnW* arrangement at the *ND2* and *trnY* junction. In addition, the C type arrangement has a translocation of *trnH* from the *ND5* and *ND4* junction to the upstream region of *ND6*, resulting in *trnH*-*ND6*-*trnP*-*trnT* at the *ND4L* and *CytB* junction. 

As nearly all members of the subfamily Delphacinae in Delphacidae have both *ND6*-*trnP*-*trnT* and *trnC*-*trnW* rearrangements, they are synapomorphy for the subfamily, but the taxonomic expansion of these rearrangements to other subfamilies in Delphacidae should wait, as a mitogenome sequence in another subfamily, Asiracinae, at present, is available and contains only a single species (*Ugyops* sp.), which has A type arrangement. 

Previously, gene rearrangement and gene multiplication in a certain species within a genus have been exemplified in the honeybee genus, *Apis*, in Hymenoptera. For example, mitogenomes of eight species of *Apis* present two different arrangements; *trnE*-*trnS_1_* (E-S_1_ type) and *trnS_1_*-*trnE* (S_1_-E type) at the A+T-rich region and *ND2* junction [96], indicating that gene rearrangement occurs in a certain species within a genus. Of species with the S_1_-E type, only *A. koschevnikovi* has tandemly triplicated copies of *trnM* at the downstream of *trnE*, and only *A. andreniformis* has five additional copies of *trnL_1_* at the downstream of *trnE*, along with a likely original copy of this tRNA located at the *ND1* and *lrRNA* junction, the position of which is ancestral in insects [96]. Considering these examples, gene duplication and translocation could possibly only occur independently in a species after the divergence of the particular species from its ancestor. 

On the other hand, the intraspecies variation of gene arrangement might be very rare and unconventional, although fair judgment for its extensiveness might limitedly be possible due to the limited availability of multi-individual sequences in a species. Furthermore, gene rearrangement data, independent of nucleotide sequences, have been signified to have phylogenetic signals for the inference of evolutionary relationships in higher levels of taxonomic groups, rather than at individual levels [97,98,99]. Nevertheless, it might be noteworthy that Delphacinae is the only subfamily that has evolved a different gene arrangement (B, B’, and C types) compared to other families and subfamilies in Fulgoroidea. Therefore, members of the subfamily may, under slightly different evolutionary forces, be prone to evolve gene rearrangement and the incorporation of additional copies of the tRNAs in the genome. As more studies focusing on these aspects become available, further close inference of gene arrangement for delphacid planthoppers may be possible. 

### 3.8. Phylogenetic Relationships

**Figure 10:**
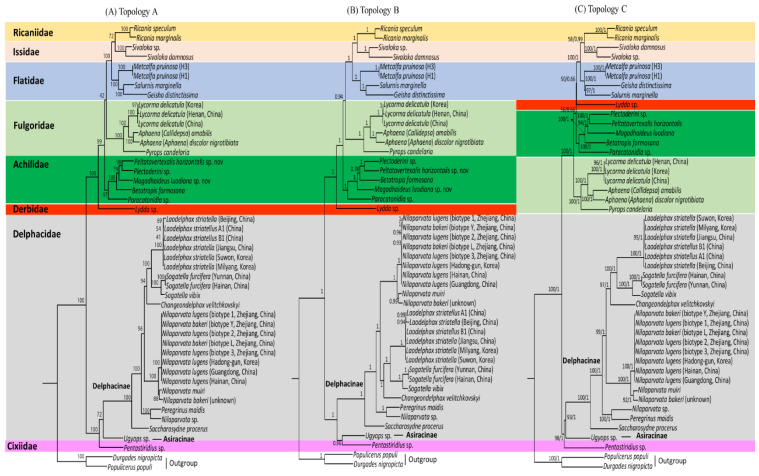
Phylogeny of Fulgoroidea using amino acids (AAs) and nucleotide (NU) sequences of 11 concatenated protein-coding genes. The numbers at each node specify bootstrap percentages of 1,000 pseudoreplicates for maximum-likelihood (ML) analysis and Bayesian posterior probabilities for Bayesian inference (BI) analysis. The scale bar indicates the number of substitutions per site. (**A**) Topology A by AA-ML analysis. (**B**) Topology B by AA-BI analysis. (**C**) Topology C by NU-ML and NU-BI analyses. *Durgades nigropicta* and *Populicerus populi* from another infraorder, Cicadomorpha in Auchenorrhyncha, were used as outgroups.

Four phylogenetic analyses were performed in combination with different datasets (AA for amino acid sequences and NU for nucleotide sequences) and algorithms (ML and BI methods). The four analyses (AA-ML, AA-BI, NU-ML, and NU-BI) resulted in three slightly, but importantly different, topologies, providing two AA-based trees (topology A by AA-ML dataset and topology B by AA-BI dataset) and only one NU-based tree (topology C both by NU-ML and NU-BI datasets; Figure 10). All phylogenetic analyses divided the eight fulgoroid families into two clades: one consisted of Ricaniidae, Issidae, Flatidae, Fulgoridae, Achilidae, and Derbidae, and the other consisted of Delphacidae and Cixiidae, each of which was supported relatively strongly in all analyses (BS = 98–100%, BPP = 1; Figure 10). The three topologies differ by the changes in positions and relationships among Fulgoridae, Achilidae, and Derbidae, with regard to consistent relationships among Ricaniidae, Issidae, and Flatidae (RIF; between topologies A, B vs. topology C) and the fluctuation in the monophyly of Delphacidae with regard to Cixiidae (between topology A vs. topologies B, C), whereas monophyly of Fulgoroidea was always supported with the highest nodal supports (BS = 100%, BPP = 1) (Figure 10).

Among the eight families included in the analyses, six were represented by more than two taxa and five of them, excluding Delphacidae, were supported as the monophyletic groups, almost always with the highest nodal supports (BS = 97–100%, BPP = 1.0) (Table S11; Figure 10). In particular, *M. pruinosa* and *S. marginella* belonging to Flatidae formed a strong monophyletic group with the co-familial species *G. distinctissima* in all analyses (BS = 100%, BPP = 1.0). However, the most specious Delphacidae, represented by two subfamilies (Asiracinae and Delphacinae), formed a non-monophyletic group in the AA-BI analysis by clustering the Asiracinae and Cixiidae, each of which was represented by a single species (topology B; Figure 10B), whereas monophyly of Delphacidae was supported in the AA-ML, NU-ML, and NU-BI analyses (topologies A and C) with moderate to high nodal supports (BS = 72, 93%, BPP = 1; Figure 10A,C), presenting an equivocal relationship with regard to the monophyly of Delphacidae. A previous study based on DNA nucleotide sequence data from three nuclear (nr) genes, mt *COI*, and 132 morphological characters also showed equivocal relationships, forming each member of Delphacidae and Cixiidae, unusual sister groups in a maximum parsimony (MP) analysis [100]. Further, other molecular and morphological studies have not yet reached a full conclusion with regard to their monophyly [29,101]. 

Nevertheless, several studies have shown mitogenome arrangement, independent of gene sequences, as an important evolutionary characteristic, which is used to infer the relationships between organismal phylogeny and rearrangement evolution in a diverse array of taxonomic groups [64,97,98,99,102,103,104,105,106]. If the gene rearrangement data are implemented to the current phylogeny, the clustering of Asiracinae in Delphacidae and Cixiidae, both of which evidenced an ancestral arrangement (type A), excluding rearranged subfamily Delphacinae (B, B’, and C types), may truly signal phylogenetic closeness between Asiracinae and Cixiidae, resulting in non-monophyly of Delphacidae (Figure 10B), an expanded sampling will likely result in better inference of their relationships. 

With regard to familial relationships, RIF as one clade with the highest nodal supports of all analyses formed Ricaniidae and Issidae as the sister group with moderate (BS = 58–72%) to higher nodal supports (BPP = 0.99–1.0) (Table S11; Figure 10). Although nodal support by ML analyses was comparatively lower than that of BI, this trend has previously been recognized [107]. 

Previously, several types of data have supported RIF as an inclusive group: morphology (Figure 1A–D), concatenated gene segments (Figure 1F,G), and mitogenome sequences (Figure 1J–P). Among them, the sister relationship between Ricaniidae and Issidae, leaving Flatidae as the sister lineage of the two families, was supported only in the studies that used several concatenated gene segments and mitogenome sequences (Figure 1F–N) but not those from morphological data (Figure 1A–E). For example, Urban and Cryan (2007) performed phylogenetic analyses using DNA nucleotide sequences from seven genes (*18S rDNA*, *28S rDNA*, *histone H3*, *histone 2A, wingless*, *COI,* and *ND4*) and 86 in-group exemplars representing all major lineages of Hemiptera. They showed the sister relationship between Ricaniidae and Issidae with higher nodal support by ML analysis (BS = 91%) and placed Flatidae as the sister to the Ricaniidae + Issidae group, with the higher nodal support (BS = 87%) (Figure 1G). Furthermore, several mitogenome-based studies, using 13 PCGs by the Neighbor-Joining method, ML method alone, and both ML and BI methods, all strongly supported the sister relationships between Ricaniidae and Issidae, leaving Flatidae as the basal lineage of the two families (Figure 1J–N), but exceptions also exist (Figure 1O,P). 

On the other hand, the morphology-based analysis presented either unresolved relationships (Figure 1A,B) or alternative relationships among RIF, other than the sister relationships between Ricaniidae and Issidae (Figure 1C–E). In particular, the sister relationship between Ricaniidae and Flatidae is supported by the loss of posterior tentorial arms in the female genitalia (Figure 1D) [27] and a piercin–excavating ovipositor, which inserts the eggs into the plant [25]. Support for the sister relationship between Flatidae and Ricaniidae is also found in the concatenated gene-based analysis of the Fulgoroidea (66 species in 23 families) using nr (*18S rRNA* and *28S rRNA*) and mt (*16S rRNA* and *CytB*) DNA sequences [30] (Figure 1H,I). They support the sister relationship between Ricaniidae and Flatidae with the higher nodal supports in ML and BI trees (BS = 95%, BPP = 1), whereas the sister group to the Ricaniidae + Flatidae group was unequivocal by the placement of moderately supported Achilidae in ML analysis (Figure 1H) or by the unresolved relationships among the Issidae + Achilidae + Fulgoridae group in the BI tree (Figure 1I). Therefore, whereas the relationships among RIF were equivocal among previous studies, the current study strongly supports a ((R + I) + F) topology. 

In contrast to consistent relationships among RIF in our analyses, the relationships of the other families fluctuate, providing two different topologies (topologies A and B vs. topology C; Figure 10). In topologies A and B, the sister relationships between RIF and Fulgoridae, with the placement of Achilidae as an early derived lineage and Derbidae as the most basal lineage in non-Delphacidae and Cixiidae clades, were supported, presenting familial relationships of (((RIF + Fulgoridae) + Achilidae) + Derbidae) (Figure 10A,B). On the other hand, topology C supported Derbidae as the sister to the RIF group, instead of Fulgoridae, presenting familial relationships of (((RIF + Derbidae) + Achilidae) + Fulgoridae) (Figure 10C). Nodal support for the sister relationships between RIF and Fulgoridae in topologies A and B was either lower (BS = 42%; Table S11) or higher (BPP = 0.94), but the sister relationships between RIF and Derbidae in topologies C were always lower (BS = 50%, BPP = 0.66). Taken together, the sister group to RIF is not consistent among datasets and algorithms, although the RIF + Fulgoridae group was slightly better supported. In contrast to the fluctuating relationships of the sister group to RIF, the placement of Achilidae as an earlier-derived lineage to the RIF + Fulgoridae group was strongly supported in AA-ML and AA-BI analyses (Figure 10A,B; BS = 99%, BPP = 1), whereas the placement of Achilidae as an earlier-derived lineage to the RIF + Derbidae group was poorly supported both in NU-ML and NU-BI analyses (Figure 10C; BS = 56%, BPP = 0.66). 

Within the non-Delphacidae and Cixiidae clade, the placement of Achilidae as an early derived lineage to the RIF + Fulgoridae group has been supported using morphological data (Figure 1C) and multiple genes by the MP method (Figure 1F), as was supported in topologies A and B (Figure 10A,B). However, this result sharply contrasts with those of other studies, such as those that support the sister relationships between Achilidae and Derbidae using morphology (Figure 1D) and unresolved relationships among the RIF + Achilidae + Fulgoridae + Derbidae group (Figure 1B) and multiple genes by the BI method (Figure 1G). In particular, recent two mitogenome-based analyses, which include Achilidae but not Derbidae, both placed the Achilidae and Fulgoridae as the sister group and placed this group as the sister to the RIF group (Figure 10I,P). It seems that newly added available mitogenome sequences in this study, subsequent to previous studies [21,22], affect the position and relationships of Achilidae and Fulgoridae, although this study is also limited by taxon diversity.

Delphacidae and Cixiidae, each as independent groups (topologies A and C) or a sister group between Cixiidae and Asiracinae, excluding another subfamily, Delphacinae in Delphacidae (topology B), were placed as the most early derived lineage in fulgoroid families (Figure 10). Nodal supports for the placement of the two families as the most basal lineages were strongly supported in all analyses (BS = 100%, BPP = 1; Figure 10). A close relationship between Cixiidae and Delphacidae has previously been identified. Asche (1987) showed that the two families share an orthopteroid ovipositor and fused gonapophyses on abdominal segment 9. Additionally, the placement of the two families as the most early derived lineage to the remaining fulgoroid families was supported by several types of data, such as morphological data (Figure 1D), concatenated gene sequence data (Figure 1F–I), and mitogenome-based studies (Figure 1K–N), whereas the placement of Delphacidae alone as an earlier-derived lineage than Cixiidae has been suggested based only on adult and larval morphology (Figure 1C). However, no previous studies support the placement of Cixiidae alone as the most basal lineage of fulgoroid families, whereas our topologies A and C did (Figure 10A,C). 

In summary, our phylogenetic analysis using mitogenome sequences consistently supported the relationships of ((R + I) + F), but the early derived lineages to the RIF group were supported either as (((RIF + Fulgoridae) + Achilidae) + Derbidae) by AA-based analysis or (((RIF + Derbidae) + Achilidae) + Fulgoridae) by NU-based analyses. Overall, slightly higher supports for the former topology suggest that AA sequences, which recorded NU data into AA data, better support the phylogenetic positions of the Fulgoridae, Achilidae, and Derbidae, although limited taxon diversity may also be responsible for such incongruence. The classification of Delphacidae as a non-monophyletic group is unconventional, but gene rearrangement data supported such a possibility by having both ancestral and derived arrangements in the members of Delphacidae.

## Figures and Tables

**Table 1:** Characteristics of 45 mitochondrial genomes of Fulgoroidea.

Taxon	Size (bp)	A/T Content (%)	PCG		srRNA		lrRNA		tRNA		A+T-Rich Region		GenBank Accession No.	References
			No. Codons ^a^	AT (%)	Size (bp)	AT (%)	Size (bp)	AT (%)	Size (bp)	AT (%)	Size (bp)	AT (%)		
Fulgoroidea														
Flatidae														
Flatinae														
*Metcalfa pruinosa* (H1)	16,312	76.62	3,656	75.78	717	77.96	1,226	79.53	1,445	76.82	1,788	77.46	MK303326	This study
*Metcalfa pruinosa* (H3)	16,314	76.62	3,656	75.79	717	77.96	1,226	79.53	1,445	76.82	1,790	77.54	MN417319	This study
*Salu* *rnis marginella*	16,126	75.73	3,637	75.33	724	76.52	1,204	78.90	1,390	76.12	1,836	74.73	MT628542	This study
*Geisha distinctissima* ^‡^	15,971	75.11	3,633	73.47	729	77.64	1,198	79.55	1,408	76.99	1,702	79.49	FJ230961	Song and Liang [5]
Fulgoridae														
Fulgorinae														
*Pyrops candelaria*	16,021	74.36	3,653	72.64	717	76.71	1,214	74.96	1,440	74.10	1,592	83.73	FJ006724	Song et al. [7]
Aphaeninae														
*Lycorma delicatula* (Henan, China)	15,946	76.36	3,641	75.04	721	77.67	1,214	77.35	1,423	76.11	1,642	83.74	EU909203	Song et al. [7]
*Lycorma delicatula* (China)	15,410	76.29	3,644	75.27	792	77.40	1,212	77.97	1,409	76.37	1,043	83.32	FJ456942	Hua et al. [3]
*Lycorma delicatula* (Korea)	15,798	76.50	3,646	75.30	735	77.28	1,209	77.67	1,410	76.45	1,495	83.48	MN607209	Jeong et al. [23]
*Aphaena* (Callidepsa) *amabilis*	16,237	77.90	3,657	77.16	730	78.77	1,210	77.93	1,403	76.48	1,869	82.24	MN025522	Wang et al. [21]
*Aphaena* (Aphaena) *discolor nigrotibiata*	16,116	77.02	3,654	76.12	733	78.72	1,210	78.18	1,397	75.88	1,764	81.07	MN025523	Wang et al. [21]
Achilidae														
Achilinae														
*Betatropis formosana*	16,161	77.77	3,661	76.25	726	78.37	1,182	79.53	1,397	77.24	1,778	84.81	MH324927	Xu et al. [22]
*Magadhaideus luodiana* sp. nov	15,885	74.35	3,650	72.48	726	74.38	1,213	78.57	1,406	76.17	1,550	82.00	MH324928	Xu et al. [22]
*Peltatavertexalis horizontalis* sp. nov	15,787	75.53	3,638	74.16	729	76.13	1,213	78.15	1,400	76.00	1,459	81.91	MH324929	Xu et al. [22]
*Plectoderini* sp.	16,216	75.70	3,645	74.21	729	75.72	1,199	79.32	1,417	75.72	1,850	81.30	MH324930	Xu et al. [22]
*Paracatonidia* sp.	15,214	76.48	3,627	75.30	726	77.13	1,198	78.21	1,412	77.41	909	84.60	MH324931	Xu et al. [22]
Cixiidae														
Cixiinae														
*Pentastiridius* sp. ^‡^	12,231	76.97	3,255	75.64	-	-	-	-	1,296	77.78	-	-	KY039133	Song et al. [15]
Derbidae														
Otiocerinae														
*Lydda* sp.^‡^	14,755	77.93	3,599	77.45	724	79.56	1,201	81.85	1,335	79.10	565	69.38	KY039126	Song et al. [15]
Issidae														
Issinae														
*Sivaloka damnosus*	15,287	76.52	3,625	75.56	711	76.79	1,192	78.44	1,424	77.18	1,994	81.69	FJ360694	Song et al. [6]
*Sivaloka* sp.^‡^	15,034	75.83	3,569	74.82	800	75.12	1,210	79.83	1,321	77.52	755	76.16	KY039137	Song et al. [15]
Ricaniidae														
Ricaniinae														
*Ricania marginalis*	15,698	76.12	3,646	75.00	736	75.68	1,216	78.70	1,426	75.67	1,324	82.33	JN242415	Song et al. [7]
*Ricania speculum*	15,729	75.75	3,640	74.67	727	75.79	1,198	78.30	1,415	75.76	1,346	80.76	KX371891	Zhang et al. [11]
Delphacidae														
Asiracinae														
*Ugyops* sp.	15,259	77.65	3,612	76.41	767	74.97	1,206	80.76	1,397	77.31	1,031	88.85	MH352481	Yu and Liang [16]
Delphacinae														
Saccharosydnini														
*Saccharosydne procerus*	16,031	80.53	3,602	79.16	754	78.12	1,217	83.48	1,404	79.91	1,662	88.03	MG515237	Huang and Qin [14]
Delphacini														
*Changeondelphax velitchkovskyi*	16,449	75.72	3,607	74.48	752	74.60	1,213	77.91	1,386	76.41	1,781	80.12	MG049916	Huang and Qin [13]
*Nilaparvata bakeri* (unknown)	14,394	77.07	3,582 ^†^	76.58	754	73.87	1,215	79.01	1,393	78.89	-	-	KC333655	Lv et al. [10]
*Nilaparvata lugens* (biotype Y, Zhejiang, China)	14,365	76.67	3,581	75.92	751	74.83	1,219	79.57	1,390	78.71	-	-	KC333653	Lv et al. [10]
*Nilaparvata lugens* (biotype L, Zhejiang, China)	14,366	76.72	3,581	75.98	751	74.83	1,219	79.49	1,392	78.74	-	-	KC333654	Lv et al. [10]
*Nilaparvata lugens* (Hainan, China)	17,619	76.95	3,608	76.01	748	75.00	1,219	79.57	1,409	78.57	2,429	79.29	JX880069	Zhang et al. [8]
*Nilaparvata lugens* (Hadong-gun, Korea) ^‡^	17,610	77.12	3,619	76.19	748	75.00	1,219	79.49	1,534	78.36	2,424	79.70	MK590088	Park et al. [19]
*Nilaparvata lugens* (Guangdong, China)	17,606	77.09	3,607	76.10	748	75.00	1,219	79.49	1,535	78.31	2,424	79.79	MK606371	Choi et al. [17]
*Nilaparvata lugens* (biotype 1, Zhejiang, China)	14,364	76.69	3,582	75.95	751	74.83	1,219	79.57	1,390	78.71	-	-	JN563995	Lv et al. [10]
*Nilaparvata lugens* (biotype 2, Zhejiang, China)	14,367	76.68	3,582	75.94	751	74.83	1,219	79.57	1,392	78.74	-	-	JN563996	Lv et al. [10]
*Nilaparvata lugens* (biotype 3, Zhejiang, China)	14,367	76.67	3,582	75.91	751	74.83	1,219	79.66	1,392	78.74	-	-	JN563997	Lv et al. [10]
*Nilaparvata muiri*	14,371	76.15	3,582 ^†^	75.41	753	74.24	1,219	78.42	1,391	78.86	-	-	JN563998	Lv et al. [10]
*Nilaparvata* sp.^‡^	15,274	76.24	3,609	75.61	749	74.63	1,212	79.21	1,396	78.51	725	73.10	KY039125	Song et al. [15]
*Peregrinus maidis*	16,279	77.75	3,607	75.74	750	76.13	1,222	79.87	1,390	78.85	1,596	86.15	MG049917	Huang and Qin [12]
*Sogatella furcifera* (Hainan, China)	16,612	76.19	3,606	74.44	747	74.03	1,224	77.94	1,389	77.75	2,223	82.50	KC512914	Zhang et al. [9]
*Sogatella furcifera* (Yunnan, China)	16,654	76.19	3,609	74.44	747	74.16	1,225	77.96	1,388	77.74	2,264	82.29	KC512915	Zhang et al. [9]
*Sogatella vibix*	16,554	76.04	3,609	75.39	749	74.77	1,227	79.14	1,395	77.92	2,167	75.91	MG515238	Huang and Qin [14]
*Laodelphax striatellus* (Jiangsu, China)	16,431	77.17	3,613	75.74	747	74.83	1,219	78.59	1,406	77.95	2,042	83.20	JX880068	Zhang et al. [8]
*Laodelphax striatellus* (Suwon, Korea)	16,359	77.26	3,609	75.81	747	74.83	1,219	78.67	1,406	78.09	1,972	83.67	MK838101	Park et al. [18]
*Laodelphax striatellus* (Milyang, Korea)	16,359	77.27	3,609	75.81	747	74.83	1,219	78.67	1,406	78.09	1,972	83.72	MK862265	Seo et al. [20]
*Laodelphax striatellus* A1 (China)	16,428	77.18	3,613	75.75	747	74.83	1,219	78.67	1,406	78.09	2,039	83.13	MK292897	Sun et al. [2]
*Laodelphax striatellus* B1 (China)	16,418	77.18	3,613	75.77	747	74.83	1,219	78.67	1,409	78.07	2,032	83.12	MK292932	Sun et al. [2]
*Laodelphax striatellus* (Beijing, China)	16,513	77.19	3,676	75.96	747	74.56	1,219	78.59	1,417	78.19	2,040	83.09	FJ360695	Song and Liang [4]

^†^ The *ND3* gene of both *Nilaparvata muiri* and *N. bakeri* has an unusually short length in comparison to other species. ^‡^ Indicates partially or fully re-annotated in this study.

**Table 2:** Summaries of mitochondrial genomes of *Metcalfa pruinosa* (H1 and H3 haplotypes) and *Salurnis marginella*.

Gene	Strand	Anticodon	Start Codon	Stop Codon	*M. pruinosa* (H1)	*M. pruinosa* (H3)	*Salurnis* *marginella*
*trnI*	+	GAT			1–65 (65)	1–65 (65)	1–62 (62)
*trnQ*	−	TTG			69–137 (69)	69–137 (69)	81–149 (69)
*trnM*	+	CAT			137–202 (66)	137–202 (66)	149–214 (66)
*ND2*	+		ATT	TAA	203–1180 (978)	203–1180 (978)	215-1180 (966)
*trnW*	+	TCA			1179–1246 (68)	1179–1246 (68)	1179–1243 (65)
*trnC*	−	GCA			1239–1302 (64)	1239–1302 (64)	1236–1295 (60)
*trnY*	−	GTA			1310–1373 (64)	1310–1373 (64)	1296–1356 (61)
*COI*	+		ATC ^a^/ATG ^b^	TAA	1374–2918 (1545) ^a^	1374–2918 (1545) ^a^	1362–2897 (1536) ^b^
*trnL_2_*	+	TAA			2921–2983 (63)	2921–2983 (63)	2898–2959 (62)
*COII*	+		ATA	TAA	2984–3661 (678)	2984–3661 (678)	2960–3640 (681)
*trnK*	+	CTT			3672–3741 (70)	3672–3741 (70)	3641–3709 (69)
*trnD*	+	GTC			3742–3810 (69)	3742–3810 (69)	3710–3772 (63)
*ATP8*	+		ATT	TAA	3811–3966 (156)	3811–3966 (156)	3773–3925 (153)
*ATP6*	+		ATG	T	3960–4611 (652)	3960–4611 (652)	3919–4570 (652)
*COIII*	+		ATG	TAA	4612–5394 (783)	4612–5394 (783)	4571–5353 (783)
*trnG*	+	TCC			5401–5465 (65)	5401–5465 (65)	5355–5415 (61)
*ND3*	+		ATG ^a^/ATT ^b^	TAA	5467–5817 (351) ^a^	5467–5817 (351) ^a^	5451–5786 (336) ^b^
*trnA*	+	TGC			5816–5883 (68)	5816–5883 (68)	5786–5848 (63)
*trnR*	+	TCG			5901–5964 (64)	5901–5964 (64)	5849–5909 (61)
*trnN*	+	GTT			5990–6054 (65)	5990–6054 (65)	5911–5973 (63)
*trnS_1_*	+	GCT			6056–6116 (61)	6056–6116 (61)	5972–6031 (60)
*trnE*	+	TTC			6120–6184 (65)	6120–6184 (65)	6034–6095 (62)
*trnF*	−	GAA			6197–6261 (65)	6197–6261 (65)	6094–6156 (63)
*ND5*	−		ATG ^a^/GTG ^b^	TAA	6268–7950 (1683) ^a^	6268–7950 (1683) ^a^	6156–7835 (1680) ^b^
*trnH*	−	GTG			7952–8016 (65)	7952–8016 (65)	7836–7897 (62)
*ND4*	−		ATG	TAG ^1^/T ^2^	8031–9362 (1332) ^1^	8031–9362 (1332) ^1^	7898–9221 (1324) ^2^
*ND4L*	−		ATG	TAA	9362–9634 (273)	9362–9634 (273)	9223–9495 (273)
*trnT*	+	TGT			9637–9700 (64)	9637–9700 (64)	9498–9558 (61)
*trnP*	−	TGG			9708–9773 (66)	9708–9773 (66)	9560–9622 (63)
*ND6*	+		ATA	TAA	9775–10275 (501)	9775–10275 (501)	9624–10121 (498)
*CytB*	+		ATG	TAA	10280–11404 (1125)	10280–11404 (1125)	10114–11235 (1122)
*trnS_2_*	+	TGA			11420–11482 (63)	11420–11482 (63)	11235–11295 (61)
*ND1*	−		ATG	TAA	11492–12442 (951)	11492–12442 (951)	11289–12230 (942)
*trnL*_1_	−	TAG			12444–12508 (65)	12444–12508 (65)	12232–12293 (62)
*lrRNA*	−				12509–13734 (1226)	12509–13734 (1226)	12294–13497 (1205)
*trnV*	−	TAC			13735–13807 (73)	13735–13807 (73)	13498–13567 (70)
*srRNA*	−				13808–14524 (717)	13808–14524 (717)	13567–14290 (724)
A+T–rich region					14525–16312 (1788)	14525–16314 (1790)	14291–16126 (1836)

Note: The gene abbreviations are as follows: *COI*, *COII*, and *COIII* refer to the cytochrome oxidase subunits; *CytB* refers to cytochrome b; *ND1-6* refers to NADH dehydrogenase components; *srRNA* and *lrRNA* refer to small and large subunit ribosomal RNA (rRNA) genes, respectively. tRNAs are denoted as one-letter symbols in accordance with the IUPAC-IUB single-letter amino acid codes, except those encoding leucine and serine, which are labeled *trnL*_1_ for the CTN codon family, *trnL_2_* for the TTR codon family, *trnS*_1_ for the AGN codon family, and *trnS_2_* for the TCN codon family. Superscripts indicate identical start and stop codons among skipper species. +, genes encoded in major strand; −, genes encoded in minor strand. Values in parentheses indicate gene size (bp).

## Data Availability

The data presented in this study are available in the article and the Supplementary Material.

## Supplementary Materials

The following are available online at https://www.mdpi.com/article/10.3390/cimb43030099/s1, Table S1: List of primers used to amplify the gapped regions of *Metcalfa pruinosa* H1 and H3 haplotypes; Table S2: Primers used to amplify and sequence the mitochondrial genome of *Salurnis marginella*; Table S3: Summary of mitochondrial genomes of Fulgoroidea; Table S4: Frequency of four most frequently used codons in 45 mitochondrial genomes of Fulgoroidea; Table S5: Size of mitochondrial tRNAs available in Fulgoroidea; Table S6: Skewness of 45 mitochondrial genomes Fulgoroidea; Table S7: Ka/Ks ratio of protein-coding genes in 45 mitochondrial genomes of Fulgoroidea; Table S8: ANOVA test for mean Ks/Ks ratio of 45 mitochondrial genomes of Fulgoroidea; Table S9: Overlapping and intergenic-spacer sequences of Fulgoroidea mitochondrial genomes; Table S10: BLAST results of the repeat sequences found in the A+T-rich region of Flatidae species; Table S11: Node supports for monophyletic families and inter-familial relationships within Fulgoroidea.

## References

[1] Du Z., Wu Y., Chen Z., Cao L., Ishikawa T., Kamitani S., Sota T., Song F., Tian L., Cai W. (2020). Global phylogeography and invasion history of the spotted lanternfly revealed by mitochondrial phylogenomics. Evol. Appl..

[2] Sun J.T., Duan X.Z., Hoffmann A.A., Liu Y., Garvin M.R., Chen L., Hu G., Zhou J.C., Huang H.J., Xue X.F. (2019). Mitochondrial variation in small brown planthoppers linked to multiple traits and probably reflecting a complex evolutionary trajectory. Mol. Ecol..

[3] Hua J.M., Li M., Dong P.Z., Cui Y., Xie Q., Bu W.J. (2009). Phylogenetic analysis of the true water bugs (Insecta: Hemiptera: Heteroptera: Nepomorpha): Evidence from mitochondrial genomes. BMC Evol. Biol..

[4] Song N., Liang A.-P. (2009). Complete Mitochondrial Genome of the Small Brown Planthopper, *Laodelphax striatellus* (Delphacidae: Hemiptera), with a Novel Gene Order. Zool. Sci..

[5] Song N., Liang A.P. (2009). The complete mitochondrial genome sequence of *Geisha distinctissima* (Hemiptera: Flatidae) and comparison with other hemipteran insects. Acta Biochim. Biophys. Sin..

[6] Song N., Liang A.-P., Ma C. (2010). The Complete Mitochondrial Genome Sequence of the Planthopper, *Sivaloka damnosus*. J. Insect Sci..

[7] Song N., Liang A.-P., Bu C.-P. (2012). A Molecular Phylogeny of Hemiptera Inferred from Mitochondrial Genome Sequences. PLoS ONE.

[8] Zhang K.-J., Zhu W.-C., Rong X., Zhang Y.-K., Ding X.-L., Liu J., Chen D.-S., Du Y., Hong X.-Y. (2013). The complete mitochondrial genomes of two rice planthoppers, *Nilaparvata lugens* and *Laodelphax striatellus*: Conserved genome rearrangement in Delphacidae and discovery of new characteristics of atp8 and tRNA genes. BMC Genom..

[9] Zhang K.-J., Zhu W.-C., Rong X., Liu J., Ding X.-L., Hong X.-Y. (2014). The complete mitochondrial genome sequence of *Sogatella furcifera* (Horváth) and a comparative mitogenomic analysis of three predominant rice planthoppers. Gene.

[10] Lv L., Peng X., Jing S., Liu B., Zhu L., He G. (2015). Intraspecific and Interspecific Variations in the Mitochondrial Genomes ofNilaparvata(Hemiptera: Delphacidae). J. Econ. Entomol..

[11] Zhang Q.-X., Guan D.-L., Niu Y., Sang L.-Q., Zhang X.-X., Xu S.-Q. (2016). Characterization of the complete mitochondrial genome of the Asian planthopper Ricania speculum (Hemiptera: Fulgoroidea: Ricannidae). Conserv. Genet. Resour..

[12] Huang Y.-X., Qin D.-Z. (2017). The complete mitochondrial genome sequence of the corn planthopper, Peregrinus maidis (Hemiptera: Fulgoroidea). Mitochondrial DNA Part B.

[13] Huang Y.-X., Qin D.-Z. (2018). Sequencing and analysis of the complete mitochondrial genome of Changeondelphax velitchkovskyi (Hemiptera: Fulgoroidea). Mitochondrial DNA Part B.

[14] Huang Y.X., Qin D.Z. (2008). First mitogenome for the tribe Saccharosydnini (Hemiptera: Delphacidae: Delphacinae) and the phylogeny of three predominant rice planthoppers. Eur. J. Entomol..

[15] Song N., Cai W., Li H. (2017). Deep-level phylogeny of Cicadomorpha inferred from mitochondrial genomes sequenced by NGS. Sci. Rep..

[16] Yu F., Liang A.-P. (2018). The Complete Mitochondrial Genome of Ugyops sp. (Hemiptera: Delphacidae). J. Insect Sci..

[17] Choi N.J., Lee B.C., Park J., Park J. (2019). The complete mitochondrial genome of *Nilaparvata lugens* (Stål, 1854) captured in China (Hemiptera: Delphacidae): Investigation of intraspecies variations between countries. Mitochondrial DNA Part B.

[18] Park J., Jung J.K., Koh Y.H., Park J., Seo B.Y. (2019). The complete mitochondrial genome of Laodelphax striatellus (Fallén, 1826) (Hemiptera: Delphacidae) collected in a mid-Western part of Korean peninsula. Mitochondrial DNA Part B.

[19] Park J., Kwon W., Park J., Kim H.J., Lee B.C., Kim Y., Choi N.J. (2019). The complete mitochondrial genome of *Nilaparvata lugens* (stål, 1854) captured in Korea (Hemiptera: Delphacidae). Mitochondrial DNA Part B.

[20] Seo B.Y., Jung J.K., Koh Y.H., Park J. (2019). The complete mitochondrial genome of Laodelphax striatellus (Fallén, 1826) (Hemiptera: Delphacidae) collected in a southern part of Korean peninsula. Mitochondrial DNA Part B.

[21] Wang W., Huang Y., Bartlett C., Zhou F., Meng R., Qin D. (2019). Characterization of the complete mitochondrial genomes of two species of the genus Aphaena Guérin-Méneville (Hemiptera: Fulgoridae) and its phylogenetic implications. Int. J. Biol. Macromol..

[22] Xu S.Y., Long J.K., Chen X.S. (2009). Comparative analysis of the complete mitochondrial genomes of five Achilidae species (Hemiptera: Fulgoroidea) and other Fulgoroidea reveals conserved mitochondrial genome organization. Peer J..

[23] Jeong N.R., Kim M.J., Lee W., Lee G.-S., Kim I. (2020). Complete mitochondrial genome of the spotted lanternfly, Lycorma delicatula White, 1845 (Hemiptera: Fulgoridae). Mitochondrial DNA Part B.

[24] Muir F. (1930). On the classification of the Fulgoroidea (Homoptera). Ann. Mag. Nat. Hist..

[25] Asche M. Preliminary thoughts on the phylogeny of Fulgoromorpha (Homoptera Auchenorrhyncha). Proceedings of the 6th Auchenorrhyncha Meeting.

[26] Emeljanov A.F. (1990). An attempt of construction of the phylogenetic tree of the planthoppers (Homoptera, Cicadina). Entomol. Obozr..

[27] Bourgoin T. (1993). Female genitalia in Fulgoromorpha (Insecta, Hemiptera): Morphological and phylogenetical data. Ann. Soc. Entomol. Fr..

[28] Chen S., Yang C.Y. (1995). The metatarsi of the Fulgoroidea (Homoptera: Auchenorrhyncha). Chin. J. Entomol..

[29] Urban J.M., Cryan J.R. (2007). Evolution of the planthoppers (Insecta: Hemiptera: Fulgoroidea). Mol. Phylogenetics Evol..

[30] Song N., Liang A.-P. (2013). A Preliminary Molecular Phylogeny of Planthoppers (Hemiptera: Fulgoroidea) Based on Nuclear and Mitochondrial DNA Sequences. PLoS ONE.

[31] Dean H.A., Bailey J.C. (1961). A flatid planthopper, *Metcalfa pruinose*. J. Econ. Entomol..

[32] Wilson S.W., Lucchi A. (2001). Distribution and ecology *of Metcalfa pruinosa* and associated planthoppers in North America (Homoptera: Fulgoroidea), Atti della Academia Nazionale Italiana di Entomologia. Rend. Anno.

[33] Zangheri S., Donadini P. (1980). Comparsa nel veneto di un omottero neartico: *Metcalfa pruinosa* Say (Homoptera, Flatidae). Redia.

[34] EPPO (2017). PQR Database, European and Mediterranean Plant Protection Organization, Paris, France. http://www.eppo.int/DATABASES/pqr/pqr.htmREF.

[35] Lee H.S., Wilson S.W. (2010). First report of the Nearctic flatid planthopper *Metcalfa pruinosa* (Say) in the Republic of Korea (Hemiptera: Fulgoroidea). Entomol. News.

[36] Peng L.F., Zhang Y.L., Wang Y.L. (2009). A taxonomic study of the genus *Salurnis* Stal from China (Hemiptera, Flatidae) with description of two new record species. Acta Zootaxonomica Sinica.

[37] Kim D.E., Kil J.H. (2013). A photographic guide to the alien insects and their host plants in Korea. Nat. Life Korea.

[38] Kim N.Y., Kim S.H., Kim D.E., Lee D.H., Ryu T.B., Choi D., Lee H., Kim H.M., Kim M.Y., Kim Y.C. (2016). Ecological Studies of Alien Species (III).

[39] Park C.-G., Min S., Lee G.-S., Kim S., Lee Y., Lee S., Hong K.-J., Wilson S.W., Akimoto S.-I., Lee W. (2016). Genetic Variability of the Invasive SpeciesMetcalfa pruinose (Hemiptera: Flatidae) in the Republic of Korea. J. Econ. Entomol..

[40] Andrews S. (2010). FastQC: A Quality Control Tool for High Throughput Sequence Data. http://www.bioinformatics.babraham.ac.uk?/projects/fastqc/.

[41] Jiang H., Lei R., Ding S.-W., Zhu S. (2014). Skewer: A fast and accurate adapter trimmer for next-generation sequencing paired-end reads. BMC Bioinform..

[42] Allam A., Kalnis P., Solovyev V. (2015). Karect: Accurate correction of substitution, insertion and deletion errors for next-generation sequencing data. Bioinformatics.

[43] Hahn C., Bachmann L., Chevreux B. (2013). Reconstructing mitochondrial genomes directly from genomic next-generation sequencing reads-a baiting and iterative mapping approach. Nucleic Acids Res..

[44] Langmead B., Salzberg S.L. (2012). Fast Gapped-Read Alignment with Bowtie 2. Nat. Methods.

[45] De Pristo M.A., Banks E., Poplin R., Garimella K.V., Maguire J.R., Hartl C., Philippakis A., Del Angel G., Rivas M.A., Hanna M. (2011). A framework for variation discovery and genotyping using next-generation DNA sequencing data. Nat. Genet..

[46] Bernt M., Donath A., Donath A., Externbrink F., Florentz C., Fritzsch G., Pütz J., Middendorf M., Stadler P.F. (2013). MITOS: Improved de novo metazoan mitochondrial genome annotation. Mol. Phylogenet. Evol..

[47] Tamura K., Stecher G., Peterson D., Filipski A., Kumar S. (2013). MEGA6: Molecular evolutionary genetics analysis ver. 6.0. Mol. Biol. Evol..

[48] Swofford D.L. (2002). PAUP* Phylogenetic Analysis Using Parsimony (*and Other Method) Version 4. 10.

[49] Perna N.T., Kocher T.D. (1995). Patterns of nucleotide composition at fourfold degenerate sites of animal mitochondrial genomes. J. Mol. Evol..

[50] Posada D., Buckley T.R. (2004). Model Selection and Model Averaging in Phylogenetics: Advantages of Akaike Information Criterion and Bayesian Approaches over Likelihood Ratio Tests. Syst. Biol..

[51] Zhang Z., Li J., Yu J. (2006). Computing Ka and Ks with a consideration of unequal transitional substitutions. BMC Evol. Biol..

[52] Shao R., Campbell N.J.H., Schmidt E.R., Barker S.C. (2001). Increased rate of gene rearrangement in the mitochondrial genomes of three orders of hemipteroid insects. Mol. Biol. Evol..

[53] Wernersson R., Pedersen A.G. (2003). RevTrans—Constructing alignments of coding DNA from aligned amino acid sequences. Nucleic Acids Res..

[54] Castresana J. (2000). Selection of conserved blocks from multiple alignments for their use in phylogenetic tool. Mol. Biol. Evol..

[55] Lanfear R., Calcott B., Ho S.Y.W., Guindon S. (2012). PartitionFinder: Combined Selection of Partitioning Schemes and Substitution Models for Phylogenetic Analyses. Mol. Biol. Evol..

[56] Lanfear R., Calcott B., Kainer D., Mayer C., Stamatakis A. (2014). Selecting optimal partitioning schemes for phylogenomic datasets. BMC Evol. Biol..

[57] Lanfear R., Frandsen P., Wright A.M., Senfeld T., Calcott B. (2016). PartitionFinder 2: New Methods for Selecting Partitioned Models of Evolution for Molecular and Morphological Phylogenetic Analyses. Mol. Biol. Evol..

[58] Stamatakis A. (2014). RAxML version 8: A tool for phylogenetic analysis and post-analysis of large phylogenies. Bioinformatics.

[59] Ronquist F., Teslenko M., Van Der Mark P., Ayres D.L., Darling A., Hoehna S., Larget B., Liu L., Suchard M.A., Huelsenbeck J.P. (2012). MrBayes 3.2: Efficient Bayesian Phylogenetic Inference and Model Choice Across a Large Model Space. Syst. Biol..

[60] Miller M.A., Pfeiffer W., Schwartz T. (2010). Creating the CIPRES science gateway for inference of large phylogenetic trees. Proceedings of the 9th Gateway Computing Environments Workshop (GCE).

[61] Wu Y., Dai R., Zhan H., Qu L. (2015). Complete mitochondrial genome of Drabescoides nuchalis (Hemiptera: Cicadellidae). Mitochondrial DNA Part A.

[62] Wang J.J., Yang M.F., Dai R.H., Li H., Wang X.Y. (2018). Characterization and phylogenetic implications of the complete mitochondrial genome of Idiocerinae (Hemiptera: Cicadellidae). Int. J. Biol. Macromol..

[63] Letunic I., Bork P. (2019). Interactive Tree of Life (iTOL) v4: Recent updates and new developments. Nucleic Acids Res..

[64] Boore J.L. (1999). Animal mitochondrial genomes. Nucleic Acids Res..

[65] Kim J.S., Kim M.J., Jeong J.S., Kim I. (2018). Complete mitochondrial genome of Saturnia jonasii (Lepidoptera: Saturniidae): Genomic comparisons and phylogenetic inference among Bombycoidea. Genomics.

[66] Shi X., Zhu Q., Wu N., Tu J., Yang D., Xu H., Yao Y., Yang M., Li D. (2015). The complete nucleotide sequence of the mitochondrial genome of Drosophila formosana (Diptera: Drosophilidae). Mitochondrial DNA Part A.

[67] Wolstenholme D.R. (1992). Animal Mitochondrial DNA: Structure and Evolution. Int. Rev. Cytol..

[68] Du Y., Zhang C., Dietrich C.H., Zhang Y., Dai W. (2017). Characterization of the complete mitochondrial genomes of Maiestas dorsalis and Japananus hyalinus (Hemiptera: Cicadellidae) and comparison with other Membracoidea. Sci. Rep..

[69] Chen J., Wang Y., Qin M., Jiang L.-Y., Qiao G.-X. (2018). The mitochondrial genome of Greenidea psidii van der Goot (Hemiptera: Aphididae: Greenideinae) and comparisons with other Aphididae aphids. Int. J. Biol. Macromol..

[70] Hebert P.D.N., Cywinska A., Ball S.L., Dewaard J.R. (2003). Biological identifications through DNA barcodes. Proc. R. Soc. B: Boil. Sci..

[71] Hajibabaei M., Singer G.A., Hebert P.D., Hickey D.A. (2007). DNA barcoding: How it complements taxonomy, molecular phylogenetics and population genetics. Trends Genet..

[72] Lee J.Y., Wang A.R., Choi Y.S., Thapa R., Kwon H.W., Kim I. (2016). Mitochondrial DNA variations in Korean *Apis cerana* (Hymenoptera: Apidae) and development of another potential marker. Apidologie.

[73] Choi D.-S., Park J.S., Kim M.J., Kim J.S., Jeong S.Y., Jeong J.S., Park J., Kim I. (2017). Geographic variation in the spotted-wing drosophila, Drosophila suzukii (Diptera: Drosophilidae), based on mitochondrial DNA sequences. Mitochondrial DNA Part A.

[74] Sanaei E., Husemann M., Seiedy M., Rethwisch M., Tuda M., Toshova T.B., Kim M.J., Atanasova D., Kim I. (2019). Global genetic diversity, lineage distribution, and Wolbachia infection of the alfalfa weevil Hypera postica (Coleoptera: Curculionidae). Ecol. Evol..

[75] Kim M.J., Cho Y., Wang A.R., Kim S.-S., Choi S.-W., Kim I. (2020). Population genetic characterization of the black-veined white, Aporia crataegi (Lepidoptera: Pieridae), using novel microsatellite markers and mitochondrial DNA gene sequences. Conserv. Genet..

[76] Wang A.-R., Kim M.-J., Cho Y.-B., Wan X., Kim I.-S. (2011). Geographic Genetic Contour of a Ground Beetle, Scarites aterrimus (Coleoptera: Carabidae) on the Basis of Mitochondrial DNA Sequence. Int. J. Ind. Entomol..

[77] Arca M., Mougel F., Guillemaud T., Dupas S., Rome Q., Perrard A., Muller F., Fossoud A., Capdevielle-Dulac C., Torres-Leguizamon M. (2015). Reconstructing the invasion and the demographic history of the yellow-legged hornet, Vespa velutina, in Europe. Biol. Invasions.

[78] Kwon D.H., Kim M., Kim H., Lee Y., Hong K.-J., Lee S.H., Lee S. (2015). Estimation of genetic divergence based on mitochondrial DNA variation for an invasive alien species, *Metcalfa pruinosa* (Say), in Korea. J. Asia-Pacific Entomol..

[79] Takahashi R., Okutama H., Kiyoshi T., Takahashi J. (2017). Complete mitochondrial DNA sequence of the invasive hornet *Vespa velutina* (Insecta, Hymenoptera) found in Japan. Mitochondrial DNA Part B.

[80] Kim K.-G., Hong M.Y., Kim M.J., Im H.H., Kim M.I., Bae C.H., Seo S.J., Lee S.H., Kim I. (2009). Complete mitochondrial genome sequence of the yellow-spotted long-horned beetle Psacothea hilaris (Coleoptera: Cerambycidae) and phylogenetic analysis among coleopteran insects. Mol. Cells.

[81] Jeong J.S., Kim M.J., Kim I. (2019). The mitochondrial genome of the dung beetle, Copris tripartitus, with mitogenomic comparisons within Scarabaeidae (Coleoptera). Int. J. Biol. Macromol..

[82] Taanman J.-W. (1999). The mitochondrial genome: Structure, transcription, translation and replication. Biochim. Biophys. Acta (BBA) Bioenerg..

[83] Cameron S.L., Whiting M.F. (2008). The complete mitochondrial genome of the tobacco hornworm, Manduca sexta, (Insecta: Lepidoptera: Sphingidae), and an examination of mitochondrial gene variability within butterflies and moths. Gene.

[84] Moriz C., Brown W.M. (1986). Tandem duplications of D-loop and ribosomal RNA sequences in lizard mitochondrial DNA. Science.

[85] Moritz C., Brown W.M. (1987). Tandem duplications in animal mitochondrial DNAs: Variation in incidence and gene content among lizards. Proc. Natl. Acad. Sci. USA.

[86] Levinson G., Gutman G.A. (1987). Slipped-strand mispairing: A major mechanism for DNA sequence evolution. Mol. Biol. Evol..

[87] Bensasson D., Zhang D.-X., Hartl D.L., Hewitt G.M. (2001). Mitochondrial pseudogenes: Evolution’s misplaced witnesses. Trends Ecol. Evol..

[88] Woischnik M., Moraes C.T. (2002). Pattern of Organization of Human Mitochondrial Pseudogenes in the Nuclear Genome. Genome Res..

[89] White D.J., Wolff J.N., Pierson M., Gemmell N. (2008). Revealing the hidden complexities of mtDNA inheritance. Mol. Ecol..

[90] Kang A.R., Kim M.J., Park I.A., Kim K.Y., Kim I. (2015). Extent and divergence of heteroplasmy of the DNA barcoding region in Anapodisma miramae (Orthoptera: Acrididae). Mitochondrial DNA Part A.

[91] Unseld M., Marienfeld J.R., Brandt P., Brennicke A. (1997). The mitochondrial genome of Arabidopsis thaliana contains 57 genes in 366,924 nucleotides. Nat. Genet..

[92] Notsu Y., Masood S., Nishikawa T., Kubo N., Akiduki G., Nakazono M., Hirai A., Kadowaki K. (2002). The complete sequence of the rice (*Oryza sativa* L.) mitochondrial genome: Frequent DNA sequence acquisition and loss during the evolution of flowering plants. Mol. Genet. Genom..

[93] Wang Y., Chen J., Jiang L.Y., Qiao G.X. (2015). Hemipteran mitochondrial genomes: Features, structures and implications for phylogeny. Int. J. Mol. Sci..

[94] Leister D. (2005). Origin, evolution and genetic effects of nuclear insertions of organelle DNA. Trends Genet..

[95] Li K., Liang A.-P. (2018). Hemiptera Mitochondrial Control Region: New Sights into the Structural Organization, Phylogenetic Utility, and Roles of Tandem Repetitions of the Noncoding Segment. Int. J. Mol. Sci..

[96] Wang A.R., Kim J.S., Kim M.J., Kim H.-K., Choi Y.S., Kim I. (2018). Comparative description of mitochondrial genomes of the honey bee Apis (Hymenoptera: Apidae): Four new genome sequences and Apis phylogeny using whole genomes and individual genes. J. Apic. Res..

[97] Dowton M., Cameron S.L., Dowavic J.I., Austin A.D., Whiting M.F. (2009). Characterization of 67 mitochondrial tRNA gene rear-rangements in the Hymenoptera suggests that mitochondrial tRNA gene position is selectively neutral. Mol. Biol. Evol..

[98] Timmermans M.J., Vogler A.P. (2012). Phylogenetically informative rearrangements in mitochondrial genomes of Coleoptera, and monophyly of aquatic elateriform beetles (Dryopoidea). Mol. Phylogenetics Evol..

[99] Cameron S.L. (2014). Insect Mitochondrial Genomics: Implications for Evolution and Phylogeny. Annu. Rev. Entomol..

[100] Urban J.M., Bartlett C.R., Cryan J.R. (2010). Evolution of Delphacidae (Hemiptera: Fulgoroidea): Combined-evidence phylogenetics reveals importance of grass host shifts. Syst. Entomol..

[101] Asche M. (1985). Zur Phylogenie der Delphacidae Leach, 1815 (Homoptera Cicadina Fulgoromorpha), Marburger entomologische. Publikationen.

[102] Boore J.L., Lavrov D., Brown W.M. (1998). Gene translocation links insects and crustaceans. Nature.

[103] Curole J.P., Kocher T.D. (1999). Mitogenomics: Digging deeper with complete mitochondrial genomes. Trends Ecol. Evol..

[104] Rokas A., Holland P.W. (2000). Rare genomic changes as a tool for phylogenetics. Trends Ecol. Evol..

[105] Cameron S.L., Yoshizawa K., Mizukoshi A., Whiting M.F., Johnson K.P. (2011). Mitochondrial genome deletions and minicircles are common in lice (Insecta: Phthiraptera). BMC Genom..

[106] Timmermans M.J., Lees D.C., Simonsen T.J. (2014). Towards a mitogenomic phylogeny of Lepidoptera. Mol. Phylogenetics Evol..

[107] Erixon P., Svennblad B., Britton T., Oxelman B. (2003). Reliability of Bayesian Posterior Probabilities and Bootstrap Frequencies in Phylogenetics. Syst. Biol..

