# Supplementary material for: Complete Mitochondrial Genomes of Metcalfa pruinosa and Salurnis marginella (Hemiptera: Flatidae): Genomic Comparison and Phylogenetic Inference in Fulgoroidea
Source: Curr Issues Mol Biol. 2021 Sep 30;43(3):1391–418. doi: 10.3390/cimb43030099 (PMC8929015; doi:10.3390/cimb43030099)
Supplement: Supplementary file 1 [file cimb-43-00099-s001.zip › Sup. Table 1. Primers for gap filling.pdf]

Table S1

List of primers used to amplify the gapped regions of *Metcalfa pruinosa* H1 and H3 haplotypes.

Fragment name	Primer name	Direction[a]	Sequence (5'- 3')
Long fragments			
LF1	Miseon-LF-COI-F1	F	AGCAGTATTCGCTATTATAGC
〃	Miseon-LF-CYTB-R1	R	TTGATCCAAAATTTCATCATG
LF2	Miseon-LF-CYTB-F2	F	AATAACACTACCATTCTCTATG
〃	Miseon-LF-COI-R2	R	ATTCTTATTATTATTCCCACTAG
gap 1 (*COII* region)			
	Miseon-COII-F (H1)	F	TTTGAACTATCATTCCAGC
	Miseon-COII-R (H1)	R	AATCGTCCTGGAATTGCGTC
gap 2 (3'-end of *ND5*)			
	Miseon-ASN-ND5-F (H1)	F	TAAAAAGAAGCACTTCACTG
	Miseon-ND5-R2 (H1)	R	GTTTTTTTGGTAATCAGGATATTCG
	Miseon-H3-ND5-END-F (H3)	F	TTTAAGAACCATATAGAACT
	Miseon-H3-ND5-END-R (H3)	R	AACCATTGTTTATAGATTGCG
gap 3 (5'-end of *ND5*)			
	Miseon-ND5-2ND-F1 (H1)	F	ATAAACACAGTAACAAGACCTG
	Miseon-ND5-2ND-R2 (H1)	R	TTTTTTTTTCTGCTTGTTATAG
	Miseon-ND5-START-F1 (H3)	F	AAAAACAAAATGGAAATTGAGCTC
	Miseon-ND5-START-R1 (H3)	R	ATTGGTGTTTGTGTAGTTCGTGA
gap 4 (5'-end of *ND4*)			
	Miseon-ND4-F1 (H1)	F	TAAATTGAACCAAATAAAGACC
	Miseon-ND4-R2 (H1)	R	GATTAGTTTTAGTTCGTAAGCA
gap 5 (3'-end of *lrRNA*)			
	Miseon-16S-F2 (H1)	F	AAAAAATTAATCGAATTTCATGTC
	Miseon-12S-R2 (H1)	R	ATTTTAAATGATATTCCACGA

[a]F and R, forward and reverse direction of transcription.

Note that all gap regions except for gap 2 were sequenced after cloning.

With parenthesis indicates the primers designed to target corresponding haplotypes.
