# Supplementary material for: Complete Mitochondrial Genomes of Metcalfa pruinosa and Salurnis marginella (Hemiptera: Flatidae): Genomic Comparison and Phylogenetic Inference in Fulgoroidea
Source: Curr Issues Mol Biol. 2021 Sep 30;43(3):1391–418. doi: 10.3390/cimb43030099 (PMC8929015; doi:10.3390/cimb43030099)
Supplement: Supplementary file 1 [file cimb-43-00099-s001.zip › Sup. Table 10. Blast results of A+T rich region repeat.pdf]

Table S10

BLAST results of the repeat sequences found in the A+T-rich region of Flatidae species.

Species	Repeat A			Repeat B			Repeat C		
	Description	Identity (Query Cover)	GenBank no.	Description	Identity (Query Cover)	GenBank no.	Description	Identity (Query Cover)	GenBank no.
M. pruinosa	*Pan troglodytes* BAC clone RP43-29J18 from chromosome 7 (Chimpanzee)	94.12% (29%)	AC146279	*Raphanus sativus* chromosome 8 (Radish)	100% (83%)	LR778317	*Spirometra erinaceieuropaei*, scaffold0147443 (Cestoda)	100% (100%)	LN165237
							Sarcophilus harrisii, chromosome X (Tasmanian devil)	100% (100%)	LR735560
S. marginella	*Rhodocytophaga* sp. 172606-1 chromosome, complete genome (Bacteria)	84.62% (54%)	CP048222	*Aquila chrysaetos chrysaetos* chromosome 18 (Golden eagle)	85.37% (62%)	LR606198	*Canis lupus* familiaris breed Labrador retriever chromosome 06a (Dog)	100% (100%)	CP050586
G. distinctissima	*Schistosoma mansoni* chromosome W (Trematoda)	96.00% (64%)	HE601631	*Frederiksenia canicola* complete genome (Bacteria)	93.55% (26%)	CP015029	*Crassostrea gigas*, chromosome 10 (Pacific oyster)	100% (100%)	CP048848
							Culicoides sonorensis, scaffold33 (Punkie)	100% (100%)	LN483383
