# Supplementary material for: Complete Mitochondrial Genomes of Metcalfa pruinosa and Salurnis marginella (Hemiptera: Flatidae): Genomic Comparison and Phylogenetic Inference in Fulgoroidea
Source: Curr Issues Mol Biol. 2021 Sep 30;43(3):1391–418. doi: 10.3390/cimb43030099 (PMC8929015; doi:10.3390/cimb43030099)
Supplement: Supplementary file 1 [file cimb-43-00099-s001.zip › Sup. Table 11. Node support.pdf]

Table S11

Node supports for monophyletic families and inter-familial relationships within Fulgoroidea.

Relationships	Dataset (method)			
	1	2	3	4
**Monophyly**				
Ricaniidae (2)	100	1	100	1
Issidae (2)	100	1	100	1
Flatidae (4)	100	1	100	1
Fulgoridae (6)	100	1	100	1
Achilidae (5)	97	1	100	1
Derbidae (1)				
Delphacidae (24)	72	X	93	1
Cixiidae (1)				
**Relationships among Families**				
(Ric + Iss)	72	1	58	0.99
((Ric + Iss) + Fla)	100	1	100	1
(((Ric + Iss) + Fla) + Ful)	42	0.94		
((((Ric + Iss) + Fla) + Ful) + Ach)	99	1		
(((((Ric + Iss) + Fla) + Ful) + Ach) + Der)	100	1		
(((Ric + Iss) + Fla) + Der)			50	0.66
((((Ric + Iss) + Fla) + Der) + Ach)			56	0.66
(((((Ric + Iss) + Fla) + Der) + Ach) + Ful)			100	1
(Del + Cix)	100		98	1
(Asi + Cix)		0.86		
((Asi + Cix) + Del)		1		
((((((Ric + Iss) + Fla) + Ful) + Ach) + Der) + (Del + Cix))	100		100	1
((((((Ric + Iss) + Fla) + Ful) + Ach) + Der) + ((Asi + Cix) + Del))		1		

1. Amino acid sequence-based analysis using the maximum-likelihood (ML) method; 2. Amino acid sequence-based analysis using the Bayesian inference (BI) method; 3. Nucleotide sequence-based analysis using the ML method; and 4. Nucleotide sequence-based analysis using the BI method. Family and subfamily names are abbreviated as follows: Ric, Ricaniidae; Iss, Issidae; Fla, Flatidae; Ful, Fulgoridae; Ach, Achilidae; Der, Derbidae; Del, Delphacidae; Cix, Cixidae; and Asi, subfamily Asiracinae in Delphacidae. Numbers next to family names within parentheses indicate taxon numbers included in the analyses. X, not supported for monophyly.
