# Supplementary material for: Complete Mitochondrial Genomes of Metcalfa pruinosa and Salurnis marginella (Hemiptera: Flatidae): Genomic Comparison and Phylogenetic Inference in Fulgoroidea
Source: Curr Issues Mol Biol. 2021 Sep 30;43(3):1391–418. doi: 10.3390/cimb43030099 (PMC8929015; doi:10.3390/cimb43030099)
Supplement: Supplementary file 1 [file cimb-43-00099-s001.zip › Sup. Table 2. Primers for Salurnis marginella.pdf]

Table S2

Primers used to amplify and sequence the mitochondrial genome of *Salurnis marginella.*

Fragment name	Primer name	Direction[a]	Sequence (5’-3’)	Nucleotide position[b]	Mismatch[c]
Long fragments					
LF1	LF1_F1	F	AGGATCAATAATCAAAAATGACC	1487-1509	0
〃	LF1_R1	R	TGACGAAAAGAATATGATTTCTG	4833-4855	0
LF2	LF2_F1	F	ATATAGTTACAAGAAGCCCATG	4593-4614	1
〃	LF2_R2	R	TTCCTCTGTTTGTCATATAGC	8389-8409	0
LF3	LF3_F1	F	AAAMCCMAWCTGTCTYAAAGTAG	6927-6947	5
〃	LF3_R2	R	TGGCGGTTTTTAAATCTTTTCAG	13892-13914	0
LF4	LF4_F2	F	WAARATCTATAGGGTCTTATCG	12679-12700	2
〃	LF4_R1	R	TATGATAGATCTAATACCTGC	1812-1832	0
Short fragments					
SF1	SF1_F2	F	ATCATTACTTATTGCGAGATC	1682-1702	0
〃	SF1_R2	R	TTTTAATWCCWGTRGGTACTGC	2293-2313	3
SF2	SF2_F2	F	TGAGCYCATCAYATATTTACTG	2217-2238	4
〃	SF2_R2	R	AARTYYWTTGCACTWWTCTGCC	2907-2928	6
SF3	SF3_F3	F	ACCYCGWCGATAYTCAG	2663-2679	3
〃	SF3_R2	R	AGMKGATGTAAYAAKWATTCG	3401-3421	6
SF4	SF4_F_MJ	F	TTGGTCACCAATGATACTGATC	3259-3279	0
〃	SF4_R_MJ	R	TTTATTGGTAGGATGATTGC	3997-4016	0
SF5	SF5_F2	F	CAAAYATCRCCMATTATRTGAAC	3779-3801	4
〃	SF5_R2	R	AGTAGCAAAAACATATGCTTG	4519-4539	0
SF6	SF6_F2	F	ATAATTGCAGGACACTTACTG	4399-4419	0
〃	SF6_R1	R	TGTTSWTCCATAAATTGAGTC	5135-5155	4
SF7	SF7_F2	F	AGGAGTTTCAATTACCTGAGC	4987-5007	0
〃	SF7_R2	R	WGARAATGGTTTTCGWGCTG	5578-5597	4
SF8	SF8_F2	F	TTTAYATYWSARTTTACTGATG	5322-5343	6
〃	SF8_R1	R	AWWYCAWTTTTTTCATTAACAGTG	5938-5961	5
SF9	SF9_F1	F	TTAAWAATAACAKYTTYATTGC	5796-5817	4
〃	SF9_R1	R	TWCTGGKTTTTTWTTAWRTCG	6343-6363	6
SF10	SF10_F1	F	ARTRTYAAACTTTTAWAATTAAGC	6126-6147	7
〃	SF10_R2	R	TTTTTTTGGTAATCARGATATTCG	6871-6894	1
SF11	SF11_F2	F	TATTTCTAAAACAAAATCCYTAG	6747-6769	5
〃	SF11_R2	R	TTTATRAATCGTTTTGGTGATG	7394-7415	3
SF12	SF12_F2	F	AGTGGGAGCTGCTATAGC	7212-7229	3
〃	SF12_R2	R	TTWTKTTTYTTCATTTATWTCCTG	7943-7966	5
SF13	SF13_F2	F	ATAAAAAWTAWAGAAAYYCARTC	7660-7682	5
〃	SF13_R2	R	AATTATATTTATATTGGGACATGG	8314-8337	0
SF14	SF14_F2	F	AAAAYAAATTAATAGAAGGAGGAC	8144-8167	2
〃	SF14_R2	R	ATACCCTTTTTGCCTC	8779-8794	0
SF15	SF15_F1	F	TCCTAATTTTAATAAGACTCCAGC	8556-8579	0
〃	SF15_R2	R	ATTTCTTATRTTTTTGGWSTAG	9065-9086	3
SF16	SF16_F2	F	CCGAAAATAATTAAAAACACAGG	8845-8867	0
〃	SF16_R1	R	TTTAYTWAGWTTRRTTTTAGTTCG	9439-9463	6
SF17	SF17_F2	F	AGAWAKTARRWAATGMTTACG	9421-9441	7
〃	SF17_R2	R	TSWWYTTGAAATATTTGTWACTG	10066-10088	5
SF18	SF18_F2	F	TTYATATAYATRTCAAGARTSG	9807-9828	6
〃	SF18_R1	R	TTATTACRAARAATATTGAKGC	10363-10384	3
SF19	SF19_F1	F	TAATAATTCAAATYWTWWCAGG	10226-10247	4
〃	SF19_R2	R	TTWGCTGGTGTAAARTTTTCTGG	10862-10883	4
SF20	SF20_F1	F	TWGATAACCCAACACTRAACCG	10613-10634	4
〃	SF20_R2	R	TKTCTTGAARGTGTTATTTACC	11368-11389	3
SF21	SF21_F2	F	AAGAACCWTAYATYTTAACAGG	11135-11156	3
〃	SF21_R2	R	TTTTTTTCTTGTTGTYTKGCTG	11657-11678	3
SF22	SF22_F2	F	TTRAAMCCYGAAACTAATTCAG	11596-11617	4
〃	SF22_R2	R	WGCTTTTTTTACTTTATTTGAGCG	12157-12180	2
SF23	SF23_F1	F	YAGAATTTGAAGATCAWCCAG	11855-11875	6
〃	SF23_R2	R	ATACCTTAGGGATAACAGC	12509-12527	0
SF24	SF24_F2	F	YACGCCGGTCTGAACTCAG	12368-12386	1
〃	SF24_R1	R	GGCAAATTTTKWTTTTCRCCT	12914-12934	6
SF25	SF25_F1	F	AAAARACTAATTATTATGCTACC	12803-12825	1
〃	SF25_R2	R	TTTACAATAAAATCTTGTTGTTCG	13513-13536	0
SF26	SF26_F2	F	TAACCAGATAWMAAATTAAKCG	13207-13228	5
〃	SF26_R2	R	AATTTTCTTGTATATCTCTGTCG	13824-13846	0
SF27	SF27_F2	F	TCTCACTGTATGAGAATGAC	13613-13631	0
〃	SF27_R2	R	AAAAATTTTGTGCCAGCATTCG	14111-14132	1
SF28	SF28_F1	F	TAACAGGGTATCTAATCCTGG	13969-13989	0
〃	SF28_R1	R	YCGGGGTATGAACCCRTTAGC	170-190	3
SF29	SF29_F2	F	AAAAGAAATCAAAATTCTTCATGC	108-131	2
〃	SF29_R1	R	TRTTATTGGGGGTATTCCTC	924-943	5
SF30	SF30_F1	F	RTCMATTTMWAAYAYRCCATG	724-744	9
〃	SF30_R2	R	ATTATTCCTACTATTCCCGATC	1429-1450	0

[a]F and R, forward and reverse direction of transcription.
[b]Nucleotide positions are with respect to the *S. marginella* mitochondrial genome.
[c]Mismatches are with respect to the *S. marginella* mitochondrial genome.
