# Supplementary material for: Complete Mitochondrial Genomes of Metcalfa pruinosa and Salurnis marginella (Hemiptera: Flatidae): Genomic Comparison and Phylogenetic Inference in Fulgoroidea
Source: Curr Issues Mol Biol. 2021 Sep 30;43(3):1391–418. doi: 10.3390/cimb43030099 (PMC8929015; doi:10.3390/cimb43030099)
Supplement: Supplementary file 1 [file cimb-43-00099-s001.zip › Sup. Table 3. Summary of Fulgoroidea-MJ.pdf]

Table S3
Summary of mitochondrial genomes of Fulgoroidea.

Gene	Strand	Anticodon	Start codon	Stop codon	*Metcalfa pruinosa* (H1)	*Metcalfa pruinosa* (H3)	*Salurnis marginella*	*Geisha distinctissima*‡	*Pyrops candelaria*
trnI	+	GAT			1-65 (65)	1-65 (65)	1-62 (62)	1-63 (63)	1-64 (64)
trnQ	-	TTG			69-137 (69)	69-137 (69)	81-149 (69)	63-131 (69)	74-143 (70)
trnM	+	CAT			137-202 (66)	137-202 (66)	149-214 (66)	131-196 (66)	148-211 (64)
ND2	+		ATT[a]/ATA[b]	TAA[1]/TAG[2]	203-1180 (978)[a, 1]	203-1180 (978)[a, 1]	215-1180 (966)[a, 1]	197-1162 (966)[b, 1]	212-1180 (969)[a, 1]
trnW	+	TCA			1179-1246 (68)	1179-1246 (68)	1179-1243 (65)	1161-1224 (64)	1214-1281 (68)
trnC	-	GCA			1239-1302 (64)	1239-1302 (64)	1236-1295 (60)	1217-1279 (63)	1274-1341 (68)
trnY	-	GTA			1310-1373 (64)	1310-1373 (64)	1296-1356 (61)	1283-1344 (62)	1342-1404 (63)
COI	+		ATC[a]/ATG[b]/ATA[c]/CTG[d]	TAA[1]/T[2]	1374-2918 (1545)[a, 1]	1374-2918 (1545)[a, 1]	1362-2897 (1536)[b, 1]	1351-2884 (1534)[b, 2]	1407-2949 (1543)[a, 2]
$trnL_2$	+	TAA			2921-2983 (63)	2921-2983 (63)	2898-2959 (62)	2885-2949 (65)	2950-3015 (66)
COII	+		ATA[a]/ATG[b]/ATT[c]	TAA[1]/T[2]/TAG[3]	2984-3661 (678)[a, 1]	2984-3661 (678)[a, 1]	2960-3640 (681)[a, 1]	2951-3637 (687)[a, 1]	3024-3689 (666)[b, 1]
trnK	+	CTT			3672-3741 (70)	3672-3741 (70)	3641-3709 (69)	3640-3706 (67)	3692-3762 (71)
trnD	+	GTC			3742-3810 (69)	3742-3810 (69)	3710-3772 (63)	3707-3771 (65)	3763-3825 (63)
ATP8	+		ATT[a]/ATA[b]/ATC[c]	TAA[1]/TAG[2]	3811-3966 (156)[a, 1]	3811-3966 (156)[a, 1]	3773-3925 (153)[a, 1]	3772-3924 (153)[a, 1]	3826-3984 (159)[a, 1]
ATP6	+		ATG[a]/ATA[b]/ATT[c]	T[1]/TAG[2]	3960-4611 (652)[a, 1]	3960-4611 (652)[a, 1]	3919-4570 (652)[a, 1]	3918-4569 (652)[a, 1]	3978-4629 (652)[a, 1]
COIII	+		ATG[a]/ATT[b]	TAG[1]/T[2]/TAA[3]	4612-5394 (783)[a, 3]	4612-5394 (783)[a, 3]	4571-5353 (783)[a, 3]	4570-5350 (781)[a, 2]	4630-5412 (783)[a, 3]
trnG	+	TCC			5401-5465 (65)	5401-5465 (65)	5355-5415 (61)	5351-5412 (62)	5419-5481 (63)
ND3	+		ATG[a]/ATT[b]/ATC[c] /ATA[d]	TAA[1]/TA[2]/TAG[3] /T[4]	5467-5817 (351)[a, 1]	5467-5817 (351)[a, 1]	5451-5786 (336)[b, 1]	5413-5760 (348)[b, 1]	5482-5829 (348)[b, 1]
trnA	+	TGC			5816-5883 (68)	5816-5883 (68)	5786-5848 (63)	5761-5825 (65)	5847-5917 (71)
trnR	+	TCG			5901-5964 (64)	5901-5964 (64)	5849-5909 (61)	5826-5886 (61)	5922-5986 (65)
trnN	+	GTT			5990-6054 (65)	5990-6054 (65)	5911-5973 (63)	5892-5953 (62)	5987-6051 (65)
$trnS_1$	+	GCT			6056-6116 (61)	6056-6116 (61)	5972-6031 (60)	5954-6014 (61)	6050-6111 (62)
trnE	+	TTC			6120-6184 (65)	6120-6184 (65)	6034-6095 (62)	6017-6078 (62)	6114-6174 (61)
trnF	-	GAA			6197-6261 (65)	6197-6261 (65)	6094-6156 (63)	6082-6145 (64)	6173-6237 (65)
ND5	-		ATG[a]/ATT[b]/TTG[c] /ATA[d]/GTG[e]	TAA[1]/TAG[2]/T[3]	6268-7950 (1683)[a, 1]	6268-7950 (1683)[a, 1]	6156-7835 (1680)[c, 1]	6136-7782 (1647)[a, 2]	6238-7909 (1672)[a, 3]
trnH	-	GTG			7952-8016 (65)	7952-8016 (65)	7836-7897 (62)	7792-7858 (67)	7910-7971 (62)
ND4	-		ATG[a]/ATT[b]/ATA[c]	TAG[1]/T[2]/TAA[3]/TA[4]	8031-9362 (1332)[a, 1]	8031-9362 (1332)[a, 1]	7898-9221 (1324)[a, 2]	7859-9179 (1321)[a, 2]	7976-9290 (1315)[a, 2]
ND4L	-		ATG[a]/ATT[b]	TAA[1]/TAG[2]	9362-9634 (273)[a, 1]	9362-9634 (273)[a, 1]	9223-9495 (273)[a, 1]	9188-9469 (282)[a, 2]	9284-9577 (294)[b, 1]
trnT	+	TGT			9637-9700 (64)	9637-9700 (64)	9498-9558 (61)	9472-9532 (61)	9594-9657 (64)
trnP	-	TGG			9708-9773 (66)	9708-9773 (66)	9560-9622 (63)	9536-9597 (62)	9661-9721 (61)
ND6	+		ATA[a]/ATT[b]/ATC[c]	TAA[1]/T[2]	9775-10275 (501)[a, 1]	9775-10275 (501)[a, 1]	9624-10121 (498)[a, 1]	9588-10091 (504)[a, 1]	9723-10217 (495)[b, 1]
CytB	+		ATG[a]/ATA[b]	TAA[1]/TAG[2]/T[3]	10280-11404 (1125)[a, 1]	10280-11404 (1125)[a, 1]	10114-11235 (1122)[a, 1]	10084-11205 (1122)[a, 1]	10221-11333 (1113)[a, 1]
$trnS_2$	+	TGA			11420-11482 (63)	11420-11482 (63)	11235-11295 (61)	11206-11266 (61)	11351-11412 (62)
ND1	-		ATG[a]/GTG[b]	TAA[1]/T[2]/TA[3] /TAG[4]	11492-12442 (951)[a, 1]	11492-12442 (951)[a, 1]	11289-12230 (942)[a, 1]	11273-12205 (933)[a, 1]	11406-12359 (954)[b, 1]
$trnL_1$	-	TAG			12444-12508 (65)	12444-12508 (65)	12232-12293 (62)	12207-12268 (62)	12361-12423 (63)
lrRNA	-				12509-13734 (1226)	12509-13734 (1226)	12294-13497 (1205)	12269-13466 (1198)	12424-13637 (1214)
trnV	-	TAC			13735-13807 (73)	13735-13807 (73)	13498-13567 (70)	13467-13540 (74)	13638-13712 (75)
srRNA	-				13808-14524 (717)	13808-14524 (717)	13567-14290 (724)	13541-14269 (729)	13713-14429 (717)
A+T–rich region					14525-16312 (1788)	14525-16314 (1790)	14291-16126 (1836)	14270-15971 (1702)	14430-16021 (1592)

Gene	Strand	Anticodon	Start codon	Stop codon	*Lycorma delicatula* (Henan, China)	*Lycorma delicatula* (China)	*Lycorma delicatula* (Korea)	*Aphaena* (Callidepsa) *amabilis*	*Aphaena* (Aphaena) *discolor nigrotibiata*
trnI	+	GAT			1-64 (64)	1-64 (64)	1-64 (64)	1-63 (63)	1-64 (64)
trnQ	-	TTG			67-135 (69)	67-135 (69)	67-135 (69)	66-134 (69)	67-135 (69)
trnM	+	CAT			135-197 (63)	135-197 (63)	135-197 (66)	134-197 (64)	135-197 (63)
ND2	+		ATT[a]/ATA[b]	TAA[1]/TAG[2]	198-1163 (966)[a, 1]	198-1163 (966)[a, 1]	198-1163 (966)[a, 1]	198-1166 (969)[a, 1]	198-1166 (969)[a, 1]
trnW	+	TCA			1166-1229 (64)	1166-1229 (64)	1166-1229 (64)	1165-1226 (62)	1169-1230 (62)
trnC	-	GCA			1222-1283 (62)	1222-1283 (62)	1222-1283 (62)	1219-1281 (63)	1223-1282 (60)
trnY	-	GTA			1284-1344 (61)	1284-1345 (62)	1284-1344 (61)	1291-1352 (62)	1286-1347 (62)
COI	+		ATC[a]/ATG[b]/ATA[c]/CTG[d]	TAA[1]/T[2]	1349-2885 (1537)[b, 2]	1350-2886 (1537)[b, 2]	1349-2885 (1537)[b, 2]	1357-2890 (1534)[b, 2]	1353-2886 (1534)[b, 2]
$trnL_2$	+	TAA			2886-2953 (68)	2887-2954 (68)	2886-2953 (68)	2891-2959 (69)	2887-2954 (68)
COII	+		ATA[a]/ATG[b]/ATT[c]	TAA[1]/T[2]/TAG[3]	2954-3625 (672)[a, 1]	2955-3626 (672)[a, 1]	2954-3625 (672)[a, 1]	2960-3637 (678)[b, 1]	2955-3632 (678)[b, 1]
trnK	+	CTT			3636-3705 (70)	3637-3706 (70)	3636-3705 (70)	3647-3716 (70)	3642-3711 (70)
trnD	+	GTC			3706-3768 (63)	3707-3770 (64)	3706-3769 (64)	3717-3779 (63)	3712-3779 (68)
ATP8	+		ATT[a]/ATA[b]/ATC[c]	TAA[1]/TAG[2]	3769-3930 (162)[a, 1]	3771-3932 (162)[a, 1]	3770-3931 (162)[a, 1]	3780-3941 (162)[a, 1]	3780-3935 (156)[b, 1]
ATP6	+		ATG[a]/ATA[b]/ATT[c]	T[1]/TAG[2]	3924-4575 (652)[a, 1]	3926-4577 (652)[a, 1]	3925-4576 (652)[a, 1]	3935-4592 (658)[a, 1]	3929-4583 (655)[a, 1]
COIII	+		ATG[a]/ATT[b]	TAG[1]/T[2]/TAA[3]	4576-5358 (783)[a, 3]	4578-5360 (783)[a, 3]	4577-5359 (783)[a, 3]	4593-5375 (783)[a, 3]	4584-5366 (783)[a, 3]
trnG	+	TCC			5364-5427 (64)	5363-5426 (64)	5362-5425 (64)	5379-5443 (65)	5374-5436 (63)
ND3	+		ATG[a]/ATT[b]/ATC[c] /ATA[d]	TAA[1]/TA[2]/TAG[3] /T[4]	5428-5769 (342)[c, 1]	5427-5773 (347)[b, 2]	5426-5773 (348)[b, 1]	5444-5791 (348)[b, 1]	5437-5784 (348)[b, 1]
trnA	+	TGC			5795-5859 (65)	5774-5838 (65)	5773-5837 (65)	5798-5862 (65)	5784-5848 (65)
trnR	+	TCG			5856-5927 (72)	5839-5905 (67)	5838-5904 (67)	5864-5924 (61)	5850-5910 (61)
trnN	+	GTT			5927-5988 (62)	5905-5966 (62)	5904-5965 (62)	5932-5993 (62)	5919-5980 (62)
$trnS_1$	+	GCT			5987-6046 (60)	5966-6023 (58)	5964-6023 (60)	5993-6050 (58)	5980-6039 (60)
trnE	+	TTC			6045-6106 (62)	6023-6085 (63)	6022-6084 (63)	6050-6115 (66)	6039-6100 (62)
trnF	-	GAA			6105-6168 (64)	6084-6146 (63)	6083-6145 (63)	6114-6176 (63)	6099-6161 (63)
ND5	-		ATG[a]/ATT[b]/TTG[c] /ATA[d]/GTG[e]	TAA[1]/TAG[2]/T[3]	6159-7820 (1662)[a, 2]	6147-7824 (1678)[a, 3]	6146-7823 (1678)[a, 3]	6178-7885 (1708)[a, 3]	6164-7871 (1708)[a, 3]
trnH	-	GTG			7820-7883 (64)	7824-7887 (64)	7823-7886 (64)	7886-7947 (62)	7872-7933 (62)
ND4	-		ATG[a]/ATT[b]/ATA[c]	TAG[1]/T[2]/TAA[3]/TA[4]	7868-9208 (1341)[a, 3]	7888-9211 (1324)[a, 2]	7887-9210 (1324)[a, 2]	7949-9272 (1324)[a, 2]	7935-9258 (1324)[a, 2]
ND4L	-		ATG[a]/ATT[b]	TAA[1]/TAG[2]	9202-9477 (276)[a, 1]	9205-9480 (276)[a, 1]	9204-9479 (276)[a, 1]	9266-9541 (276)[a, 1]	9252-9527 (276)[a, 1]
trnT	+	TGT			9485-9550 (66)	9488-9553 (66)	9487-9552 (66)	9549-9613 (65)	9535-9599 (65)
trnP	-	TGG			9554-9615 (62)	9557-9618 (62)	9556-9617 (62)	9620-9681 (62)	9602-9664 (63)
ND6	+		ATA[a]/ATT[b]/ATC[c]	TAA[1]/T[2]	9617-10117 (501)[b, 1]	9620-10120 (501)[b, 1]	9619-10119 (501)[b, 1]	9683-10183 (501)[a, 1]	9666-10166 (501)[b, 1]
CytB	+		ATG[a]/ATA[b]	TAA[1]/TAG[2]/T[3]	10110-11228 (1119)[a, 1]	10113-11231 (1119)[a, 1]	10124-11230 (1107)[b, 1]	10176-11294 (1119)[a, 1]	10159-11280 (1122)[a, 1]
$trnS_2$	+	TGA			11234-11297 (64)	11237-11300 (64)	11236-11299 (64)	11296-11358 (63)	11281-11342 (62)
ND1	-		ATG[a]/GTG[b]	TAA[1]/T[2]/TA[3] /TAG[4]	11290-12234 (945)[a, 1]	11293-12237 (945)[a, 1]	11292-12236 (945)[a, 1]	11362-12301 (940)[a, 2]	11347-12285 (939)[a, 1]
$trnL_1$	-	TAG			12236-12298 (63)	12239-12301 (63)	12238-12300 (63)	12303-12364 (62)	12287-12348 (62)
lrRNA	-				12299-13512 (1214)	12302-13513 (1212)	12301-13509 (1209)	12365-13574 (1210)	12349-13558 (1210)
trnV	-	TAC			13513-13583 (71)	13514-13575 (62)	13510-13571 (62)	13575-13638 (64)	13559-13619 (61)
srRNA	-				13584-14304 (721)	13576-14367 (792)	13569-14303 (735)	13639-14368 (730)	13620-14352 (733)
A+T–rich region					14305-15946 (1642)	14368-15410 (1043)	14304-15798 (1495)	14369-16237 (1869)	14353-16116 (1764)

Gene	Strand	Anticodon	Start codon	Stop codon	*Betatropis formosana*	*Magadhaideus luodiana* sp. nov	*Peltatavertexalis horizontalis* sp. nov	*Plectoderini* sp.	*Paracatonidia* sp.
trnI	+	GAT			1-64 (64)	1-65 (65)	1-64 (64)	1-64 (64)	1-64 (64)
trnQ	-	TTG			62-130 (69)	68-136 (69)	62-130 (69)	62-130 (69)	62-130 (69)
trnM	+	CAT			130-192 (63)	136-201 (66)	130-194 (65)	130-196 (67)	130-193 (64)
ND2	+		ATT[a]/ATA[b]	TAA[1]/TAG[2]	193-1161 (969)[a, 1]	202-1170 (969)[a, 1]	195-1160 (966)[a, 1]	197-1162 (966)[a, 1]	194-1162 (969)[a, 1]
trnW	+	TCA			1160-1223 (64)	1169-1239 (71)	1159-1221 (63)	1161-1227 (67)	1161-1225 (65)
trnC	-	GCA			1216-1278 (63)	1232-1298 (67)	1214-1277 (64)	1220-1282 (63)	1218-1278 (61)
trnY	-	GTA			1291-1352 (62)	1307-1369 (63)	1283-1344 (62)	1291-1352 (62)	1282-1343 (62)
COI	+		ATC[a]/ATG[b]/ATA[c]/CTG[d]	TAA[1]/T[2]	1369-2902 (1534)[b, 2]	1370-2903 (1534)[b, 2]	1345-2878 (1534)[b, 2]	1355-2888 (1534)[b, 2]	1344-2877 (1534)[b, 2]
trnL$_2$	+	TAA			2903-2966 (64)	2904-2965 (62)	2879-2940 (62)	2889-2951 (63)	2878-2943 (66)
COII	+		ATA[a]/ATG[b]/ATT[c]	TAA[1]/T[2]/TAG[3]	2967-3636 (670)[c, 2]	2966-3635 (670)[c, 2]	2941-3610 (670)[a, 2]	2952-3620 (669)[a, 1]	2944-3616 (673)[b, 2]
trnK	+	CTT			3637-3704 (68)	3636-3704 (69)	3611-3680 (70)	3622-3690 (69)	3617-3686 (70)
trnD	+	GTC			3705-3766 (62)	3705-3766 (62)	3681-3743 (63)	3691-3762 (72)	3687-3748 (62)
ATP8	+		ATT[a]/ATA[b]/ATC[c]	TAA[1]/TAG[2]	3767-3940 (174)[b, 1]	3767-3931 (165)[b, 1]	3744-3902 (159)[b, 2]	3763-3924 (162)[b, 1]	3749-3901 (153)[b, 1]
ATP6	+		ATG[a]/ATA[b]/ATT[c]	T[1]/TAG[2]	3934-4585 (652)[a, 1]	3925-4576 (652)[a, 1]	3896-4547 (652)[a, 1]	3918-4569 (652)[a, 1]	3895-4546 (652)[a, 1]
COIII	+		ATG[a]/ATT[b]	TAG[1]/T[2]/TAA[3]	4586-5368 (783)[a, 3]	4577-5357 (781)[a, 2]	4548-5330 (783)[a, 3]	4570-5350 (781)[a, 2]	4547-5327 (781)[a, 2]
trnG	+	TCC			5368-5428 (61)	5358-5419 (62)	5331-5395 (65)	5351-5414 (64)	5328-5388 (61)
ND3	+		ATG[a]/ATT[b]/ATC[c] /ATA[d]	TAA[1]/TA[2]/TAG[3] /T[4]	5435-5776 (342)[b, 3]	5420-5765 (346)[b, 4]	5396-5743 (348)[d, 1]	5415-5762 (348)[d, 1]	5389-5736 (348)[b, 1]
trnA	+	TGC			5775-5840 (66)	5766-5830 (65)	5747-5809 (63)	5762-5829 (68)	5736-5800 (65)
trnR	+	TCG			5847-5908 (62)	5831-5889 (59)	5813-5872 (60)	5835-5895 (61)	5801-5862 (62)
trnN	+	GTT			5911-5972 (62)	5902-5965 (64)	5874-5936 (63)	5902-5965 (64)	5863-5925 (63)
trnS$_1$	+	GCT			5973-6032 (60)	5966-6020 (55)	5937-5992 (56)	5966-6025 (60)	5925-5987 (63)
trnE	+	TTC			6039-6102 (64)	6029-6091 (63)	5993-6056 (64)	6026-6088 (63)	5991-6052 (62)
trnF	-	GAA			6105-6167 (63)	6091-6153 (63)	6061-6127 (67)	6088-6151 (64)	6056-6122 (67)
ND5	-		ATG[a]/ATT[b]/TTG[c] /ATA[d]/GTG[e]	TAA[1]/TAG[2]/T[3]	6178-7875 (1698)[b, 1]	6156-7850 (1695)[c, 2]	6122-7828 (1707)[a, 2]	6154-7860 (1707)[a, 2]	6103-7824 (1722)[d, 1]
trnH	-	GTG			7881-7943 (63)	7859-7920 (62)	7845-7906 (62)	7864-7925 (62)	7818-7880 (63)
ND4	-		ATG[a]/ATT[b]/ATA[c]	TAG[1]/T[2]/TAA[3]/TA[4]	7945-9289 (1345)[a, 2]	7922-9245 (1324)[a, 2]	7908-9189 (1282)[b, 2]	7927-9229 (1303)[a, 2]	7882-9121 (1240)[a, 2]
ND4L	-		ATG[a]/ATT[b]	TAA[1]/TAG[2]	9283-9555 (273)[a, 1]	9239-9511 (273)[a, 1]	9235-9507 (273)[a, 1]	9278-9550 (273)[a, 1]	9191-9463 (273)[a, 1]
trnT	+	TGT			9558-9620 (63)	9514-9575 (62)	9510-9573 (64)	9552-9613 (62)	9467-9529 (63)
trnP	-	TGG			9628-9689 (62)	9578-9640 (63)	9574-9636 (63)	9614-9676 (63)	9531-9595 (65)
ND6	+		ATA[a]/ATT[b]/ATC[c]	TAA[1]/T[2]	9691-10197 (507)[a, 1]	9642-10142 (501)[b, 1]	9638-10144 (507)[a, 1]	9678-10181 (504)[b, 1]	9597-10091 (495)[c, 1]
CytB	+		ATG[a]/ATA[b]	TAA[1]/TAG[2]/T[3]	10190-11311 (1122)[a, 1]	10135-11256 (1122)[a, 1]	10137-11258 (1122)[a, 1]	10174-11295 (1122)[a, 1]	10098-11222 (1125)[a, 1]
trnS$_2$	+	TGA			11311-11376 (66)	11256-11319 (64)	11258-11321 (64)	11295-11357 (63)	11222-11285 (64)
ND1	-		ATG[a]/GTG[b]	TAA[1]/T[2]/TA[3] /TAG[4]	11376-12320 (945)[a, 1]	11316-12258 (943)[b, 2]	11320-12261 (942)[a, 1]	11351-12295 (945)[b, 1]	11291-12234 (944)[b, 3]
trnL$_1$	-	TAG			12322-12384 (63)	12260-12325 (66)	12263-12325 (63)	12297-12360 (64)	12236-12301 (66)
lrRNA	-				12403-13584 (1182)	12329-13541 (1213)	12326-12530 (205)	12364-13562 (1199)	12302-13499 (1198)
trnV	-	TAC			13598-13660 (63)	13549-13612 (64)	13539-13602 (64)	13578-13640 (63)	13518-13582 (65)
srRNA	-				13658-14383 (726)	13610-14335 (726)	13600-14328 (729)	13638-14366 (729)	13580-14305 (726)
A+T–rich region					14384-16161 (1778)	14336-15885 (1550)	14329-15787 (1459)	14367-16216 (1850)	14306-15214 (909)

Gene	Strand	Anticodon	Start codon	Stop codon	*Pentastiridius* sp.‡	*Lydda* sp.‡	*Sivaloka damnosus*	*Sivaloka* sp.‡	*Ricania marginalis*
trnI	+	GAT			1-68 (68)	1-63 (63)	1-66 (66)	1-63 (63)	1-64 (64)
trnQ	-	TTG			66-132 (67)	65-131 (67)	67-135 (69)	63-131 (69)	69-137 (69)
trnM	+	CAT			131-196 (66)	132-196 (65)	135-200 (66)	131-193 (63)	152-216 (65)
ND2	+		ATT[a]/ATA[b]	TAA[1]/TAG[2]	197-1156 (960)[a, 1]	197-1156 (960)[a, 1]	201-1166 (966)[a, 2]	194-1159 (966)[a, 1]	217-1182 (966)[a, 1]
trnW	+	TCA			1155-1222 (68)	1155-1217 (63)	1167-1230 (64)	1159-1219 (61)	1210-1273 (64)
trnC	-	GCA			1215-1276 (62)	1210-1274 (65)	1222-1284 (63)	1212-1272 (61)	1266-1328 (63)
trnY	-	GTA			1277-1337 (61)	1277-1337 (61)	1301-1367 (67)	1273-1336 (64)	1341-1402 (62)
COI	+		ATC[a]/ATG[b]/ATA[c]/CTG[d]	TAA[1]/T[2]	1336-2869 (1534)[d, 2]	1339-2872 (1534)[b, 2]	1377-2912 (1536)[b, 1]	1338-2871 (1534)[b, 2]	1404-2945 (1542)[c, 1]
$trnL_2$	+	TAA			2874-2937 (64)	2874-2935 (62)	2908-2970 (63)	2873-2935 (63)	2947-3009 (63)
COII	+		ATA[a]/ATG[b]/ATT[c]	TAA[1]/T[2]/TAG[3]	2938-3606 (669)[a, 1]	2936-3601 (666)[a, 1]	2971-3642 (672)[a, 1]	2936-3607 (672)[a, 1]	3010-3682 (673)[c, 2]
trnK	+	CTT			3608-3678 (71)	3602-3672 (71)	3643-3712 (70)	3608-3677 (70)	3683-3752 (70)
trnD	+	GTC			3679-3742 (64)	3674-3738 (65)	3713-3774 (62)	3678-3739 (62)	3755-3818 (64)
ATP8	+		ATT[a]/ATA[b]/ATC[c]	TAA[1]/TAG[2]	3743-3850 (108)[a, 1]	3739-3888 (150)[b, 1]	3775-3927 (153)[a, 1]	3740-3892 (153)[a, 1]	3819-3980 (162)[b, 1]
ATP6	+		ATG[a]/ATA[b]/ATT[c]	T[1]/TAG[2]	3844-4498 (655)[b, 1]	3883-4533 (651)[a, 1]	3921-4572 (652)[a, 1]	3886-4537 (652)[a, 1]	3977-4654 (678)[b, 2]
COIII	+		ATG[a]/ATT[b]	TAG[1]/T[2]/TAA[3]	4499-5281 (783)[a, 3]	4534-5314 (781)[a, 2]	4573-5355 (783)[a, 3]	4538-5320 (783)[a, 3]	4627-5409 (783)[a, 1]
trnG	+	TCC			5281-5343 (63)	5315-5376 (62)	5376-5436 (61)	5321-5381 (61)	5413-5473 (61)
ND3	+		ATG[a]/ATT[b]/ATC[c] /ATA[d]	TAA[1]/TA[2]/TAG[3] /T[4]	5344-5694 (351)[b, 1]	5377-5724 (348)[b, 1]	5437-5784 (348)[b, 1]	5381-5728 (348)[b,1]	5474-5821 (348)[d, 1]
trnA	+	TGC			5694-5758 (65)	5724-5789 (66)	5791-5857 (67)	5728-5791 (64)	5827-5891 (65)
trnR	+	TCG			5758-5819 (62)	5790-5850 (61)	5858-5923 (66)	5792-5851 (60)	5892-5963 (72)
trnN	+	GTT			5818-5882 (65)	5850-5912 (63)	5937-6002 (66)	5852-5913 (62)	5961-6028 (68)
$trnS_1$	+	GCT			5882-5951 (70)	5913-5969 (57)	6002-6060 (59)	5913-5971 (59)	6028-6088 (61)
trnE	+	TTC			5951-6015 (65)	5975-6038 (64)	6069-6130 (62)	5971-6032 (62)	6090-6156 (67)
trnF	-	GAA			6016-6080 (65)	6037-6100 (64)	6135-6199 (65)	6031-6094 (64)	6165-6230 (66)
ND5	-		ATG[a]/ATT[b]/TTG[c] /ATA[d]/GTG[e]	TAA[1]/TAG[2]/T[3]	6165-7706 (1542)[d, 1]	6101-7691 (1591)[d, 3]	6206-7864 (1659)[b, 1]	6093-7673 (1581)[b, 1]	6237-7916 (1680)[b, 1]
trnH	-	GTG			7847-7908 (62)	-	7870-7930 (61)	-	7918-7981 (64)
ND4	-		ATG[a]/ATT[b]/ATA[c]	TAG[1]/T[2]/TAA[3]/TA[4]	7909-9213 (1305)[b, 2]	7809-9122 (1314)[b, 1]	7931-9245 (1315)[a, 2]	7771-8997 (1227)[c, ?]	7982-9302 (1321)[a, 2]
ND4L	-		ATG[a]/ATT[b]	TAA[1]/TAG[2]	9219-9494 (276)[a, 1]	9128-9400 (273)[a, 1]	9239-9511 (273)[a, 1]	9149-9421 (273)[a, 1]	9296-9565 (270)[a, 1]
trnT	+	TGT			9506-9568 (63)	9403-9465 (63)	9514-9576 (63)	9424-9486 (63)	9568-9632 (65)
trnP	-	TGG			9568-9631 (64)	9465-9528 (64)	9577-9639 (63)	9487-9549 (63)	9644-9709 (66)
ND6	+		ATA[a]/ATT[b]/ATC[c]	TAA[1]/T[2]	9633-10127 (495)[a, 1]	9530-10027 (498)[c, 1]	9641-10132 (492)[a, 1]	9551-10039 (489)[a, 1]	9711-10205 (495)[c, 1]
CytB	+		ATG[a]/ATA[b]	TAA[1]/TAG[2]/T[3]	10131-11246 (1116)[a, 1]	10020-11144 (1125)[a, 1]	10125-11255 (1131)[a, 1]	10032-11153 (1122)[a, 2]	10198-11316 (1119)[a, 1]
$trnS_2$	+	TGA			11248-11309 (62)	11143-11205 (63)	11255-11319 (65)	11152-11213 (62)	11303-11364 (62)
ND1	-		ATG[a]/GTG[b]	TAA[1]/T[2]/TA[3] /TAG[4]	-	11206-12139 (934)[a, 2]	11324-12253 (930)[a, 4]	11207-12145 (939)[a, 1]	11361-12296 (936)[a, 1]
$trnL_1$	-	TAG			-	12142-12203 (62)	12255-12317 (63)	12147-12209 (63)	12298-12359 (62)
lrRNA	-				-	12203-13404 (1202)	12318-13509 (1192)	12210-13418 (1209)	12360-13575 (1216)
trnV	-	TAC			-	13405-13465 (61)	13510-13582 (73)	13419-13479 (61)	13576-13638 (63)
srRNA	-				-	13467-14191 (725)	13583-14293 (711)	13480-14279 (800)	13639-14374 (736)
A+T–rich region					-	14191-14755 (565)	14294-15287 (994)	14280-15034 (755)	14375-15698 (1324)

Gene	Strand	Anticodon	Start codon	Stop codon	*Ricania speculum*	*Ugyops* sp.
trnI	+	GAT			1-64 (64)	1-64 (64)
trnQ	-	TTG			69-137 (69)	65-131 (67)
trnM	+	CAT			152-216 (65)	130-193 (64)
ND2	+		ATT[a]/ATA[b]	TAA[1]/TAG[2]	217-1182 (966)[a, 1]	194-1153 (960)[a, 1]
trnW	+	TCA			1209-1272 (64)	1157-1219 (63)
trnC	-	GCA			1265-1327 (63)	1212-1272 (61)
trnY	-	GTA			1332-1393 (62)	1274-1334 (61)
COI	+		ATC[a]/ATG[b]/ATA[c]/CTG[d]	TAA[1]/T[2]	1398-2933 (1536)[b, 1]	1333-2866 (1534)[d, 2]
trnL$_2$	+	TAA			2935-2997 (63)	2867-2929 (63)
COII	+		ATA[a]/ATG[b]/ATT[c]	TAA[1]/T[2]/TAG[3]	2998-3679 (682)[c, 2]	2930-3601 (672)[a, 1]
trnK	+	CTT			3680-3749 (70)	3603-3674 (72)
trnD	+	GTC			3752-3815 (64)	3675-3736 (62)
ATP8	+		ATT[a]/ATA[b]/ATC[c]	TAA[1]/TAG[2]	3816-3977 (162)[b, 1]	3737-3844 (108)[b, 1]
ATP6	+		ATG[a]/ATA[b]/ATT[c]	T[1]/TAG[2]	3974-4622 (649)[b, 1]	3841-4492 (652)[b, 1]
COIII	+		ATG[a]/ATT[b]	TAG[1]/T[2]/TAA[3]	4623-5405 (783)[a, 1]	4493-5273 (781)[a, 2]
trnG	+	TCC			5409-5469 (61)	5274-5333 (60)
ND3	+		ATG[a]/ATT[b]/ATC[c] /ATA[d]	TAA[1]/TA[2]/TAG[3] /T[4]	5470-5817 (348)[d, 1]	5334-5684 (351)[b, 3]
trnA	+	TGC			5823-5887 (65)	5683-5743 (61)
trnR	+	TCG			5893-5954 (62)	5750-5809 (60)
trnN	+	GTT			5957-6024 (68)	5808-5871 (64)
trnS$_1$	+	GCT			6024-6082 (59)	5871-5931 (61)
trnE	+	TTC			6084-6148 (65)	5931-5996 (66)
trnF	-	GAA			6156-6220 (65)	5995-6058 (64)
ND5	-		ATG[a]/ATT[b]/TTG[c] /ATA[d]/GTG[e]	TAA[1]/TAG[2]/T[3]	6226-7890 (1665)[a, 1]	6059-7739 (1681)[e, 3]
trnH	-	GTG			7906-7969 (64)	7740-7803 (64)
ND4	-		ATG[a]/ATT[b]/ATA[c]	TAG[1]/T[2]/TAA[3]/TA[4]	7970-9296 (1327)[a, 2]	7804-9121 (1318)[a, 2]
ND4L	-		ATG[a]/ATT[b]	TAA[1]/TAG[2]	9290-9559 (270)[a, 1]	9115-9387 (273)[a, 1]
trnT	+	TGT			9562-9626 (65)	9389-9451 (63)
trnP	-	TGG			9638-9702 (65)	9451-9514 (64)
ND6	+		ATA[a]/ATT[b]/ATC[c]	TAA[1]/T[2]	9704-10198 (495)[c, 1]	9516-10008 (493)[b, 2]
CytB	+		ATG[a]/ATA[b]	TAA[1]/TAG[2]/T[3]	10191-11321 (1131)[a, 1]	10009-11130 (1122)[a, 1]
trnS$_2$	+	TGA			11321-11386 (66)	11130-11191 (62)
ND1	-		ATG[a]/GTG[b]	TAA[1]/T[2]/TA[3] /TAG[4]	11380-12318 (939)[a, 1]	11208-12123 (916)[a, 2]
trnL$_1$	-	TAG			12320-12383 (64)	12125-12186 (62)
lrRNA	-				12407-13604 (1198)	12187-13392 (1206)
trnV	-	TAC			13598-13659 (62)	13393-13461 (69)
srRNA	-				13657-14383 (727)	13462-14228 (767)
A+T–rich region					14384-15729 (1346)	14229-15259 (1031)

Gene	Strand	Anticodon	Start codon	Stop codon	*Saccharosydne procerus*	*Changeondelphax velitchkovskyi*	*Nilaparvata bakeri* (unknown)	*Nilaparvata lugens* (biotype Y, Zhejiang, China)	*Nilaparvata lugens* (biotype L, Zhejiang, China)
trnI	+	GAT			1-65 (65)	1-66 (66)	1-70 (70)	1-69 (69)	1-69 (69)
trnQ	-	TTG			67-132 (66)	72-137 (66)	74-139 (66)	73-139 (67)	73-139 (67)
trnM	+	CAT			132-195 (64)	137-198 (62)	139-202 (64)	139-202 (64)	139-202 (64)
ND2	+		ATT[a]/ATA[b]	TAA[1]/TAG[2]	196-1155 (960)[a, 1]	199-1152 (954)[a, 1]	203-1156 (954)[a, 1]	203-1156 (954)[a, 1]	203-1156 (954)[a, 1]
trnC	-	GCA			1154-1214 (61)	1156-1218 (63)	1157-1218 (62)	1157-1219 (63)	1157-1219 (63)
trnW	+	TCA			1223-1287 (65)	1247-1311 (65)	1231-1286 (56)	1228-1283 (56)	1227-1282 (56)
trnY	-	GTA			1302-1362 (61)	1327-1386 (60)	1307-1369 (63)	1304-1366 (63)	1303-1365 (63)
COI	+		ATC[a]/ATG[b]/ATA[c]/CTG[d]	TAA[1]/T[2]	1368-2901 (1534)[b, 2]	1395-2928 (1534)[b, 2]	1384-2922 (1539)[b, 1]	1380-2918 (1539)[b, 1]	1379-2917 (1539)[b, 1]
$trnL_2$	+	TAA			2902-2965 (64)	2929-2994 (66)	2918-2983 (66)	2914-2979 (66)	2913-2978 (66)
COII	+		ATA[a]/ATG[b]/ATT[c]	TAA[1]/T[2]/TAG[3]	2966-3631 (666)[c, 1]	2995-3657 (663)[c, 1]	2984-3646 (663)[c, 1]	2980-3642 (663)[c, 1]	2979-3641 (663)[c, 1]
trnK	+	CTT			3634-3703 (70)	3661-3731 (71)	3653-3722 (70)	3654-3724 (71)	3653-3723 (71)
trnD	+	GTC			3704-3763 (60)	3733-3794 (62)	3723-3786 (64)	3725-3788 (64)	3724-3787 (64)
ATP8	+		ATT[a]/ATA[b]/ATC[c]	TAA[1]/TAG[2]	3764-3865 (102)[a, 1]	3795-3896 (102)[a, 1]	3787-3888 (102)[b, 1]	3789-3887 (99)[a, 1]	3788-3886 (99)[a, 1]
ATP6	+		ATG[a]/ATA[b]/ATT[c]	T[1]/TAG[2]	3859-4513 (655)[a, 1]	3890-4544 (655)[b, 1]	3867-4518 (652)[c, 1]	3869-4520 (652)[c, 1]	3868-4519 (652)[c, 1]
COIII	+		ATG[a]/ATT[b]	TAG[1]/T[2]/TAA[3]	4514-5294 (781)[a, 2]	4545-5325 (781)[a, 2]	4519-5314 (796)[b, 2]	4521-5316 (796)[b, 2]	4520-5315 (796)[b, 2]
trnG	+	TCC			5295-5355 (61)	5326-5386 (61)	5315-5376 (62)	5317-5378 (62)	5316-5377 (62)
ND3	+		ATG[a]/ATT[b]/ATC[c]/ATA[d]	TAA[1]/TA[2]/TAG[3]/T[4]	5356-5706 (351)[b, 1]	5387-5737 (351)[b, 3]	5478-5723 (246)[c, 1]	5480-5725 (246)[c, 1]	5479-5724 (246)[c, 1]
trnA	+	TGC			5712-5774 (63)	5736-5796 (61)	5732-5792 (61)	5731-5791 (61)	5730-5790 (61)
trnR	+	TCG			5775-5833 (59)	5801-5861 (61)	5797-5858 (62)	5796-5855 (60)	5795-5854 (60)
trnN	+	GTT			5835-5897 (63)	5865-5929 (65)	5858-5921 (64)	5855-5918 (64)	5854-5917 (64)
$trnS_1$	+	GCT			5897-5954 (58)	5929-5987 (59)	5921-5980 (60)	5918-5976 (59)	5917-5975 (59)
trnE	+	TTC			5954-6015 (62)	5987-6046 (60)	5980-6042 (63)	5976-6039 (64)	5975-6039 (65)
trnF	-	GAA			6020-6086 (67)	6047-6110 (64)	6043-6108 (66)	6040-6105 (66)	6040-6105 (66)
ND5	-		ATG[a]/ATT[b]/TTG[c]/ATA[d]/GTG[e]	TAA[1]/TAG[2]/T[3]	6087-7758 (1672)[a, 3]	6111-7782 (1672)[e, 3]	6109-7783 (1675)[a, 3]	6106-7780 (1675)[a, 3]	6106-7780 (1675)[a, 3]
trnH	-	GTG			7759-7822 (64)	7783-7842 (60)	7783-7842 (60)	7780-7840 (61)	7780-7840 (61)
ND4	-		ATG[a]/ATT[b]/ATA[c]	TAG[1]/T[2]/TAA[3]/TA[4]	7826-9142 (1317)[a, 3]	7843-9160 (1318)[a, 2]	7843-9162 (1320)[b, 3]	7841-9160 (1320)[b, 3]	7841-9160 (1320)[b, 3]
ND4L	-		ATG[a]/ATT[b]	TAA[1]/TAG[2]	9136-9408 (273)[a, 2]	9154-9426 (273)[a, 1]	9153-9422 (270)[a, 1]	9151-9420 (270)[a, 1]	9151-9420 (270)[a, 1]
ND6	+		ATA[a]/ATT[b]/ATC[c]	TAA[1]/T[2]	9458-9964 (507)[a, 1]	9472-9996 (525)[b, 1]	9457-9996 (540)[b, 1]	9454-9993 (540)[b, 1]	9454-9993 (540)[b, 1]
trnP	-	TGG			10029-10092 (64)	10143-10205 (63)	10067-10128 (62)	10042-10103 (62)	10042-10103 (62)
trnT	+	TGT			10094-10157 (64)	10206-10267 (62)	10129-10194 (66)	10104-10165 (62)	10104-10166 (63)
CytB	+		ATG[a]/ATA[b]	TAA[1]/TAG[2]/T[3]	10162-11262 (1101)[a, 1]	10273-11376 (1104)[a, 1]	10203-11306 (1104)[a, 2]	10173-11276 (1104)[a, 2]	10174-11277 (1104)[a, 2]
$trnS_2$	+	TGA			11264-11325 (62)	11379-11436 (58)	11297-11358 (62)	11267-11328 (62)	11268-11329 (62)
ND1	-		ATG[a]/GTG[b]	TAA[1]/T[2]/TA[3]/TAG[4]	11341-12256 (916)[a, 2]	11654-12571 (918)[a, 1]	11379-12296 (918)[a, 4]	11349-12266 (918)[a, 4]	11350-12267 (918)[a, 4]
$trnL_1$	-	TAG			12258-12328 (71)	12573-12634 (62)	12298-12359 (62)	12268-12329 (62)	12269-12330 (62)
lrRNA	-				12329-13545 (1217)	12635-13847 (1213)	12360-13574 (1215)	12330-13548 (1219)	12331-13549 (1219)
trnV	-	TAC			13546-13615 (70)	13848-13916 (69)	13575-13636 (62)	13549-13610 (62)	13550-13611 (62)
srRNA	-				13616-14369 (754)	13917-14668 (752)	13641-14394 (754)	13615-14365 (751)	13616-14366 (751)
A+T–rich region					14370-16031 (1662)	14669-16449 (1781)	-	-	-

Gene	Strand	Anticodon	Start codon	Stop codon	*Nilaparvata lugens* (Hainan, China)	*Nilaparvata lugens* (Hadong-gun, Korea)[‡]	*Nilaparvata lugens* (Guangdong, China)	*Nilaparvata lugens* (biotype 1, Zhejiang, China)	*Nilaparvata lugens* (biotype 2, Zhejiang, China)
trnI	+	GAT			1-69 (69)	1-65 (65)	1-69 (69)	1-69 (69)	1-69 (69)
trnQ	-	TTG			73-139 (67)	63-131 (69)	73-139 (67)	73-139 (67)	73-139 (67)
trnM	+	CAT			139-202 (64)	131-199 (69)	139-202 (64)	139-202 (64)	139-202 (64)
ND2	+		ATT[a]/ATA[b]	TAA[1]/TAG[2]	203-1156 (954)[a, 1]	200-1213 (1014)[a, 1]	203-1156 (954)[a, 1]	203-1156 (954)[a, 1]	203-1156 (954)[a, 1]
trnC	-	GCA			1157-1219 (63), 1568-1630 (63), 1979-2041 (63)	1157-1219 (63), 1569-1631 (63), 1978-2040 (63)	1157-1219 (63), 1565-1627 (63), 1974-2036 (63)	1157-1219 (63)	1157-1219 (63)
trnW	+	TCA			2052-2116 (65)	2052-2116 (65)	2047-2111 (65)	1228-1283 (56)	1228-1283 (56)
trnY	-	GTA			2126-2188 (63)	2125-2187 (63)	2121-2183 (63)	1304-1366 (63)	1304-1366 (63)
COI	+		ATC[a]/ATG[b]/ATA[c]/CTG[d]	TAA[1]/T[2]	2202-3735 (1534)[b, 2]	2201-3734 (1534)[b, 2]	2197-3730 (1534)[b, 2]	1380-2918 (1539)[b, 1]	1380-2918 (1539)[b, 1]
$trnL_2$	+	TAA			3736-3801 (66)	3735-3800 (66)	3731-3796 (66)	2914-2979 (66)	2914-2979 (66)
COII	+		ATA[a]/ATG[b]/ATT[c]	TAA[1]/T[2]/TAG[3]	3802-4464 (663)[c, 1]	3801-4463 (663)[c, 1]	3797-4459 (663)[c, 1]	2980-3642 (663)[c, 1]	2980-3642 (663)[c, 1]
trnK	+	CTT			4476-4546 (71)	4475-4545 (71)	4471-4541 (71)	3654-3724 (71)	3654-3724 (71)
trnD	+	GTC			4547-4610 (64)	4546-4609 (64)	4542-4605 (64)	3725-3788 (64)	3725-3788 (64)
ATP8	+		ATT[a]/ATA[b]/ATC[c]	TAA[1]/TAG[2]	4611-4709 (99)[a, 1]	4610-4708 (99)[a, 1]	4606-4704 (99)[a, 1]	3789-3887 (99)[a, 1]	3789-3887 (99)[a, 1]
ATP6	+		ATG[a]/ATA[b]/ATT[c]	T[1]/TAG[2]	4709-5360 (652)[b, 1]	4708-5359 (652)[b, 1]	4704-5355 (652)[b, 1]	3869-4520 (652)[c, 1]	3869-4520 (652)[c, 1]
COIII	+		ATG[a]/ATT[b]	TAG[1]/T[2]/TAA[3]	5361-6138 (778)[a, 2]	5360-6137 (778)[a, 2]	5356-6133 (778)[a, 2]	4521-5316 (796)[b, 2]	4521-5316 (796)[b, 2]
trnG	+	TCC			6139-6200 (62)	6138-6199 (62)	6134-6195 (62)	5317-5378 (62)	5317-5378 (62)
ND3	+		ATG[a]/ATT[b]/ATC[c] /ATA[d]	TAA[1]/TA[2]/TAG[3] /T[4]	6201-6551 (351)[d, 1]	6200-6550 (351)[d, 1]	6196-6546 (351)[d, 1]	5480-5725 (246)[c, 1]	5480-5725 (246)[c, 1]
trnA	+	TGC			6553-6613 (61)	6552-6612 (61)	6548-6608 (61)	5731-5791 (61)	5731-5791 (61)
trnR	+	TCG			6618-6677 (60)	6617-6676 (60)	6613-6672 (60)	5796-5855 (60)	5796-5855 (60)
trnN	+	GTT			6677-6740 (64)	6676-6739 (64)	6672-6735 (64)	5855-5918 (64)	5855-5918 (64)
$trnS_1$	+	GCT			6741-6797 (57)	6740-6796 (57)	6736-6792 (57)	5918-5976 (59)	5918-5976 (59)
trnE	+	TTC			6798-6862 (65)	6797-6862 (66)	6793-6858 (66)	5976-6039 (64)	5976-6040 (65)
trnF	-	GAA			6863-6928 (66)	6863-6928 (66)	6859-6924 (66)	6040-6105 (66)	6041-6106 (66)
ND5	-		ATG[a]/ATT[b]/TTG[c] /ATA[d]/GTG[e]	TAA[1]/TAG[2]/T[3]	6929-8603 (1675)[a, 3]	6929-8603 (1675)[a, 3]	6925-8599 (1675)[a, 3]	6106-7783 (1678)[d, 3]	6107-7784 (1678)[d, 3]
trnH	-	GTG			8604-8664 (61)	8604-8664 (61)	8600-8660 (61)	7780-7840 (61)	7781-7841 (61)
ND4	-		ATG[a]/ATT[b]/ATA[c]	TAG[1]/T[2]/TAA[3]/TA[4]	8665-9980 (1316)[a, 4]	8665-9980 (1316)[a, 4]	8660-9976 (1317)[a, 3]	7841-9160 (1320)[b, 3]	7842-9161 (1320)[b, 3]
ND4L	-		ATG[a]/ATT[b]	TAA[1]/TAG[2]	9974-10243 (270)[a, 1]	9974-10243 (270)[a, 1]	9970-10239 (270)[a, 1]	9151-9420 (270)[a, 1]	9152-9421 (270)[a, 1]
ND6	+		ATA[a]/ATT[b]/ATC[c]	TAA[1]/T[2]	10277-10816 (540)[b, 1]	10277-10816 (9790)[b, 1]	10273-10812 (9786)[b, 1]	9454-9993 (540)[b, 1]	9455-9994 (540)[b, 1]
trnP	-	TGG			10865-10926 (62)	10865-10926 (62)	10861-10922 (62)	10041-10102 (62)	10043-10104 (62)
trnT	+	TGT			10927-10990 (64)	10927-10988 (62)	10923-10985 (63)	10103-10164 (62)	10105-10167 (63)
CytB	+		ATG[a]/ATA[b]	TAA[1]/TAG[2]/T[3]	10998-12101 (1104)[a, 2]	10996-12099 (1104)[a, 2]	10993-12096 (1104)[a, 2]	10172-11275 (1104)[a, 2]	10175-11278 (1104)[a, 2]
$trnS_2$	+	TGA			12092-12153 (62)	12090-12151 (62)	12087-12148 (62)	11266-11327 (62)	11269-11330 (62)
ND1	-		ATG[a]/GTG[b]	TAA[1]/T[2]/TA[3] /TAG[4]	12174-13091 (918)[a, 4]	12137-13087 (951)[a, 1]	12169-13083 (915)[a, 4]	11348-12265 (918)[a, 4]	11351-12268 (918)[a, 4]
$trnL_1$	-	TAG			13093-13154 (62)	13089-13150 (62)	13085-13146 (62)	12267-12328 (62)	12270-12331 (62)
lrRNA	-				13155-14373 (1219)	13151-14369 (1219)	13147-14365 (1219)	12329-13547 (1219)	12332-13550 (1219)
trnV	-	TAC			14373-14443 (71)	14369-14439 (71)	14365-14435 (71)	13548-13609 (62)	13551-13612 (62)
srRNA	-				14443-15190 (748)	14439-15186 (748)	14435-15182 (748)	13614-14364 (751)	13617-14367 (751)
A+T–rich region					15191-17619 (2429)	15187-17610 (2424)	15183-17606 (2424)	-	-

Gene	Strand	Anticodon	Start codon	Stop codon	*Nilaparvata lugens* (biotype 3, Zhejiang, China)	*Nilaparvata muiri*	*Nilaparvata* sp.[‡]	*Peregrinus maidis*	*Sogatella furcifera* (Hainan, China)
trnI	+	GAT			1-69 (69)	1-70 (70)	1-67 (67)	1-66 (66)	1-66 (66)
trnQ	-	TTG			73-139 (67)	73-138 (66)	84-149 (66)	83-148 (66)	69-134 (66)
trnM	+	CAT			139-202 (64)	138-200 (63)	149-213 (65)	148-209 (62)	134-195 (62)
ND2	+		ATT[a]/ATA[b]	TAA[1]/TAG[2]	203-1156 (954)[a, 1]	201-1154 (954)[a, 1]	214-1170 (957)[a, 2]	210-1166 (957)[a, 1]	196-1152 (957)[a, 1]
trnC	-	GCA			1157-1219 (63)	1155-1216 (62)	1169-1229 (61)	1165-1225 (61)	1151-1215 (65)
trnW	+	TCA			1228-1283 (56)	1224-1281 (58)	1249-1314 (66)	1266-1331 (66)	1234-1300 (67)
trnY	-	GTA			1304-1366 (63)	1302-1364 (63)	1318-1378 (61)	1333-1394 (62)	1311-1373 (63)
COI	+		ATC[a]/ATG[b]/ATA[c]/CTG[d]	TAA[1]/T[2]	1380-2918 (1539)[b, 1]	1379-2917 (1539)[b, 1]	1388-2921 (1534)[b, 2]	1403-2936 (1534)[b, 2]	1375-2908 (1534)[b, 2]
$trnL_2$	+	TAA			2914-2979 (66)	2913-2978 (66)	2922-2986 (65)	2937-3001 (65)	2909-2973 (65)
COII	+		ATA[a]/ATG[b]/ATT[c]	TAA[1]/T[2]/TAG[3]	2980-3642 (663)[c, 1]	2979-3641 (663)[c, 1]	2987-3649 (663)[a, 1]	3002-3664 (663)[a, 1]	2974-3637 (664)[c, 2]
trnK	+	CTT			3654-3724 (71)	3648-3717 (70)	3651-3722 (72)	3666-3736 (71)	3638-3708 (71)
trnD	+	GTC			3725-3788 (64)	3718-3781 (64)	3723-3784 (62)	3737-3801 (65)	3709-3768 (60)
ATP8	+		ATT[a]/ATA[b]/ATC[c]	TAA[1]/TAG[2]	3789-3887 (99)[a, 1]	3782-3880 (99)[c, 1]	3786-3887 (102)[b, 1]	3802-3900 (99)[b, 1]	3771-3872 (102)[a, 1]
ATP6	+		ATG[a]/ATA[b]/ATT[c]	T[1]/TAG[2]	3869-4520 (652)[c, 1]	3862-4513 (652)[c, 1]	3881-4535 (655)[c, 1]	3897-4551 (655)[b, 1]	3866-4520 (655)[a, 1]
COIII	+		ATG[a]/ATT[b]	TAG[1]/T[2]/TAA[3]	4521-5316 (796)[b, 2]	4514-5309 (796)[b, 2]	4536-5316 (781)[a, 2]	4552-5332 (781)[a, 2]	4521-5301 (781)[a, 2]
trnG	+	TCC			5317-5378 (62)	5310-5371 (62)	5318-5376 (59)	5333-5392 (60)	5302-5363 (62)
ND3	+		ATG[a]/ATT[b]/ATC[c] /ATA[d]	TAA[1]/TA[2]/TAG[3] /T[4]	5480-5725 (246)[c, 1]	5473-5718 (246)[c, 1]	5377-5727 (351)[d, 3]	5393-5743 (351)[b, 1]	5364-5712 (349)[b, 4]
trnA	+	TGC			5731-5791 (61)	5726-5786 (61)	5726-5788 (63)	5745-5805 (61)	5713-5773 (61)
trnR	+	TCG			5796-5855 (60)	5791-5850 (60)	5793-5853 (61)	5810-5870 (61)	5778-5838 (61)
trnN	+	GTT			5855-5918 (64)	5850-5913 (64)	5853-5916 (64)	5870-5933 (64)	5838-5900 (63)
$trnS_1$	+	GCT			5918-5976 (59)	5913-5972 (60)	5917-5975 (59)	5933-5992 (60)	5901-5954 (54)
trnE	+	TTC			5976-6040 (65)	5972-6037 (66)	5975-6039 (65)	5992-6054 (63)	5955-6017 (63)
trnF	-	GAA			6041-6106 (66)	6038-6103 (66)	6041-6103 (63)	6054-6116 (63)	6018-6085 (68)
ND5	-		ATG[a]/ATT[b]/TTG[c] /ATA[d]/GTG[e]	TAA[1]/TAG[2]/T[3]	6107-7784 (1678)[d, 3]	6104-7781 (1678)[d, 3]	6103-7785 (1683)[d, 1]	6117-7797 (1681)[d, 3]	6086-7760 (1675)[e, 3]
trnH	-	GTG			7782-7842 (61)	7779-7839 (61)	7788-7843 (56)	7800-7854 (55)	7761-7820 (60)
ND4	-		ATG[a]/ATT[b]/ATA[c]	TAG[1]/T[2]/TAA[3]/TA[4]	7842-9161 (1320)[b, 3]	7839-9158 (1320)[b, 3]	7845-9143 (1299)[c, 3]	7856-9172 (1317)[a, 3]	7821-9143 (1323)[a, 3]
ND4L	-		ATG[a]/ATT[b]	TAA[1]/TAG[2]	9152-9421 (270)[a, 1]	9149-9418 (270)[a, 1]	9155-9427 (273)[a, 1]	9166-9438 (273)[a, 2]	9137-9409 (273)[a, 1]
ND6	+		ATA[a]/ATT[b]/ATC[c]	TAA[1]/T[2]	9455-9994 (540)[b, 1]	9453-9992 (540)[b, 1]	9467-10006 (540)[a, 1]	9492-10010 (519)[a, 1]	9459-9968 (510)[b, 1]
trnP	-	TGG			10043-10104 (62)	10048-10109 (62)	10226-10287 (62)	10349-10410 (62)	10062-10123 (62)
trnT	+	TGT			10105-10167 (63)	10110-10170 (61)	10289-10351 (63)	10412-10475 (64)	10127-10189 (63)
CytB	+		ATG[a]/ATA[b]	TAA[1]/TAG[2]/T[3]	10175-11278 (1104)[a, 2]	10181-11284 (1104)[a, 2]	10356-11459 (1104)[a, 1]	10480-11583 (1104)[a, 1]	10194-11295 (1102)[a, 3]
$trnS_2$	+	TGA			11269-11330 (62)	11273-11334 (62)	11459-11516 (58)	11583-11638 (56)	11296-11351 (56)
ND1	-		ATG[a]/GTG[b]	TAA[1]/T[2]/TA[3] /TAG[4]	11351-12268 (918)[a, 4]	11357-12274 (918)[a, 4]	11534-12451 (918)[a, 1]	11656-12573 (918)[a, 4]	11369-12286 (918)[a, 4]
$trnL_1$	-	TAG			12270-12331 (62)	12274-12335 (62)	12453-12518 (66)	12575-12640 (66)	12288-12349 (62)
lrRNA	-				12332-13550 (1219)	12336-13554 (1219)	12519-13730 (1212)	12641-13862 (1222)	12350-13573 (1224)
trnV	-	TAC			13551-13612 (62)	13555-13616 (62)	13731-13801 (71)	13863-13933 (71)	13574-13642 (69)
srRNA	-				13617-14367 (751)	13619-14371 (753)	13802-14550 (749)	13934-14683 (750)	13643-14389 (747)
A+T–rich region					-	-	14551-15274 (724)	14684-16279 (1596)	14390-16612 (2223)

Gene	Strand	Anticodon	Start codon	Stop codon	*Sogatella furcifera* (Yunnan, China)	*Sogatella vibix*	*Laodelphax striatellus* (Jiangsu, China)	*Laodelphax striatella* (Suwon, Korea)	*Laodelphax striatella* (Milyang, Korea)
trnI	+	GAT			1-66 (66)	1-68 (68)	1-66 (66)	1-66 (66)	1-66 (66)
trnQ	-	TTG			69-134 (66)	71-138 (68)	69-134 (66)	69-134 (66)	69-134 (66)
trnM	+	CAT			134-195 (62)	138-199 (62)	134-198 (65)	134-198 (65)	134-198 (65)
ND2	+		ATT[a]/ATA[b]	TAA[1]/TAG[2]	196-1152 (957)[a, 1]	200-1156 (957)[a, 1]	199-1155 (957)[a, 1]	199-1155 (957)[a, 1]	199-1155 (957)[a, 1]
trnC	-	GCA			1151-1215 (65)	1155-1219 (65)	1159-1220 (62)	1159-1220 (62)	1159-1220 (62)
trnW	+	TCA			1234-1300 (67)	1236-1300 (65)	1255-1321 (67)	1255-1321 (67)	1255-1321 (67)
trnY	-	GTA			1311-1373 (63)	1310-1371 (62)	1325-1385 (61)	1325-1385 (61)	1325-1385 (61)
COI	+		ATC[a]/ATG[b]/ATA[c]/CTG[d]	TAA[1]/T[2]	1375-2908 (1534)[b, 2]	1373-2906 (1534)[b, 2]	1390-2923 (1534)[b, 2]	1390-2923 (1534)[b, 2]	1390-2923 (1534)[b, 2]
trnL$_2$	+	TAA			2909-2973 (65)	2907-2971 (65)	2924-2989 (66)	2924-2989 (66)	2924-2989 (66)
COII	+		ATA[a]/ATG[b]/ATT[c]	TAA[1]/T[2]/TAG[3]	2974-3637 (664)[c, 2]	2972-3634 (663)[c, 3]	2990-3652 (663)[c, 3]	2990-3652 (663)[c, 3]	2990-3652 (663)[c, 3]
trnK	+	CTT			3638-3708 (71)	3636-3706 (71)	3653-3723 (71)	3653-3723 (71)	3653-3723 (71)
trnD	+	GTC			3709-3768 (60)	3707-3768 (62)	3724-3785 (62)	3724-3785 (62)	3724-3785 (62)
ATP8	+		ATT[a]/ATA[b]/ATC[c]	TAA[1]/TAG[2]	3771-3872 (102)[a, 1]	3769-3870 (102)[a, 1]	3786-3887 (102)[a, 2]	3786-3887 (102)[a, 1]	3786-3887 (102)[a, 1]
ATP6	+		ATG[a]/ATA[b]/ATT[c]	T[1]/TAG[2]	3866-4520 (655)[a, 1]	3864-4518 (655)[a, 1]	3881-4535 (655)[a, 1]	3881-4535 (655)[a, 1]	3881-4535 (655)[a, 1]
COIII	+		ATG[a]/ATT[b]	TAG[1]/T[2]/TAA[3]	4521-5301 (781)[a, 2]	4519-5299 (781)[a, 2]	4536-5316 (781)[a, 2]	4536-5316 (781)[a, 2]	4536-5316 (781)[a, 2]
trnG	+	TCC			5302-5363 (62)	5300-5359 (60)	5317-5377 (61)	5317-5377 (61)	5317-5377 (61)
ND3	+		ATG[a]/ATT[b]/ATC[c] /ATA[d]	TAA[1]/TA[2]/TAG[3] /T[4]	5364-5712 (349)[b, 4]	5360-5710 (351)[b, 1]	5378-5727 (350)[b, 2]	5378-5727 (350)[b, 2]	5378-5728 (351)[b, 1]
trnA	+	TGC			5713-5773 (61)	5710-5770 (61)	5728-5788 (61)	5728-5788 (61)	5728-5788 (61)
trnR	+	TCG			5778-5838 (61)	5775-5835 (61)	5793-5855 (63)	5793-5855 (63)	5793-5855 (63)
trnN	+	GTT			5838-5900 (63)	5835-5898 (64)	5855-5918 (64)	5855-5918 (64)	5855-5918 (64)
trnS$_1$	+	GCT			5901-5954 (54)	5898-5954 (57)	5905-5973 (69)	5905-5973 (69)	5905-5973 (69)
trnE	+	TTC			5955-6017 (63)	5954-6016 (63)	5974-6035 (62)	5974-6035 (62)	5974-6035 (62)
trnF	-	GAA			6018-6085 (68)	6017-6083 (67)	6035-6099 (65)	6035-6099 (65)	6035-6099 (65)
ND5	-		ATG[a]/ATT[b]/TTG[c] /ATA[d]/GTG[e]	TAA[1]/TAG[2]/T[3]	6086-7760 (1675)[c, 3]	6084-7758 (1675)[a, 3]	6100-7774 (1675)[a, 3]	6100-7774 (1675)[a, 3]	6100-7774 (1675)[a, 3]
trnH	-	GTG			7761-7820 (60)	7759-7819 (61)	7775-7836 (62)	7775-7836 (62)	7775-7836 (62)
ND4	-		ATG[a]/ATT[b]/ATA[c]	TAG[1]/T[2]/TAA[3]/TA[4]	7821-9143 (1323)[a, 3]	7820-9142 (1323)[a, 3]	7839-9155 (1317)[a, 3]	7839-9155 (1317)[a, 3]	7839-9155 (1317)[a, 3]
ND4L	-		ATG[a]/ATT[b]	TAA[1]/TAG[2]	9137-9409 (273)[a, 1]	9136-9408 (273)[a, 2]	9149-9421 (273)[a, 1]	9149-9421 (273)[a, 1]	9149-9421 (273)[a, 1]
ND6	+		ATA[a]/ATT[b]/ATC[c]	TAA[1]/T[2]	9459-9968 (510)[a, 1]	9458-9979 (522)[b, 1]	9467-10006 (540)[b, 1]	9467-9994 (528)[b, 1]	9467-9994 (528)[b, 1]
trnP	-	TGG			10063-10124 (62)	10055-10116 (62)	10065-10126 (62)	10063-10124 (62)	10063-10124 (62)
trnT	+	TGT			10128-10189 (62)	10119-10182 (64)	10131-10193 (63)	10129-10191 (63)	10129-10191 (63)
CytB	+		ATG[a]/ATA[b]	TAA[1]/TAG[2]/T[3]	10194-11295 (1102)[a, 3]	10187-11290 (1104)[a, 2]	10198-11299 (1102)[a, 3]	10196-11299 (1104)[a, 2]	10196-11299 (1104)[a, 2]
trnS$_2$	+	TGA			11296-11351 (56)	11289-11344 (56)	11300-11355 (56)	11298-11353 (56)	11298-11353 (56)
ND1	-		ATG[a]/GTG[b]	TAA[1]/T[2]/TA[3] /TAG[4]	11369-12286 (918)[a, 4]	11362-12279 (918)[a, 4]	11373-12290 (918)[a, 4]	11371-12288 (918)[a, 4]	11371-12288 (918)[a, 4]
trnL$_1$	-	TAG			12288-12349 (62)	12281-12342 (62)	12292-12355 (64)	12290-12353 (64)	12290-12353 (64)
lrRNA	-				12350-13574 (1225)	12343-13569 (1227)	12356-13574 (1219)	12354-13572 (1219)	12354-13572 (1219)
trnV	-	TAC			13575-13643 (69)	13570-13638 (69)	13575-13642 (68)	13573-13640 (68)	13573-13640 (68)
srRNA	-				13644-14390 (747)	13639-14387 (749)	13643-14389 (747)	13641-14387 (747)	13641-14387 (747)
A+T–rich region					14391-16654 (2264)	14388-16554 (2167)	14390-16431 (2042)	14388-16359 (1972)	14388-16359 (1972)

Gene	Strand	Anticodon	Start codon	Stop codon	*Laodelphax striatellus* A1 (China)	*Laodelphax striatellus* B1 (China)
trnI	+	GAT			1-66 (66)	1-66 (66)
trnQ	-	TTG			69-134 (66)	69-134 (66)
trnM	+	CAT			134-198 (65)	134-198 (65)
ND2	+		ATT[a]/ATA[b]	TAA[1]/TAG[2]	199-1155 (957)[a, 1]	199-1155 (957)[a, 1]
trnC	-	GCA			1159-1220 (62)	1159-1220 (62)
trnW	+	TCA			1255-1321 (67)	1255-1321 (67)
trnY	-	GTA			1325-1385 (61)	1325-1385 (61)
COI	+		ATC[a]/ATG[b]/ATA[c]/CTG[d]	TAA[1]/T[2]	1390-2923 (1534)[b, 2]	1390-2923 (1534)[b, 2]
trnL₂	+	TAA			2924-2989 (66)	2924-2989 (66)
COII	+		ATA[a]/ATG[b]/ATT[c]	TAA[1]/T[2]/TAG[3]	2990-3652 (663)[c, 3]	2990-3652 (663)[c, 3]
trnK	+	CTT			3653-3723 (71)	3653-3723 (71)
trnD	+	GTC			3724-3785 (62)	3724-3785 (62)
ATP8	+		ATT[a]/ATA[b]/ATC[c]	TAA[1]/TAG[2]	3786-3887 (102)[a, 1]	3786-3887 (102)[a, 1]
ATP6	+		ATG[a]/ATA[b]/ATT[c]	T[1]/TAG[2]	3881-4535 (655)[a, 1]	3881-4535 (655)[a, 1]
COIII	+		ATG[a]/ATT[b]	TAG[1]/T[2]/TAA[3]	4536-5316 (781)[a, 2]	4536-5316 (781)[a, 2]
trnG	+	TCC			5317-5377 (61)	5317-5377 (61)
ND3	+		ATG[a]/ATT[b]/ATC[c] /ATA[d]	TAA[1]/TA[2]/TAG[3] /T[4]	5378-5727 (350)[b, 2]	5378-5727 (350)[b, 2]
trnA	+	TGC			5728-5788 (61)	5728-5788 (61)
trnR	+	TCG			5793-5855 (63)	5793-5855 (63)
trnN	+	GTT			5855-5918 (64)	5855-5918 (64)
trnS₁	+	GCT			5905-5973 (69)	5905-5973 (69)
trnE	+	TTC			5974-6035 (62)	5974-6035 (62)
trnF	-	GAA			6035-6099 (65)	6035-6099 (65)
ND5	-		ATG[a]/ATT[b]/TTG[c] /ATA[d]/GTG[e]	TAA[1]/TAG[2]/T[3]	6100-7774 (1675)[a, 3]	6100-7774 (1675)[a, 3]
trnH	-	GTG			7775-7836 (62)	7775-7836 (62)
ND4	-		ATG[a]/ATT[b]/ATA[c]	TAG[1]/T[2]/TAA[3]/TA[4]	7839-9155 (1317)[a, 3]	7839-9155 (1317)[a, 3]
ND4L	-		ATG[a]/ATT[b]	TAA[1]/TAG[2]	9149-9421 (273)[a, 1]	9149-9421 (273)[a, 1]
ND6	+		ATA[a]/ATT[b]/ATC[c]	TAA[1]/T[2]	9467-10006 (540)[b, 1]	9467-10006 (540)[b, 1]
trnP	-	TGG			10065-10126 (62)	10062-10123 (62)
trnT	+	TGT			10131-10193 (63)	10128-10190 (63)
CytB	+		ATG[a]/ATA[b]	TAA[1]/TAG[2]/T[3]	10198-11299 (1102)[a, 3]	10195-11296 (1102)[a, 3]
trnS₂	+	TGA			11300-11355 (56)	11297-11352 (56)
ND1	-		ATG[a]/GTG[b]	TAA[1]/T[2]/TA[3] /TAG[4]	11373-12290 (918)[a, 4]	11370-12287 (918)[a, 4]
trnL₁	-	TAG			12292-12355 (64)	12289-12352 (64)
lrRNA	-				12356-13574 (1219)	12353-13571 (1219)
trnV	-	TAC			13575-13642 (68)	13572-13639 (68)
srRNA	-				13643-14389 (747)	13640-14386 (747)
A+T–rich region					14390-16428 (2039)	14387-16418 (2032)

Gene	Strand	Anticodon	Start codon	Stop codon	*Laodelphax striatellus* (Beijing, China)
trnI	+	GAT			1-66 (66)
trnQ	-	TTG			69-134 (66)
trnM	+	CAT			134-198 (65)
ND2	+		ATT[a]/ATA[b]	TAA[1]/TAG[2]	199-1176 (978)[a, 1]
trnC	-	GCA			1180-1241 (62)
trnW	+	TCA			1276-1343 (68)
trnY	-	GTA			1347-1407 (61)
COI	+		ATC[a]/ATG[b]/ATA[c]/CTG[d]	TAA[1]/T[2]	1412-2945 (1534)[b, 2]
$trnL_2$	+	TAA			2946-3011 (66)
COII	+		ATA[a]/ATG[b]/ATT[c]	TAA[1]/T[2]/TAG[3]	3012-3695 (684)[c, 1]
trnK	+	CTT			3676-3746 (71)
trnD	+	GTC			3747-3808 (62)
ATP8	+		ATT[a]/ATA[b]/ATC[c]	TAA[1]/TAG[2]	3809-3970 (162)[a, 1]
ATP6	+		ATG[a]/ATA[b]/ATT[c]	T[1]/TAG[2]	3964-4618 (655)[a, 1]
COIII	+		ATG[a]/ATT[b]	TAG[1]/T[2]/TAA[3]	4619-5399 (781)[a, 2]
trnG	+	TCC			5400-5460 (61)
ND3	+		ATG[a]/ATT[b]/ATC[c]/ATA[d]	TAA[1]/TA[2]/TAG[3]/T[4]	5461-5811 (351)[b, 1]
trnA	+	TGC			5811-5871 (61)
trnR	+	TCG			5876-5939 (64)
trnN	+	GTT			5939-6002 (64)
$trnS_1$	+	GCT			5999-6062 (64)
trnE	+	TTC			6057-6118 (62)
trnF	-	GAA			6118-6182 (65)
ND5	-		ATG[a]/ATT[b]/TTG[c]/ATA[d]/GTG[e]	TAA[1]/TAG[2]/T[3]	6183-7914 (1732)[b, 3]
ND4	-		ATG[a]/ATT[b]	TAG[1]/T[2]/TAA[3]/TA[4]	7901-9238 (1338)[a, 3]
ND4L	-		ATG[a]/ATT[b]	TAA[1]/TAG[2]	9232-9504 (273)[a, 1]
trnH	-	GTG			9577-9637 (61)
ND6	+		ATA[a]/ATT[b]/ATC[c]	TAA[1]/T[2]	9637-10147 (511)[a, 2]
trnP	-	TGG			10148-10209 (62)
trnT	+	TGT			10214-10276 (63)
CytB	+		ATG[a]/ATA[b]	TAA[1]/TAG[2]/T[3]	10281-11384 (1104)[a, 2]
$trnS_2$	+	TGA			11370-11440 (71)
ND1	-		ATG[a]/GTG[b]	TAA[1]/T[2]/TA[3]/TAG[4]	11421-12374 (954)[a, 1]
$trnL_1$	-	TAG			12376-12439 (64)
lrRNA	-				12440-13658 (1219)
trnV	-	TAC			13659-13726 (68)
srRNA	-				13727-14473 (747)
A+T–rich region					14474-16513 (2040)

Note: The gene abbreviations are as follows: *COI*, *COII*, and *COIII* refer to the cytochrome oxidase subunits; *CytB* refers to cytochrome b; *ND1-6* refers to NADH dehydrogenase components; *srRNA* and *lrRNA* refer to small and large subunit ribosomal RNA (rRNA) genes, respectively; tRNAs are denoted as one-letter symbols in accordance with the IUPAC-IUB single-letter amino acid codes, except those encoding leucine and serine, which are labeled $trnL_1$ for the CTN codon family, $trnL_2$ for the TTR codon family, $trnS_1$ for the AGN codon family, and $trnS_2$ for the TCN codon family. Superscripts indicate identical start and stop codons among Fulgoroidea species; +, genes encoded in major strand; -, genes encoded in minor strand. Values in parentheses indicate gene size (bp).

Superscripts indicate identical start and stop codons among Fulgoroidea species.
‡, partially or fully re-annotated in this study.
