# Supplementary material for: Complete Mitochondrial Genomes of Metcalfa pruinosa and Salurnis marginella (Hemiptera: Flatidae): Genomic Comparison and Phylogenetic Inference in Fulgoroidea
Source: Curr Issues Mol Biol. 2021 Sep 30;43(3):1391–418. doi: 10.3390/cimb43030099 (PMC8929015; doi:10.3390/cimb43030099)
Supplement: Supplementary file 1 [file cimb-43-00099-s001.zip › Sup. Table 4. Four frequent codons.pdf]

Table S4

Frequency of four most frequently used codons in 45 mitochondrial genomes of Fulgoroidea.

Taxon	GenBank accession no.	PCGs				Total
		TTT (F)	ATT (I)	ATA (I)	TTA (L)	
**Fulgoroidea**						
**Flatidae**						
Flatinae						
Metcalfa pruinosa (H1)	MK303326	11.52	9.00	8.64	6.46	35.61
Metcalfa pruinosa (H3)	MN417319	11.52	9.00	8.64	6.48	35.64
Salurnis marginella	MT628542	11.58	8.74	8.08	6.38	34.78
Geisha distinctissima	FJ230961	10.76	7.76	7.18	6.30	32.01
**Fulgoridae**						
Fulgorinae						
Pyrops candelaria	FJ006724	10.26	7.11	8.67	4.67	30.71
Aphaeninae						
Lycorma delicatula (Henan, China)	EU909203	10.74	7.53	8.46	6.32	33.04
Lycorma delicatula (China)	FJ456942	11.33	7.60	8.48	6.26	33.67
Lycorma delicatula (Korea)	MN607209	11.32	7.25	7.06	5.52	31.15
Aphaena (*Callidepsa*) *amabilis*	MN025522	12.17	8.34	8.89	7.30	36.70
Aphaena (*Aphaena*) *discolor nigrotibiata*	MN025523	12.12	8.24	8.87	6.60	35.82
**Achilidae**						
Achilinae						
Betatropis formosana	MH324927	11.83	8.33	8.41	7.35	35.92
Magadhaideus luodiana sp. nov	MH324928	10.25	7.45	7.29	5.15	30.14
Peltatavertexalis horizontalis sp. nov	MH324929	10.86	8.19	8.36	5.66	33.07
Plectoderini sp.	MH324930	11.00	7.76	7.76	5.82	32.35
Paracatonidia sp.	MH324931	10.95	8.01	8.56	6.72	34.24
**Cixiidae**						
Cixiinae						
Pentastiridius sp.	KY039133	9.59	9.74	7.19	7.96	34.47
**Derbidae**						
Otiocerinae						
Lydda sp.	KY039126	10.70	9.75	8.17	8.95	37.57
**Issidae**						
Issinae						
Sivaloka damnosus	FJ360694	11.70	8.72	7.42	6.73	34.57
Sivaloka sp.	KY039137	10.41	9.68	7.82	6.67	34.58
**Ricaniidae**						
Ricaniinae						
Ricania marginalis	JN242415	11.19	8.69	6.72	6.28	32.89
Ricania speculum	KX371891	11.76	8.27	6.68	5.66	32.36
**Delphacidae**						
Asiracinae						
Ugyops sp.	MH352481	11.46	7.45	9.50	6.48	34.88
Delphacinae						
Saccharosydnini						
Saccharosydne procerus	MG515237	10.55	11.16	6.94	11.80	40.45
Delphacini						
Changeondelphax velitchkovskyi	MG049916	9.35	8.37	5.82	9.90	33.44

Nilaparvata bakeri (unknown)	KC333655	10.02	9.66	6.50	9.55	35.73
Nilaparvata bakeri (biotype Y, Zhejiang, China)	KC333653	9.83	9.74	6.03	9.52	35.12
Nilaparvata bakeri (biotype L, Zhejiang, China)	KC333654	9.83	9.74	6.03	9.52	35.12
Nilaparvata lugens (Hainan, China)	JX880069	9.95	9.73	6.40	9.70	35.78
Nilaparvata lugens (Hadong-gun, Korea)	MK590088	10.11	9.70	6.38	9.84	36.03
Nilaparvata lugens (Guangdong, China)	MK606371	9.98	9.73	6.38	9.75	35.84
Nilaparvata lugens (biotype 1, Zhejiang, China)	JN563995	9.85	9.74	6.06	9.52	35.18
Nilaparvata lugens (biotype 2, Zhejiang, China)	JN563996	9.74	9.72	6.09	9.58	35.12
Nilaparvata lugens (biotype 3, Zhejiang, China)	JN563997	9.80	9.66	6.11	9.52	35.09
Nilaparvata muiri	JN563998	9.60	9.32	6.20	9.02	34.14
Nilaparvata sp.	KY039125	9.61	9.48	6.35	10.09	35.52
Peregrinus maidis	MG049917	9.32	8.95	6.04	10.78	35.10
Sogatella furcifera (Hainan, China)	KC512914	10.32	8.96	5.57	9.37	34.22
Sogatella furcifera (Yunnan, China)	KC512915	10.32	8.96	5.57	9.35	34.19
Sogatella vibix	MG515238	10.11	9.12	5.76	9.64	34.64
Laodelphax striatellus (Jiangsu, China)	JX880068	9.49	9.08	5.81	9.96	34.35
Laodelphax striatellus (Suwon, Korea)	MK838101	9.59	9.09	5.79	9.95	34.41
Laodelphax striatellus (Milyang, Korea)	MK862265	9.59	9.09	5.79	9.95	34.41
Laodelphax striatellus A1 (China)	MK292897	9.58	9.00	5.90	10.07	34.54
Laodelphax striatellus B1 (China)	MK292932	9.49	9.05	5.87	9.99	34.40
Laodelphax striatellus (Beijing, China)	FJ360695	9.63	8.92	5.71	10.07	34.33

Within parenthesis indicate corresponding amino acids.
