# Supplementary material for: Complete Mitochondrial Genomes of Metcalfa pruinosa and Salurnis marginella (Hemiptera: Flatidae): Genomic Comparison and Phylogenetic Inference in Fulgoroidea
Source: Curr Issues Mol Biol. 2021 Sep 30;43(3):1391–418. doi: 10.3390/cimb43030099 (PMC8929015; doi:10.3390/cimb43030099)
Supplement: Supplementary file 1 [file cimb-43-00099-s001.zip › Sup. Table 5. Size of tRNAs avaliable in Fulgoroidea.pdf]

Table S5
Size of mitochondrial tRNAs available in Fulgoroidea.

Taxon	*trnI*	*trnQ*	*trnM*	*trnW*	*trnC*	*trnY*	*trnL*$_2$	*trnK*	*trnD*	*trnG*	*trnA*	*trnR*	*trnN*	*trnS*$_1$	*trnE*	*trnF*	*trnH*	*trnT*	*trnP*	*trnS*$_2$	*trnL*$_1$	*trnV*	Average	References
**Fulgoroidea**																								
**Flatidae**																								
Flatinae																								
Metcalfa pruinosa (H1)	65	69	66	68	64	64	63	70	67	65	68	64	65	61	65	65	65	64	66	63	65	73	65.68	This study
Metcalfa pruinosa (H3)	65	69	66	68	64	64	63	70	67	65	68	64	65	61	65	65	65	64	66	63	65	73	65.68	This study
Salurnis marginella	62	69	66	65	60	61	62	69	63	61	63	61	63	59	62	63	62	61	63	61	62	70	63.09	This study
**Fulgoridae**																								
Fulgorinae																								
Aphaena (*Callidepsa*) *amabilis*	63	69	64	62	63	62	69	70	63	65	65	61	62	58	66	63	62	65	62	63	62	64	63.77	Wang et al. (2019)
Aphaena (*Aphaena*) *discolor nigrotibiata*	64	69	63	62	60	62	68	70	68	63	65	61	62	60	62	63	62	65	63	62	62	61	63.50	Wang et al. (2019)
**Achilidae**																								
Achilinae																								
Betatropis formosana	64	69	63	64	63	62	64	68	62	61	66	62	62	60	64	63	63	63	62	66	63	63	63.50	Xu et al. (2019)
Magadhaideus luodiana sp. nov	65	69	66	71	67	63	62	69	62	62	65	59	64	55	63	63	62	62	63	64	66	64	63.91	Xu et al. (2019)
Peltatavertexalis horizontalis sp. nov	64	69	65	63	64	62	62	70	63	65	63	60	63	56	64	67	62	64	63	64	63	64	63.64	Xu et al. (2019)
Plectoderini sp.	64	69	67	67	63	62	63	69	72	64	68	61	64	60	63	64	62	62	63	63	64	63	64.41	Xu et al. (2019)
Paracatonidia sp.	64	69	64	65	61	62	66	70	62	61	65	62	63	63	62	67	63	63	65	64	66	65	64.18	Xu et al. (2019)
**Issidae**																								
Issinae																								
Sivaloka damnosus	66	69	66	64	63	67	63	70	62	61	67	66	66	59	62	65	61	63	63	65	63	73	64.73	Song et al. (2010)
**Delphacidae**																								
Asiracinae																								
Ugyops sp.	64	67	64	63	61	61	63	72	62	60	61	60	64	61	66	62	64	63	64	62	62	69	63.41	Yu and Liang (2018)
Delphacinae																								
Sogatella furcifera (Hainan, China)	66	66	62	67	65	63	65	71	60	62	61	61	63	54	63	68	60	63	62	56	62	69	63.14	Zhang et al. (2014)
Sogatella furcifera (Yunnan, China)	66	66	62	67	65	63	65	71	60	62	61	61	63	54	63	68	60	62	62	56	62	69	63.09	Zhang et al. (2014)
Range	62-66	66-69	62-67	62-71	60-67	61-67	62-69	68-72	60-72	60-65	61-68	59-66	62-66	54-63	62-66	62-68	60-65	61-65	62-66	56-66	62-66	61-73		
