# Supplementary material for: Complete Mitochondrial Genomes of Metcalfa pruinosa and Salurnis marginella (Hemiptera: Flatidae): Genomic Comparison and Phylogenetic Inference in Fulgoroidea
Source: Curr Issues Mol Biol. 2021 Sep 30;43(3):1391–418. doi: 10.3390/cimb43030099 (PMC8929015; doi:10.3390/cimb43030099)
Supplement: Supplementary file 1 [file cimb-43-00099-s001.zip › Sup. Table 6. Skewness.pdf]

Table S6

Skewness of 45 mitochondrial genomes Fulgoroidea.

Taxon	Whole genome		Whole PCG		PCG-major strand		PCG-Minor strand	
	A/T	G/C	A/T	G/C	A/T	G/C	A/T	G/C
**Flatidae**								
Flatinae								
Metcalfa pruinosa (H1)	0.262	-0.274	-0.106	-0.062	0.149	-0.241	-0.483	0.289
Metcalfa pruinosa (H3)	0.261	-0.274	-0.106	-0.062	0.147	-0.241	-0.483	0.289
Salurnis marginella	0.242	-0.248	-0.119	-0.077	0.131	-0.236	-0.490	0.247
Geisha distinctissima	0.265	-0.269	-0.132	-0.049	0.125	-0.250	-0.528	0.328
**Fulgoridae**								
Fulgorinae								
Pyrops candelaria	0.284	-0.195	-0.106	-0.055	0.168	-0.210	-0.514	0.236
Aphaeninae								
Lycorma delicatula (Henan, China)	0.275	-0.280	-0.107	-0.080	0.161	-0.301	-0.508	0.336
Lycorma delicatula (China)	0.277	-0.283	-0.108	-0.085	0.161	-0.301	-0.512	0.327
Lycorma delicatula (Korea)	0.273	-0.277	-0.108	-0.085	0.163	-0.301	-0.512	0.327
Aphaena (Callidepsa) *amabilis*	0.277	-0.267	-0.117	-0.079	0.152	-0.269	-0.514	0.299
Aphaena (Aphaena) *discolor nigrotibiata*	0.299	-0.278	-0.110	-0.071	0.176	-0.274	-0.534	0.320
**Achilidae**								
Achilinae								
Betatropis formosana	0.265	-0.256	-0.108	-0.067	0.156	-0.279	-0.499	0.356
Magadhaideus luodiana sp. nov	0.199	-0.253	-0.131	-0.087	0.103	-0.289	-0.472	0.314
Peltatavertexalis horizontalis sp. nov	0.256	-0.273	-0.120	-0.058	0.124	-0.249	-0.488	0.313
Plectoderini sp.	0.258	-0.284	-0.113	-0.078	0.144	-0.302	-0.494	0.369
Paracatonidia sp.	0.234	-0.319	-0.118	-0.085	0.118	-0.326	-0.475	0.388
**Cixiidae**								
Cixiinae								
Pentastiridius sp.	0.183	-0.304	-0.106	-0.098	0.065	-0.289	-0.451	0.376
**Derbidae**								
Otiocerinae								
Lydda sp.	0.223	-0.280	-0.112	-0.088	0.100	-0.295	-0.442	0.313
**Issidae**								
Issinae								
Sivaloka damnosus	0.226	-0.243	-0.130	-0.049	0.091	-0.226	-0.465	0.294
Sivaloka sp.	0.230	-0.259	-0.120	-0.065	0.092	-0.255	-0.463	0.310
**Ricaniidae**								
Ricaniinae								
Ricania marginalis	0.236	-0.213	-0.120	-0.064	0.107	-0.203	-0.469	0.190
Ricania speculum	0.251	-0.218	-0.151	-0.055	0.099	-0.207	-0.530	0.233
**Delphacidae**								
Asiracinae								
Ugyops sp.	0.289	-0.268	-0.102	-0.068	0.190	-0.270	-0.541	0.327
Delphacinae								
Saccharosydnini								
Saccharosydne procerus	0.133	-0.221	-0.144	-0.067	-0.001	-0.222	-0.364	0.234
Delphacini								
Changeondelphax velitchkovskyi	0.131	-0.273	-0.152	-0.098	0.006	-0.299	-0.386	0.286
Nilaparvata bakeri (unknown)	0.106	-0.231	-0.149	-0.085	-0.027	-0.247	-0.329	0.224
Nilaparvata lugens (biotype Y, Zhejiang, China)	0.111	-0.227	-0.157	-0.071	-0.032	-0.231	-0.344	0.238
Nilaparvata lugens (biotype L, Zhejiang, China)	0.111	-0.224	-0.158	-0.067	-0.032	-0.223	-0.344	0.238
Nilaparvata lugens (Hainan, China)	0.091	-0.183	-0.155	-0.075	-0.031	-0.233	-0.344	0.238
Nilaparvata lugens (Hadong-gun, Korea)	0.089	-0.188	-0.155	-0.067	-0.031	-0.230	-0.340	0.240
Nilaparvata lugens (Guangdong, China)	0.089	-0.183	-0.156	-0.071	-0.032	-0.230	-0.344	0.238
Nilaparvata lugens (biotype 1, Zhejiang, China)	0.111	-0.227	-0.158	-0.071	-0.032	-0.228	-0.344	0.238
Nilaparvata lugens (biotype 2, Zhejiang, China)	0.111	-0.227	-0.157	-0.071	-0.030	-0.231	-0.342	0.238
Nilaparvata lugens (biotype 3, Zhejiang, China)	0.111	-0.222	-0.157	-0.071	-0.030	-0.231	-0.343	0.232
Nilaparvata muiri	0.110	-0.227	-0.151	-0.086	-0.026	-0.234	-0.334	0.213
Nilaparvata sp.	0.113	-0.247	-0.153	-0.082	-0.020	-0.251	-0.357	0.251
Peregrinus maidis	0.108	-0.243	-0.150	-0.083	-0.011	-0.254	-0.359	0.256
Sogatella furcifera (Hainan, China)	0.092	-0.143	-0.169	-0.067	-0.044	-0.170	-0.356	0.140
Sogatella furcifera (Yunnan, China)	0.094	-0.143	-0.169	-0.070	-0.043	-0.173	-0.356	0.140
Sogatella vibix	0.099	-0.158	-0.162	-0.073	-0.033	-0.182	-0.358	0.138
Laodelphax striatellus (Jiangsu, China)	0.119	-0.184	-0.144	-0.091	0.000	-0.245	-0.359	0.217
Laodelphax striatellus (Suwon, Korea)	0.120	-0.189	-0.145	-0.091	0.000	-0.242	-0.359	0.217
Laodelphax striatellus (Milyang, Korea)	0.120	-0.189	-0.145	-0.091	0.000	-0.242	-0.359	0.217
Laodelphax striatellus A1 (China)	0.119	-0.184	-0.145	-0.091	-0.001	-0.242	-0.357	0.217
Laodelphax striatellus B1 (China)	0.119	-0.184	-0.145	-0.091	0.000	-0.245	-0.359	0.217
Laodelphax striatellus (Beijing, China)	0.119	-0.184	-0.137	-0.083	0.007	-0.237	-0.346	0.217

PCG, protein-coding gene. Stop codon was excluded in the count.
