# Supplementary material for: Complete Mitochondrial Genomes of Metcalfa pruinosa and Salurnis marginella (Hemiptera: Flatidae): Genomic Comparison and Phylogenetic Inference in Fulgoroidea
Source: Curr Issues Mol Biol. 2021 Sep 30;43(3):1391–418. doi: 10.3390/cimb43030099 (PMC8929015; doi:10.3390/cimb43030099)
Supplement: Supplementary file 1 [file cimb-43-00099-s001.zip › Sup. Table 7. KaKs.pdf]

Table S7

Ka/Ks ratio of protein-coding genes in 45 mitochondrial genomes of Fulgoroidea.

Species	PCGs			Major-strand PCG			Minor-strand PCGs		
	Ka	Ks	Ka/Ks	Ka	Ks	Ka/Ks	Ka	Ks	Ka/Ks
**Fulgoroidea**									
**Flatidae**									
Flatinae									
Metcalfa pruinosa (H1)	0.4780	5.1564	0.0927	0.4405	5.7321	0.0769	0.5888	5.5307	0.1065
Metcalfa pruinosa (H3)	0.4787	5.1545	0.0929	0.4415	5.7285	0.0771	0.5879	5.5317	0.1063
Salurnis marginella	0.4811	5.1757	0.0929	0.4456	5.6546	0.0788	0.5793	5.6699	0.1022
Geisha distinctissima	0.4882	4.7852	0.1020	0.4413	5.4352	0.0812	0.6251	4.9619	0.1260
**Fulgoridae**									
Fulgorinae									
Pyrops candelaria	0.5001	4.4889	0.1114	0.4691	4.9236	0.0953	0.5806	4.9932	0.1163
Aphaeninae									
Lycorma delicatula (Henan, China)	0.4865	5.0366	0.0966	0.4455	5.6297	0.0791	0.5998	5.5967	0.1072
Lycorma delicatula (China)	0.4772	5.1504	0.0927	0.4416	5.6915	0.0776	0.5755	5.8545	0.0983
Lycorma delicatula (Korea)	0.4782	5.1384	0.0931	0.4423	5.7005	0.0776	0.5690	6.5022	0.0875
Aphaena (*Callidepsa*) *amabilis*	0.4860	5.3906	0.0902	0.4543	6.0451	0.0751	0.5870	6.0873	0.0964
Aphaena (*Aphaena*) *discolor nigrotibiata*	0.4849	5.1275	0.0946	0.4573	5.8834	0.0777	0.5805	5.6180	0.1033
**Achilidae**									
Achilinae									
Betatropis formosana	0.4800	5.2248	0.0919	0.4490	5.9000	0.0761	0.5654	4.7197	0.1198
Magadhaideus luodiana sp. nov	0.4885	4.4312	0.1103	0.4433	4.7532	0.0933	0.5748	4.8082	0.1195
Peltatavertexalis horizontalis sp. nov	0.4856	4.9169	0.0988	0.4437	5.3385	0.0831	0.5942	5.0915	0.1167
Plectoderini sp.	0.4858	4.8000	0.1012	0.4462	5.1524	0.0866	0.5865	5.2409	0.1119
Paracatonidia sp.	0.4685	5.1277	0.0914	0.4319	5.7053	0.0757	0.5542	5.2030	0.1065
**Cixiidae**									
Cixiinae									
Pentastiridius sp.	0.4789	5.1556	0.0929	0.4518	5.5447	0.0815	0.5599	5.1704	0.1083
**Derbidae**									
Otiocerinae									
Lydda sp.	0.4865	5.3193	0.0915	0.4515	5.9683	0.0756	0.5870	5.3815	0.1091
**Issidae**									
Issinae									
Sivaloka damnosus	0.5252	4.6808	0.1122	0.4719	5.3747	0.0878	0.6544	4.1794	0.1566
Sivaloka sp.	0.4556	5.3276	0.0855	0.4259	5.5910	0.0762	0.5626	6.0192	0.0935
**Ricaniidae**									
Ricaniinae									
Ricania marginalis	0.4987	4.8020	0.1038	0.4564	5.5054	0.0829	0.6028	4.4061	0.1368
Ricania speculum	0.4674	5.0202	0.0931	0.4278	5.4042	0.0792	0.5660	5.2673	0.1075
**Delphacidae**									
Asiracinae									
Ugyops sp.	0.4732	5.3467	0.0885	0.4481	6.1408	0.0730	0.5728	6.1580	0.0930
Delphacinae									
Saccharosydnini									
Saccharosydne procerus	0.4882	5.9126	0.0826	0.4502	6.3138	0.0713	0.5863	5.7587	0.1018
Delphacini									
Changeondelphax velitchkovskyi	0.5032	4.6081	0.1092	0.4560	4.8531	0.0939	0.5654	4.7197	0.1198
Nilaparvata bakeri (unknown)	0.4855	5.2052	0.0933	0.4466	5.2539	0.0850	0.5730	5.2730	0.1087
Nilaparvata lugens (biotype Y, Zhejiang, China)	0.4939	4.9521	0.0997	0.4526	5.0665	0.0893	0.5790	4.9300	0.1174
Nilaparvata lugens (biotype L, Zhejiang, China)	0.4944	4.9684	0.0995	0.4528	5.0990	0.0888	0.5808	4.9239	0.1180
Nilaparvata lugens (Hainan, China)	0.4903	5.0290	0.0975	0.4467	5.1829	0.0862	0.5796	4.9481	0.1171
Nilaparvata lugens (Hadong-gun, Korea)	0.4901	5.0926	0.0962	0.4458	5.2458	0.0850	0.5805	5.0023	0.1161
Nilaparvata lugens (Guangdong, China)	0.4901	5.0697	0.0967	0.4464	5.2249	0.0854	0.5795	4.9611	0.1168
Nilaparvata lugens (biotype 1, Zhejiang, China)	0.4937	4.9612	0.0995	0.4524	5.0719	0.0892	0.5789	4.9408	0.1172
Nilaparvata lugens (biotype 2, Zhejiang, China)	0.4942	4.9490	0.0999	0.4527	5.0712	0.0893	0.5795	4.9213	0.1178
Nilaparvata lugens (biotype 3, Zhejiang, China)	0.4939	4.9437	0.0999	0.4525	5.0661	0.0893	0.5793	4.9196	0.1178
Nilaparvata muiri	0.4892	4.8592	0.1007	0.4519	4.8506	0.0932	0.5664	5.0748	0.1116
Nilaparvata sp.	0.4953	5.0703	0.0977	0.4535	5.2758	0.0860	0.5887	5.1242	0.1149
Peregrinus maidis	0.4951	4.8830	0.1014	0.4531	5.1592	0.0878	0.5943	4.8054	0.1237
Sogatella furcifera (Hainan, China)	0.4981	4.9242	0.1012	0.4539	4.9761	0.0912	0.5820	5.1253	0.1136
Sogatella furcifera (Yunnan, China)	0.4982	4.9208	0.1012	0.4543	4.9664	0.0915	0.5806	5.1508	0.1127
Sogatella vibix	0.4936	5.1024	0.0967	0.4512	5.2516	0.0859	0.5904	5.2355	0.1128
Laodelphax striatellus (Jiangsu, China)	0.5023	5.0074	0.1003	0.4559	5.1709	0.0882	0.6017	5.1664	0.1165
Laodelphax striatella (Suwon, Korea)	0.5025	5.0221	0.1001	0.4568	5.1844	0.0881	0.5992	5.1809	0.1156
Laodelphax striatella (Milyang, Korea)	0.5025	5.0221	0.1001	0.4568	5.1844	0.0881	0.5992	5.1809	0.1156
Laodelphax striatellus A1 (China)	0.5040	5.0036	0.1007	0.4584	5.1657	0.0887	0.5998	5.1864	0.1157
Laodelphax striatellus B1 (China)	0.5040	4.9958	0.1009	0.4582	5.1596	0.0888	0.6005	5.1681	0.1162
Laodelphax striatellus (Beijing, China)	0.5082	4.9734	0.1022	0.4611	5.1952	0.0888	0.6165	4.9848	0.1237

Species	*ATP8*			*ATP6*			*COI*		
	Ka	Ks	Ka/Ks	Ka	Ks	Ka/Ks	Ka	Ks	Ka/Ks
**Fulgoroidea**									
**Flatidae**									
Flatinae									
Metcalfa pruinosa (H1)	0.8789	1.7498	0.5023	0.5453	4.2239	0.1291	0.2301	5.1599	0.0446
Metcalfa pruinosa (H3)	0.8789	1.7498	0.5023	0.5544	4.1406	0.1339	0.2301	5.1519	0.0447
Salurnis marginella	0.7011	2.9817	0.2352	0.5487	3.8436	0.1427	0.2248	4.8735	0.0461
Geisha distinctissima	0.9353	1.3676	0.6839	0.5114	4.2204	0.1212	0.2150	4.7884	0.0449
**Fulgoridae**									
Fulgorinae									
Pyrops candelaria	0.8672	2.0296	0.4273	0.5217	3.7945	0.1375	0.2266	4.5304	0.0500
Aphaeninae									
Lycorma delicatula (Henan, China)	0.8015	2.7124	0.2955	0.5125	4.5942	0.1115	0.2175	5.1387	0.0423
Lycorma delicatula (China)	0.8068	2.6297	0.3068	0.5078	4.6108	0.1101	0.2144	5.1156	0.0419
Lycorma delicatula (Korea)	0.8015	2.7124	0.2955	0.5132	4.5807	0.1120	0.2144	5.1205	0.0419
Aphaena (*Callidepsa*) *amabilis*	0.8276	2.5799	0.3208	0.5227	4.0185	0.1301	0.2200	5.2626	0.0418
Aphaena (*Aphaena*) *discolor nigrotibiata*	0.8130	2.8495	0.2853	0.5441	3.9249	0.1386	0.2215	5.1727	0.0428
**Achilidae**									
Achilinae									
Betatropis formosana	0.8889	1.9448	0.4571	0.5294	4.1372	0.1280	0.2194	5.1782	0.0424
Magadhaideus luodiana sp. nov	0.9384	1.4368	0.6531	0.5532	3.5620	0.1553	0.2154	4.7026	0.0458
Peltatavertexalis horizontalis sp. nov	0.9228	1.4273	0.6465	0.5896	3.4019	0.1733	0.2100	4.9360	0.0425
Plectoderini sp.	0.8304	2.3338	0.3558	0.5728	3.5138	0.1630	0.2240	4.7597	0.0471
Paracatonidia sp.	0.8600	2.0116	0.4275	0.5107	4.1834	0.1221	0.2087	5.1917	0.0402
**Cixiidae**									
Cixiinae									
Pentastiridius sp.	0.8155	2.1343	0.3821	0.5317	4.1018	0.1296	0.2044	5.3338	0.0383
**Derbidae**									
Otiocerinae									
Lydda sp.	0.7579	3.0301	0.2501	0.5674	3.9737	0.1428	0.2236	5.2817	0.0423
**Issidae**									
Issinae									
Sivaloka damnosus	0.7246	2.6240	0.2761	0.5848	3.6503	0.1602	0.2674	5.0122	0.0534
Sivaloka sp.	0.7267	2.4713	0.2941	0.5192	4.5070	0.1152	0.2180	5.1082	0.0427
**Ricaniidae**									
Ricaniinae									
Ricania marginalis	0.8037	2.5821	0.3113	0.5569	4.0062	0.1390	0.2258	4.8422	0.0466
Ricania speculum	0.8207	2.5655	0.3199	0.5495	4.0232	0.1366	0.2155	4.9293	0.0437
**Delphacidae**									
Asiracinae									
Ugyops sp.	0.7768	2.7516	0.2823	0.4680	4.6823	0.1000	0.2183	5.1886	0.0421
Delphacinae									
Saccharosydnini									
Saccharosydne procerus	0.7795	2.2144	0.3520	0.5376	4.5807	0.1174	0.2014	5.3935	0.0373
Delphacini									
Changeondelphax velitchkovskyi	0.8500	2.1680	0.3921	0.5797	3.4795	0.1666	0.2003	4.8812	0.0410
Nilaparvata bakeri (unknown)	0.7999	2.7394	0.2920	0.5941	3.8231	0.1554	0.1937	5.1913	0.0373
Nilaparvata lugens (biotype Y, Zhejiang, China)	0.7351	2.9447	0.2496	0.5974	3.7525	0.1592	0.2004	5.0720	0.0395
Nilaparvata lugens (biotype L, Zhejiang, China)	0.7351	2.9447	0.2496	0.5997	3.7079	0.1617	0.2016	5.0870	0.0396
Nilaparvata lugens (Hainan, China)	0.7351	2.9447	0.2496	0.5989	3.7086	0.1615	0.1990	5.0934	0.0391
Nilaparvata lugens (Hadong-gun, Korea)	0.7351	2.9447	0.2496	0.5957	3.7284	0.1598	0.2010	5.0726	0.0396
Nilaparvata lugens (Guangdong, China)	0.7351	2.9447	0.2496	0.5989	3.7086	0.1615	0.2010	5.0726	0.0396
Nilaparvata lugens (biotype 1, Zhejiang, China)	0.7351	2.9447	0.2496	0.5997	3.7079	0.1617	0.2004	5.0720	0.0395
Nilaparvata lugens (biotype 2, Zhejiang, China)	0.7351	2.9447	0.2496	0.5980	3.7281	0.1604	0.2017	5.0794	0.0397
Nilaparvata lugens (biotype 3, Zhejiang, China)	0.7351	2.9447	0.2496	0.5993	3.7069	0.1617	0.2004	5.0720	0.0395
Nilaparvata muiri	0.7793	2.8162	0.2767	0.6161	3.3142	0.1859	0.1957	4.9230	0.0398
Nilaparvata sp.	0.7441	2.8348	0.2625	0.5856	3.5859	0.1633	0.2152	4.9445	0.0435
Peregrinus maidis	0.8022	2.7892	0.2876	0.5808	3.5448	0.1639	0.2030	5.0741	0.0400
Sogatella furcifera (Hainan, China)	0.7494	2.6893	0.2787	0.5592	3.7511	0.1491	0.2010	4.6867	0.0429
Sogatella furcifera (Yunnan, China)	0.7494	2.6893	0.2787	0.5592	3.7511	0.1491	0.2010	4.6886	0.0429
Sogatella vibix	0.7393	2.4768	0.2985	0.5620	4.1048	0.1369	0.2018	4.7887	0.0421
Laodelphax striatellus (Jiangsu, China)	0.8238	2.7494	0.2996	0.5993	3.5119	0.1707	0.1879	5.0591	0.0371
Laodelphax striatella (Suwon, Korea)	0.8238	2.7494	0.2996	0.6014	3.5046	0.1716	0.1895	5.0512	0.0375
Laodelphax striatella (Milyang, Korea)	0.8238	2.7494	0.2996	0.6014	3.5046	0.1716	0.1895	5.0512	0.0375
Laodelphax striatellus A1 (China)	0.8345	2.7817	0.3000	0.6003	3.5082	0.1711	0.1911	4.9829	0.0384
Laodelphax striatellus B1 (China)	0.8238	2.7494	0.2996	0.6033	3.4904	0.1728	0.1910	5.0091	0.0381
Laodelphax striatellus (Beijing, China)	0.7710	2.8706	0.2686	0.6033	3.4806	0.1733	0.1911	4.9829	0.0384

Species	*COII*			*COIII*			*CytB*		
	Ka	Ks	Ka/Ks	Ka	Ks	Ka/Ks	Ka	Ks	Ka/Ks
**Fulgoroidea**									
**Flatidae**									
Flatinae									
Metcalfa pruinosa (H1)	0.5281	4.3420	0.1216	0.2950	5.2737	0.0559	0.3262	3.5787	0.0911
Metcalfa pruinosa (H3)	0.5281	4.3420	0.1216	0.2950	5.2737	0.0559	0.3264	3.5844	0.0911
Salurnis marginella	0.4753	4.5306	0.1049	0.3221	5.0634	0.0636	0.3124	3.5300	0.0885
Geisha distinctissima	0.4958	4.1545	0.1193	0.3327	5.3099	0.0627	0.3513	3.3519	0.1048
**Fulgoridae**									
Fulgorinae									
Pyrops candelaria	0.5185	4.1031	0.1264	0.3429	4.9884	0.0687	0.3647	3.2293	0.1129
Aphaeninae									
Lycorma delicatula (Henan, China)	0.4892	4.2325	0.1156	0.3578	5.3375	0.0670	0.3471	3.4206	0.1015
Lycorma delicatula (China)	0.4898	4.1960	0.1167	0.3545	5.2899	0.0670	0.3453	3.4466	0.1002
Lycorma delicatula (Korea)	0.4892	4.2325	0.1156	0.3545	5.2899	0.0670	0.3364	3.4824	0.0966
Aphaena (*Callidepsa*) *amabilis*	0.5020	4.4094	0.1138	0.3650	5.1453	0.0709	0.3336	3.6326	0.0918
Aphaena (*Aphaena*) *discolor nigrotibiata*	0.5160	4.0752	0.1266	0.3752	5.2040	0.0721	0.3238	3.6367	0.0890
**Achilidae**									
Achilinae									
Betatropis formosana	0.4961	4.2799	0.1159	0.3501	5.6812	0.0616	0.3137	3.7225	0.0843
Magadhaideus luodiana sp. nov	0.4882	4.1130	0.1187	0.3696	4.6259	0.0799	0.3203	3.3723	0.0950
Peltatavertexalis horizontalis sp. nov	0.4614	4.6407	0.0994	0.3539	5.3724	0.0659	0.3328	3.4847	0.0955
Plectoderini sp.	0.5097	3.7807	0.1348	0.3544	4.5905	0.0772	0.3329	3.4665	0.0960
Paracatonidia sp.	0.4626	4.6754	0.0989	0.3408	5.2768	0.0646	0.3349	3.5229	0.0951
**Cixiidae**									
Cixiinae									
Pentastiridius sp.	0.4759	4.9587	0.0960	0.3684	5.0953	0.0723	0.3029	3.6567	0.0828
**Derbidae**									
Otiocerinae									
Lydda sp.	0.5422	4.1631	0.1302	0.3841	4.9201	0.0781	0.3219	3.7927	0.0849
**Issidae**									
Issinae									
Sivaloka damnosus	0.4597	4.2923	0.1071	0.3464	5.3822	0.0644	0.3186	3.6038	0.0884
Sivaloka sp.	0.4606	4.2368	0.1087	0.3390	4.9745	0.0681	0.2973	3.6556	0.0813
**Ricaniidae**									
Ricaniinae									
Ricania marginalis	0.4710	4.9596	0.0950	0.3277	5.1898	0.0631	0.3484	3.5951	0.0969
Ricania speculum	0.4618	5.0527	0.0914	0.3301	5.1930	0.0636	0.3174	3.5137	0.0903
**Delphacidae**									
Asiracinae									
Ugyops sp.	0.5223	4.4860	0.1164	0.3597	5.1603	0.0697	0.3213	3.7920	0.0847
Delphacinae									
Saccharosydnini									
Saccharosydne procerus	0.4763	4.8727	0.0977	0.3824	5.1200	0.0747	0.3143	3.9716	0.0791
Delphacini									
Changeondelphax velitchkovskyi	0.5254	3.8401	0.1368	0.3862	4.9547	0.0780	0.3167	3.4521	0.0917
Nilaparvata bakeri (unknown)	0.4747	4.5709	0.1039	0.3727	5.0557	0.0737	0.2832	3.7544	0.0754
Nilaparvata lugens (biotype Y, Zhejiang, China)	0.4842	4.3702	0.1108	0.3835	4.5503	0.0843	0.2740	3.7208	0.0737
Nilaparvata lugens (biotype L, Zhejiang, China)	0.4843	4.3972	0.1101	0.3839	4.6101	0.0833	0.2748	3.7236	0.0738
Nilaparvata lugens (Hainan, China)	0.4843	4.3972	0.1101	0.3853	4.5231	0.0852	0.2747	3.7310	0.0736
Nilaparvata lugens (Hadong-gun, Korea)	0.4842	4.4271	0.1094	0.3846	4.5634	0.0843	0.2743	3.7475	0.0732
Nilaparvata lugens (Guangdong, China)	0.4843	4.3972	0.1101	0.3853	4.5231	0.0852	0.2763	3.7496	0.0737
Nilaparvata lugens (biotype 1, Zhejiang, China)	0.4842	4.3702	0.1108	0.3835	4.5503	0.0843	0.2747	3.7310	0.0736
Nilaparvata lugens (biotype 2, Zhejiang, China)	0.4843	4.3972	0.1101	0.3835	4.5503	0.0843	0.2747	3.7310	0.0736
Nilaparvata lugens (biotype 3, Zhejiang, China)	0.4843	4.3972	0.1101	0.3877	4.5124	0.0859	0.2747	3.7310	0.0736
Nilaparvata muiri	0.4704	4.3696	0.1076	0.3798	4.7788	0.0795	0.2763	3.6498	0.0757
Nilaparvata sp.	0.4926	4.5392	0.1085	0.3968	4.5164	0.0879	0.2882	3.7603	0.0767
Peregrinus maidis	0.4946	4.4142	0.1120	0.4176	4.6882	0.0891	0.3135	3.7649	0.0833
Sogatella furcifera (Hainan, China)	0.5038	4.3015	0.1171	0.3824	4.7262	0.0809	0.2978	3.7939	0.0785
Sogatella furcifera (Yunnan, China)	0.5038	4.3015	0.1171	0.3863	4.7097	0.0820	0.2980	3.7851	0.0787
Sogatella vibix	0.4966	4.7401	0.1048	0.3909	4.8843	0.0800	0.2900	4.0531	0.0715
Laodelphax striatellus (Jiangsu, China)	0.4804	4.3784	0.1097	0.3884	5.1321	0.0757	0.2713	3.9399	0.0688
Laodelphax striatella (Suwon, Korea)	0.4792	4.4180	0.1085	0.3883	5.1559	0.0753	0.2698	3.9587	0.0681
Laodelphax striatella (Milyang, Korea)	0.4792	4.4180	0.1085	0.3883	5.1559	0.0753	0.2698	3.9587	0.0681
Laodelphax striatellus A1 (China)	0.4891	4.3286	0.1130	0.3883	5.1225	0.0758	0.2690	3.9498	0.0681
Laodelphax striatellus B1 (China)	0.4879	4.3311	0.1127	0.3886	5.1646	0.0752	0.2711	3.9401	0.0688
Laodelphax striatellus (Beijing, China)	0.4806	4.4657	0.1076	0.3883	5.1225	0.0758	0.2676	3.9605	0.0676

Species	ND1			ND2			ND3		
	Ka	Ks	Ka/Ks	Ka	Ks	Ka/Ks	Ka	Ks	Ka/Ks
**Fulgoroidea**									
**Flatidae**									
Flatinae									
Metcalfa pruinosa (H1)	0.7433	2.9953	0.2482	0.7381	2.7699	0.2665	0.5795	4.0748	0.1422
Metcalfa pruinosa (H3)	0.7433	2.9953	0.2482	0.7364	2.7867	0.2643	0.5795	4.0748	0.1422
Salurnis marginella	0.7625	2.9579	0.2578	0.7856	2.6485	0.2966	0.7215	3.0602	0.2358
Geisha distinctissima	0.7680	2.5742	0.2984	0.7523	2.5583	0.2941	0.5847	4.4094	0.1326
**Fulgoridae**									
Fulgorinae									
Pyrops candelaria	0.7569	2.7323	0.2770	0.7531	2.5694	0.2931	0.7535	2.9102	0.2589
Aphaeninae									
Lycorma delicatula (Henan, China)	0.7342	3.2810	0.2238	0.7852	2.5417	0.3089	0.7182	2.7934	0.2571
Lycorma delicatula (China)	0.7275	3.2746	0.2222	0.7836	2.5761	0.3042	0.6621	3.4249	0.1933
Lycorma delicatula (Korea)	0.7275	3.2746	0.2222	0.7836	2.5761	0.3042	0.6621	3.4249	0.1933
Aphaena (*Callidepsa*) *amabilis*	0.7287	3.6783	0.1981	0.7626	2.9359	0.2598	0.7012	3.3337	0.2103
Aphaena (*Aphaena*) *discolor nigrotibiata*	0.7192	3.3786	0.2129	0.7953	2.6605	0.2989	0.6022	4.2790	0.1407
**Achilidae**									
Achilinae									
Betatropis formosana	0.7389	3.1962	0.2312	0.7505	2.8243	0.2657	0.6409	3.8930	0.1646
Magadhaideus luodiana sp. nov	0.7511	2.6784	0.2804	0.7204	2.7537	0.2616	0.5454	3.9422	0.1384
Peltatavertexalis horizontalis sp. nov	0.7471	2.6753	0.2793	0.7879	2.3755	0.3317	0.6361	3.8830	0.1638
Plectoderini sp.	0.7663	2.8645	0.2675	0.7201	3.0515	0.2360	0.6348	3.5247	0.1801
Paracatonidia sp.	0.7289	3.2385	0.2251	0.7325	3.0017	0.2440	0.5836	3.7848	0.1542
**Cixiidae**									
Cixiinae									
Pentastiridius sp.	-	-	-	0.8215	2.3527	0.3492	0.7140	3.1575	0.2261
**Derbidae**									
Otiocerinae									
Lydda sp.	0.7295	3.1217	0.2337	0.6995	3.1414	0.2227	0.5963	4.2188	0.1413
**Issidae**									
Issinae									
Sivaloka damnosus	0.7698	2.5477	0.3022	0.7788	2.4506	0.3178	0.5957	3.6781	0.1619
Sivaloka sp.	0.7464	2.8770	0.2594	0.7283	2.9694	0.2453	0.6102	3.9104	0.1560
**Ricaniidae**									
Ricaniinae									
Ricania marginalis	0.7965	2.3493	0.3391	0.7897	2.5311	0.3120	0.6009	3.5970	0.1671
Ricania speculum	0.7497	3.0184	0.2484	0.7174	2.7608	0.2598	0.5905	3.6965	0.1598
**Delphacidae**									
Asiracinae									
Ugyops sp.	0.7173	3.7132	0.1932	0.7716	3.1038	0.2486	0.7029	3.4375	0.2045
Delphacinae									
Saccharosydnini									
Saccharosydne procerus	0.7869	3.1631	0.2488	0.7697	3.1352	0.2455	0.6566	3.9879	0.1647
Delphacini									
Changeondelphax velitchkovskyi	0.8250	2.2407	0.3682	0.7390	2.7437	0.2693	0.6240	3.7289	0.1673
Nilaparvata bakeri (unknown)	0.7865	2.7383	0.2872	0.6849	3.0876	0.2218	0.8576	1.6783	0.5110
Nilaparvata lugens (biotype Y, Zhejiang, China)	0.7792	2.6582	0.2931	0.6978	2.9752	0.2345	0.8345	1.7986	0.4640
Nilaparvata lugens (biotype L, Zhejiang, China)	0.7792	2.6582	0.2931	0.6972	2.9932	0.2329	0.8351	1.8192	0.4590
Nilaparvata lugens (Hainan, China)	0.7696	2.7356	0.2813	0.7060	2.8638	0.2465	0.6171	4.2903	0.1438
Nilaparvata lugens (Hadong-gun, Korea)	0.7890	2.6178	0.3014	0.6903	3.0320	0.2277	0.6171	4.2903	0.1438
Nilaparvata lugens (Guangdong, China)	0.7781	2.6793	0.2904	0.6903	3.0320	0.2277	0.6171	4.2903	0.1438
Nilaparvata lugens (biotype 1, Zhejiang, China)	0.7792	2.6582	0.2931	0.6978	2.9752	0.2345	0.8345	1.7986	0.4640
Nilaparvata lugens (biotype 2, Zhejiang, China)	0.7792	2.6582	0.2931	0.7002	2.9676	0.2359	0.8345	1.7986	0.4640
Nilaparvata lugens (biotype 3, Zhejiang, China)	0.7789	2.6420	0.2948	0.6986	2.9795	0.2345	0.8345	1.7986	0.4640
Nilaparvata muiri	0.7698	2.9717	0.2590	0.7095	2.8354	0.2502	0.8427	1.7448	0.4830
Nilaparvata sp.	0.7646	2.8362	0.2696	0.7501	2.9173	0.2571	0.6951	3.1758	0.2189
Peregrinus maidis	0.7690	2.7189	0.2829	0.6995	3.1414	0.2227	0.6830	3.1383	0.2176
Sogatella furcifera (Hainan, China)	0.7503	2.8622	0.2621	0.7309	2.5883	0.2824	0.6465	3.6730	0.1760
Sogatella furcifera (Yunnan, China)	0.7503	2.8622	0.2621	0.7323	2.5749	0.2844	0.6465	3.6730	0.1760
Sogatella vibix	0.7758	2.8903	0.2684	0.7323	2.7089	0.2703	0.6455	3.4690	0.1861
Laodelphax striatellus (Jiangsu, China)	0.7841	2.4796	0.3162	0.7917	2.4243	0.3266	0.7743	2.8012	0.2764
Laodelphax striatella (Suwon, Korea)	0.7797	2.5489	0.3059	0.7934	2.4198	0.3279	0.7743	2.8012	0.2764
Laodelphax striatella (Milyang, Korea)	0.7797	2.5489	0.3059	0.7934	2.4198	0.3279	0.7743	2.8012	0.2764
Laodelphax striatellus A1 (China)	0.7843	2.4818	0.3160	0.7919	2.4439	0.3240	0.7812	2.7258	0.2866
Laodelphax striatellus B1 (China)	0.7852	2.4948	0.3147	0.7934	2.4198	0.3279	0.7743	2.8012	0.2764
Laodelphax striatellus (Beijing, China)	0.7843	2.4818	0.3160	0.7889	2.4557	0.3213	0.7812	2.7258	0.2866

Species	ND4			ND4L			ND5		
	Ka	Ks	Ka/Ks	Ka	Ks	Ka/Ks	Ka	Ks	Ka/Ks
**Fulgoroidea**									
**Flatidae**									
Flatinae									
Metcalfa pruinosa (H1)	0.5568	5.7317	0.0971	0.7511	2.4527	0.3063	0.5655	5.8165	0.0972
Metcalfa pruinosa (H3)	0.5568	5.7317	0.0971	0.7511	2.4527	0.3063	0.5665	5.8752	0.0964
Salurnis marginella	0.5761	5.0915	0.1132	0.6801	3.3647	0.2021	0.5583	5.9332	0.0941
Geisha distinctissima	0.5936	4.6408	0.1279	0.8935	2.8299	0.3157	0.6086	5.4935	0.1108
**Fulgoridae**									
Fulgorinae									
Pyrops candelaria	0.6262	4.2032	0.1490	0.9379	1.3262	0.7072	0.5491	5.3324	0.1030
Aphaeninae									
Lycorma delicatula (Henan, China)	0.6187	5.3665	0.1153	0.7338	3.2573	0.2253	0.5789	5.8056	0.0997
Lycorma delicatula (China)	0.6141	5.3557	0.1147	0.7338	3.2573	0.2253	0.5045	6.5611	0.0769
Lycorma delicatula (Korea)	0.6121	5.4154	0.1130	0.7338	3.2573	0.2253	0.5030	6.6071	0.0761
Aphaena (*Callidepsa*) *amabilis*	0.6021	5.8064	0.1037	0.7996	2.3507	0.3402	0.5249	6.3016	0.0833
Aphaena (*Aphaena*) *discolor nigrotibiata*	0.5995	5.2975	0.1132	0.8243	2.0809	0.3961	0.5382	6.3431	0.0848
**Achilidae**									
Achilinae									
Betatropis formosana	0.5549	5.5192	0.1005	0.8626	1.8636	0.4629	0.5603	5.6525	0.0991
Magadhaideus luodiana sp. nov	0.5833	4.3929	0.1328	0.7824	2.1692	0.3607	0.5487	5.1342	0.1069
Peltatavertexalis horizontalis sp. nov	0.6161	4.3607	0.1413	0.8200	2.2149	0.3702	0.5321	5.9514	0.0894
Plectoderini sp.	0.5995	4.8404	0.1239	0.7772	2.4131	0.3221	0.5460	5.5023	0.0992
Paracatonidia sp.	0.6098	4.9776	0.1225	0.7725	2.1490	0.3595	0.5560	6.1192	0.0909
**Cixiidae**									
Cixiinae									
Pentastiridius sp.	0.5935	4.8595	0.1221	0.7113	4.9334	0.1442	0.5215	5.2072	0.1002
**Derbidae**									
Otiocerinae									
Lydda sp.	0.5896	4.9327	0.1195	0.7422	2.5342	0.2929	0.5240	6.1825	0.0848
**Issidae**									
Issinae									
Sivaloka damnosus	0.5862	4.5736	0.1282	0.8198	2.2727	0.3607	0.5829	5.4469	0.1070
Sivaloka sp.	0.5545	4.9512	0.1120	0.8198	2.2727	0.3607	0.5331	6.2588	0.0852
**Ricaniidae**									
Ricaniinae									
Ricania marginalis	0.6460	3.7507	0.1722	0.7221	3.4670	0.2083	0.5512	5.5338	0.0996
Ricania speculum	0.5875	4.6132	0.1274	0.7221	3.4670	0.2083	0.5239	5.9654	0.0878
**Delphacidae**									
Asiracinae									
Ugyops sp.	0.5868	6.0838	0.0964	0.7730	3.2046	0.2412	0.5429	6.4828	0.0837
Delphacinae									
Saccharosydnini									
Saccharosydne procerus	0.5760	5.1814	0.1112	0.7903	3.7234	0.2122	0.6082	4.8469	0.1255
Delphacini									
Changeondelphax velitchkovskyi	0.6087	4.7942	0.1270	0.8070	2.6923	0.2997	0.6034	4.6229	0.1305
Nilaparvata bakeri (unknown)	0.5925	4.9115	0.1206	0.8492	2.1816	0.3893	0.5833	5.6170	0.1038
Nilaparvata lugens (biotype Y, Zhejiang, China)	0.5915	4.4901	0.1317	0.8295	2.3812	0.3483	0.5862	5.5455	0.1057
Nilaparvata lugens (biotype L, Zhejiang, China)	0.5927	4.4838	0.1322	0.8295	2.3812	0.3483	0.5870	5.5414	0.1059
Nilaparvata lugens (Hainan, China)	0.5877	4.5146	0.1302	0.8312	2.3877	0.3481	0.5875	5.5253	0.1063
Nilaparvata lugens (Hadong-gun, Korea)	0.5874	4.5575	0.1289	0.8295	2.3812	0.3483	0.5867	5.5770	0.1052
Nilaparvata lugens (Guangdong, China)	0.5877	4.5146	0.1302	0.8183	2.4768	0.3304	0.5881	5.5481	0.1060
Nilaparvata lugens (biotype 1, Zhejiang, China)	0.5912	4.5102	0.1311	0.8295	2.3812	0.3483	0.5862	5.5455	0.1057
Nilaparvata lugens (biotype 2, Zhejiang, China)	0.5915	4.4901	0.1317	0.8295	2.3812	0.3483	0.5879	5.5266	0.1064
Nilaparvata lugens (biotype 3, Zhejiang, China)	0.5891	4.5385	0.1298	0.8295	2.3812	0.3483	0.5875	5.5253	0.1063
Nilaparvata muiri	0.5864	4.4297	0.1324	0.9134	1.6130	0.5663	0.5734	5.5872	0.1026
Nilaparvata sp.	0.5939	5.0614	0.1173	0.8029	2.0852	0.3850	0.6215	4.6709	0.1331
Peregrinus maidis	0.6093	4.5926	0.1327	0.7978	2.9282	0.2725	0.6018	4.8365	0.1244
Sogatella furcifera (Hainan, China)	0.5736	5.1879	0.1106	0.8167	2.2186	0.3681	0.6144	4.2756	0.1437
Sogatella furcifera (Yunnan, China)	0.5699	5.2747	0.1080	0.8167	2.2186	0.3681	0.6145	4.2924	0.1432
Sogatella vibix	0.6047	4.8459	0.1248	0.7745	2.2048	0.3513	0.5838	5.0983	0.1145
Laodelphax striatellus (Jiangsu, China)	0.5958	5.1282	0.1162	0.9814	1.1750	0.8352	0.6286	5.0679	0.1240
Laodelphax striatella (Suwon, Korea)	0.6007	5.0348	0.1193	0.9814	1.1750	0.8352	0.6247	5.0972	0.1225
Laodelphax striatella (Milyang, Korea)	0.6007	5.0348	0.1193	0.9814	1.1750	0.8352	0.6247	5.0972	0.1225
Laodelphax striatellus A1 (China)	0.5959	5.1194	0.1164	0.9483	1.5185	0.6245	0.6269	5.0740	0.1235
Laodelphax striatellus B1 (China)	0.6007	5.0348	0.1193	0.9814	1.1750	0.8352	0.6247	5.0972	0.1225
Laodelphax striatellus (Beijing, China)	0.6255	4.7742	0.1310	0.9602	1.3670	0.7025	0.6353	4.9438	0.1285

Species	ND6		
	Ka	Ks	Ka/Ks
**Fulgoroidea**			
**Flatidae**			
Flatinae			
Metcalfa pruinosa (H1)	0.8362	2.1858	0.3826
Metcalfa pruinosa (H3)	0.8362	2.1858	0.3826
Salurnis marginella	0.8073	2.4032	0.3359
Geisha distinctissima	0.7782	2.6300	0.2959
**Fulgoridae**			
Fulgorinae			
Pyrops candelaria	0.7912	2.7288	0.2900
Aphaeninae			
Lycorma delicatula (Henan, China)	0.8054	2.4521	0.3285
Lycorma delicatula (China)	0.8054	2.4521	0.3285
Lycorma delicatula (Korea)	0.8054	2.4521	0.3285
Aphaena (*Callidepsa*) *amabilis*	0.7919	2.8620	0.2767
Aphaena (*Aphaena*) *discolor nigrotibiata*	0.8777	1.9291	0.4550
**Achilidae**			
Achilinae			
Betatropis formosana	0.8356	2.1523	0.3882
Magadhaideus luodiana sp. nov	0.7282	2.6059	0.2795
Peltatavertexalis horizontalis sp. nov	0.7640	2.5709	0.2972
Plectoderini sp.	0.7867	2.4784	0.3174
Paracatonidia sp.	0.8194	2.2793	0.3595
**Cixiidae**			
Cixiinae			
Pentastiridius sp.	0.8918	1.7634	0.5057
**Derbidae**			
Otiocerinae			
Lydda sp.	0.7701	3.1016	0.2483
**Issidae**			
Issinae			
Sivaloka damnosus	0.7884	2.5912	0.3042
Sivaloka sp.	0.7679	2.7103	0.2833
**Ricaniidae**			
Ricaniinae			
Ricania marginalis	0.7380	2.8633	0.2577
Ricania speculum	0.7554	2.7924	0.2705
**Delphacidae**			
Asiracinae			
Ugyops sp.	0.8045	2.5844	0.3113
Delphacinae			
Saccharosydnini			
Saccharosydne procerus	0.8754	2.1813	0.4013
Delphacini			
Changeondelphax velitchkovskyi	0.7774	2.3893	0.3254
Nilaparvata bakeri (unknown)	0.8356	2.1929	0.3810
Nilaparvata lugens (biotype Y, Zhejiang, China)	0.8412	2.0618	0.4080
Nilaparvata lugens (biotype L, Zhejiang, China)	0.8336	2.1123	0.3947
Nilaparvata lugens (Hainan, China)	0.8313	2.1426	0.3880
Nilaparvata lugens (Hadong-gun, Korea)	0.8313	2.1426	0.3880
Nilaparvata lugens (Guangdong, China)	0.8313	2.1426	0.3880
Nilaparvata lugens (biotype 1, Zhejiang, China)	0.8313	2.1426	0.3880
Nilaparvata lugens (biotype 2, Zhejiang, China)	0.8313	2.1426	0.3880
Nilaparvata lugens (biotype 3, Zhejiang, China)	0.8313	2.1426	0.3880
Nilaparvata muiri	0.8261	2.1859	0.3779
Nilaparvata sp.	0.8335	2.2157	0.3762
Peregrinus maidis	0.8188	2.4191	0.3385
Sogatella furcifera (Hainan, China)	0.7844	2.5354	0.3094
Sogatella furcifera (Yunnan, China)	0.7844	2.5354	0.3094
Sogatella vibix	0.7513	2.6016	0.2888
Laodelphax striatellus (Jiangsu, China)	0.8232	2.3496	0.3504
Laodelphax striatella (Suwon, Korea)	0.8199	2.4058	0.3408
Laodelphax striatella (Milyang, Korea)	0.8199	2.4058	0.3408
Laodelphax striatellus A1 (China)	0.8199	2.4058	0.3408
Laodelphax striatellus B1 (China)	0.8199	2.4058	0.3408
Laodelphax striatellus (Beijing, China)	0.8521	2.1230	0.4013

*Stop codon was excluded in the count.
