# Supplementary material for: Complete Mitochondrial Genomes of Metcalfa pruinosa and Salurnis marginella (Hemiptera: Flatidae): Genomic Comparison and Phylogenetic Inference in Fulgoroidea
Source: Curr Issues Mol Biol. 2021 Sep 30;43(3):1391–418. doi: 10.3390/cimb43030099 (PMC8929015; doi:10.3390/cimb43030099)
Supplement: Supplementary file 1 [file cimb-43-00099-s001.zip › Sup. Table 8. KaKs ANOVA-MJ.pdf]

Table S8

ANOVA test for mean Ks/Ks ratio of 45 mitochondrial genomes of Fulgoroidea.

(A) Whole PCGs, major-strand PCGs, and minor-strand PCGs

Factor	No.	Mean	Std Dev.	Lower	Upper	Lower 95%	Upper 95%	Prob > F	Min.	Med.	Max.	Connecting Latter
PCGs	45	0.0977	0.0062	0.0915	0.1040	0.0958	0.0996		0.0826	0.0988	0.1122	A
PCGs-major	45	0.0841	0.0063	0.0778	0.0904	0.0823	0.0860	<.0001	0.0713	0.0859	0.0953	B
PCGs-minor	45	0.1131	0.0114	0.1017	0.1245	0.1097	0.1165		0.0875	0.1156	0.1566	C

(B) Individual PCGs

Factor	No.	Mean	Std Dev.	Lower	Upper	Lower 95%	Upper 95%	Prob > F	Min.	Med.	Max.	Connecting Latter
ATP6	44	0.1469	0.0213	0.1257	0.1682	0.1405	0.1534		0.1000	0.1522	0.1859	C
ATP8	44	0.3340	0.1115	0.2224	0.4455	0.3000	0.3679		0.2352	0.2955	0.6839	A
COI	44	0.0418	0.0035	0.0384	0.0453	0.0408	0.0429		0.0371	0.0419	0.0534	E
COII	44	0.1120	0.0098	0.1022	0.1218	0.1090	0.1150		0.0914	0.1101	0.1368	CD
COIII	44	0.0741	0.0088	0.0653	0.0829	0.0714	0.0768		0.0559	0.0753	0.0891	DE
CytB	44	0.0837	0.0114	0.0723	0.0951	0.0802	0.0872		0.0676	0.0831	0.1129	DE
ND1	43	0.2716	0.0373	0.2343	0.3088	0.2601	0.2830	<.0001	0.1932	0.2770	0.3682	B
ND2	44	0.2725	0.0367	0.2358	0.3092	0.2614	0.2837		0.2218	0.2650	0.3492	B
ND3	44	0.2358	0.1136	0.1222	0.3493	0.2012	0.2703		0.1326	0.1897	0.5110	B
ND4	44	0.1222	0.0140	0.1081	0.1362	0.1179	0.1264		0.0964	0.1223	0.1722	CD
ND4L	44	0.3808	0.1710	0.2098	0.5518	0.3288	0.4328		0.1442	0.3483	0.8352	A
ND5	44	0.1056	0.0168	0.0888	0.1224	0.1005	0.1107		0.0761	0.1055	0.1437	CD
ND6	44	0.3464	0.0536	0.2927	0.4000	0.3301	0.3627		0.2483	0.3408	0.5057	A

PCG, protein-coding gene.
