# Supplementary material for: Complete Mitochondrial Genomes of Metcalfa pruinosa and Salurnis marginella (Hemiptera: Flatidae): Genomic Comparison and Phylogenetic Inference in Fulgoroidea
Source: Curr Issues Mol Biol. 2021 Sep 30;43(3):1391–418. doi: 10.3390/cimb43030099 (PMC8929015; doi:10.3390/cimb43030099)
Supplement: Supplementary file 1 [file cimb-43-00099-s001.zip › Sup. Table 9. Overlap-Space of Fulgoroidea.pdf]

Table S9
Overlapping and intergenic-spacer sequences of Fulgoroidea mitochondrial genomes (1/11).

Region	*Metcalfa pruinosa* (H1)	*Metcalfa pruinosa* (H3)	*Salurnis marginella*	*Geisha distinctissima*‡	*Pyrops candelaria*
trnI - *trnQ*	(I)3	(I)3	(I)18	(O)1	(I)9
trnQ - *trnM*	(O)1	(O)1	(O)1	(O)1	(I)4
trnM - *ND2*					
ND2- *trnW*	(O)2	(O)2	(O)2	(O)2	(I)33
trnW - *trnC*	(O)8	(O)8	(O)8	(O)8	(O)8
trnC - *trnY*	(I)7	(I)7		(I)3	
trnY - *COI*			(I)5	(I)6	(I)2
COI - *trnL*$_2$	(I)2	(I)2			
trnL$_2$ - *COII*				(I)1	(I)8
COII - *trnK*	(I)10	(I)10		(I)2	(I)2
trnK - *trnD*					
trnD - *ATP8*					
ATP8- *ATP6*	(O)7	(O)7	(O)7	(O)7	(O)7
ATP6- *COIII*					
COIII - *trnG*	(I)6	(I)6	(I)1		(I)6
trnG - *ND3*	(I)1	(I)1	(I)35		
ND3 - *trnA*	(O)2	(O)2	(O)1		(I)17
trnA - *trnR*	(I)17	(I)17			(I)4
trnR - *trnN*	(I)25	(I)25	(I)1	(I)5	
trnN - *trnS*$_1$	(I)1	(I)1	(O)2		(O)2
trnS$_1$- *trnE*	(I)3	(I)3	(I)2	(I)2	(I)2
trnE - *trnF*	(I)12	(I)12	(O)2	(I)3	(O)2
trnF - *ND5*	(I)6	(I)6	(O)1	(O)10	
ND5 - *trnH*	(I)1	(I)1		(I)9	
trnH - *ND4*	(I)14	(I)14			(I)4
ND4 - *ND4L*	(O)1	(O)1	(I)1	(I)8	(O)7
ND4L - *trnT*	(I)2	(I)2	(I)2	(I)2	(I)16
trnT - *trnP*	(I)7	(I)7	(I)1	(I)3	(I)3
trnP - *ND6*	(I)1	(I)1	(I)1	(O)10	(I)1
ND6 - *CytB*	(I)4	(I)4	(O)8	(O)8	(I)3
CytB - *trnS*$_2$	(I)15	(I)15	(O)1		(I)17
trnS$_2$ - *ND1*	(I)9	(I)9	(O)7	(I)6	(O)7
ND1 - *trnL*$_1$	(I)1	(I)1	(I)1	(I)1	(I)1
trnL$_1$ - *lrRNA*					
lrRNA - *trnV*					
trnV - *srRNA*			(O)1		
srRNA - A+T-rich region					
Total nucleotides	(O)21<br>(I)147	(O)21<br>(I)147	(O)41<br>(I)68	(O)47<br>(I)51	(O)33<br>(I)132

Table S9

Continued (2/11).

Region	*Lycorma delicatula* (Henan, China)	*Lycorma delicatula* (China)	*Lycorma delicatula* (Korea)	*Aphaena* (Callidepsa) *amabilis*	*Aphaena* (Aphaena) *discolor nigrotibiata*
trnI - trnQ	(I)2	(I)2	(I)2	(I)2	(I)2
trnQ - trnM	(O)1	(O)1	(O)1	(O)1	(O)1
trnM - ND2					
ND2- trnW	(I)2	(I)2	(I)2	(O)2	(I)2
trnW - trnC	(O)8	(O)8	(O)8	(O)8	(O)8
trnC - trnY				(I)9	(I)3
trnY - COI	(I)4	(I)4	(I)4	(I)4	(I)5
COI - trnL$_2$					
trnL$_2$ - COII					
COII - trnK	(I)10	(I)10	(I)10	(I)9	(I)9
trnK - trnD					
trnD - ATP8					
ATP8- ATP6	(O)7	(O)7	(O)7	(O)7	(O)7
ATP6- COIII					
COIII - trnG	(I)5	(I)2	(I)2	(I)3	(I)7
trnG - ND3					
ND3 - trnA	(I)25		(O)1	(I)6	(O)1
trnA - trnR	(O)4			(I)1	(I)1
trnR - trnN	(O)1	(O)1	(O)1	(I)7	(I)8
trnN - trnS$_1$	(O)2	(O)1	(O)2	(O)1	(O)1
trnS$_1$- trnE	(O)2	(O)1	(O)2	(O)1	(O)1
trnE - trnF	(O)2	(O)2	(O)2	(O)2	(O)2
trnF - ND5	(O)10			(I)1	(I)2
ND5 - trnH	(O)1	(O)1	(O)1		
trnH - ND4	(O)16			(I)1	(I)1
ND4 - ND4L	(O)7	(O)7	(O)7	(O)7	(O)7
ND4L - trnT	(I)7	(I)7	(I)7	(I)7	(I)7
trnT - trnP	(I)3	(I)3	(I)3	(I)6	(I)2
trnP - ND6	(I)1	(I)1	(I)1	(I)1	(I)1
ND6 - CytB	(O)8	(O)8	(I)4	(O)8	(O)8
CytB - trnS$_2$	(I)5	(I)5	(I)5	(I)1	
trnS$_2$ - ND1	(O)8	(O)8	(O)8	(I)3	(I)4
ND1 - trnL$_1$	(I)1	(I)1	(I)1	(I)1	(I)1
trnL$_1$ - lrRNA					
lrRNA - trnV					
trnV - srRNA			(O)3		
srRNA - A+T-rich region					
Total nucleotides	(O)77<br>(I)65	(O)45<br>(I)37	(O)43<br>(I)41	(O)37<br>(I)62	(O)36<br>(I)55

Table S9

Continued (3/11).

Region	*Betatropis formosana*	*Magadhaideus luodiana* sp. nov	*Peltatavertexalis horizontalis* sp. nov	*Plectoderini* sp.	*Paracatonidia* sp.
trnI - *trnQ*	(O)3	(I)2	(O)3	(O)3	(O)3
trnQ - *trnM*	(O)1	(O)1	(O)1	(O)1	(O)1
trnM - *ND2*					
ND2- *trnW*	(O)2	(O)2	(O)2	(O)2	(O)2
trnW - *trnC*	(O)8	(O)8	(O)8	(O)8	(O)8
trnC - *trnY*	(I)12	(I)8	(I)5	(I)8	(I)3
trnY - *COI*	(I)16			(I)2	
COI - $trnL_2$					
$trnL_2$ - *COII*					
COII - *trnK*				(I)1	
trnK - *trnD*					
trnD - *ATP8*					
ATP8- *ATP6*	(O)7	(O)7	(O)7	(O)7	(O)7
ATP6- *COIII*					
COIII - *trnG*	(O)1				
trnG - *ND3*	(I)6				
ND3 - *trnA*	(O)2		(I)3	(O)1	(O)1
trnA - *trnR*	(I)6		(I)3	(I)5	
trnR - *trnN*	(I)2	(I)12	(I)1	(I)6	
trnN - $trnS_1$					(O)1
$trnS_1$- *trnE*	(I)6	(I)8			(I)3
trnE - *trnF*	(I)2	(O)1	(I)4	(O)1	(I)3
trnF - *ND5*	(I)10	(I)2	(O)6	(I)2	(O)20
ND5 - *trnH*	(I)5	(I)8	(I)16	(I)3	(O)7
trnH - *ND4*	(I)1	(I)1	(I)1	(I)1	(I)1
ND4 - *ND4L*	(O)7	(O)7	(I)45	(I)48	(I)69
ND4L - *trnT*	(I)2	(I)2	(I)2	(I)1	(I)3
trnT - *trnP*	(I)7	(I)2			(I)1
trnP - *ND6*	(I)1	(I)1	(I)1	(I)1	(I)1
ND6 - *CytB*	(O)8	(O)8	(O)8	(O)8	(I)6
CytB - $trnS_2$	(O)1	(O)1	(O)1	(O)1	(O)1
$trnS_2$ - *ND1*	(O)1	(O)4	(O)2	(O)7	(I)5
ND1 - $trnL_1$	(I)1	(I)1	(I)1	(I)1	(I)1
$trnL_1$ - *lrRNA*	(I)18	(I)3		(I)3	
lrRNA - *trnV*	(I)13	(I)7	(I)1008	(I)15	(I)18
trnV - *srRNA*	(O)3	(O)3	(O)3	(O)3	(O)3
srRNA - A+T-rich region					
Total nucleotides	(O)44<br>(I)108	(O)42<br>(I)57	(O)41<br>(I)1090	(O)42<br>(I)97	(O)54<br>(I)114

Table S9

Continued (4/11).

Region	*Pentastiridius* sp.‡	*Lydda* sp.‡	*Sivaloka damnosus*	*Sivaloka* sp.‡	*Ricania marginalis*
trnI - *trnQ*	(O)3	(I)1		(O)1	(I)4
trnQ - *trnM*	(O)2		(O)1	(O)1	(I)14
trnM - *ND2*					
ND2- *trnW*	(O)2	(O)2		(O)1	(I)27
trnW - *trnC*	(O)8	(O)8	(O)9	(O)8	(O)8
trnC - *trnY*		(I)2	(I)16		(I)12
trnY - *COI*	(O)2	(I)1	(I)9	(I)1	(I)1
COI - *trnL₂*	(I)4	(I)1	(O)5	(I)1	(I)1
trnL₂ - *COII*					
COII - *trnK*	(I)1				
trnK - *trnD*		(I)1			(I)2
trnD - *ATP8*					
ATP8- *ATP6*	(O)7	(O)6	(O)7	(O)7	(O)4
ATP6- *COIII*					(O)28
COIII - *trnG*	(O)1		(I)20		(I)3
trnG - *ND3*				(O)1	
ND3 - *trnA*	(O)1	(O)1	(I)6	(O)1	(I)5
trnA - *trnR*	(O)1				
trnR - *trnN*	(O)2	(O)1	(I)13		(O)3
trnN - *trnS₁*	(O)1		(O)1	(O)1	(O)1
trnS₁- *trnE*	(O)1	(I)5	(I)8	(O)1	(I)1
trnE - *trnF*		(O)2	(I)4	(O)2	(I)8
trnF - *ND5*	(I)84		(I)6	(O)2	(I)6
ND5 - *trnH*	(I)140		(I)5		(I)1
trnH - *ND4*					
ND4 - *ND4L*	(I)5	(I)5	(O)7	(I)151	(O)7
ND4L - *trnT*	(I)11	(I)2	(I)2	(I)2	(I)2
trnT - *trnP*	(O)1	(O)1			(I)11
trnP - *ND6*	(I)1	(I)1	(I)1	(I)1	(I)1
ND6 - *CytB*	(I)3	(O)8	(O)8	(O)8	(O)8
CytB - *trnS₂*	(I)1	(O)2	(O)1	(O)2	(O)14
trnS₂ - *ND1*			(I)4	(O)7	(O)4
ND1 - *trnL₁*		(I)2	(I)1	(I)1	(I)1
trnL₁ - *lrRNA*		(O)1			
lrRNA - *trnV*					
trnV - *srRNA*		(I)1			
srRNA - A+T-rich region		(O)1			
Total nucleotides	(O)32<br>(I)250	(O)33<br>(I)22	(O)39<br>(I)95	(O)43<br>(I)157	(O)77<br>(I)100

Table S9

Continued (5/11).

Region	*Ricania speculum*	*Ugyops* sp.
trnI - trnQ	(I)4	
trnQ - trnM	(I)14	(O)2
trnM - ND2		
ND2- trnW	(I)26	(I)3
trnW - trnC	(O)8	(O)8
trnC - trnY	(I)4	(I)1
trnY - COI	(I)4	(O)2
COI - trnL$_2$	(I)1	
trnL$_2$ - COII		
COII - trnK		(I)1
trnK - trnD	(I)2	
trnD - ATP8		
ATP8- ATP6	(O)4	(O)4
ATP6- COIII		
COIII - trnG	(I)3	
trnG - ND3		
ND3 - trnA	(I)5	(O)2
trnA - trnR	(I)5	(I)6
trnR - trnN	(I)2	(O)2
trnN - trnS$_1$	(O)1	(O)1
trnS$_1$- trnE	(I)1	(O)1
trnE - trnF	(I)7	(O)2
trnF - ND5	(I)5	
ND5 - trnH	(I)15	
trnH - ND4		
ND4 - ND4L	(O)7	(O)7
ND4L - trnT	(I)2	(I)1
trnT - trnP	(I)11	(O)1
trnP - ND6	(I)1	(I)1
ND6 - CytB	(O)8	
CytB - trnS$_2$	(O)1	(O)1
trnS$_2$ - ND1	(O)7	(I)16
ND1 - trnL$_1$	(I)1	(I)1
trnL$_1$ - lrRNA	(I)23	
lrRNA - trnV	(O)7	
trnV - srRNA	(O)3	
srRNA - A+T-rich region		
Total nucleotides	(O)46<br>(I)136	(O)33<br>(I)30

Table S9

Continued (6/11).

Region	*Saccharosydne procerus*	*Changeondelphax velitchkovskyi*	*Nilaparvata bakeri* (unknown)	*Nilaparvata lugens* (biotype Y, Zhejiang, China)	*Nilaparvata lugens* (biotype L, Zhejiang, China)
trnI - *trnQ*	(I)1	(I)5	(I)3	(I)3	(I)3
trnQ - *trnM*	(O)1	(O)1	(O)1	(O)1	(O)1
trnM - *ND2*					
ND2 - *trnC*	(O)2	(I)3			
trnC - *trnW*	(I)8	(I)28	(I)12	(I)8	(I)7
trnW - *trnY*	(I)14	(I)15	(I)20	(I)20	(I)20
trnY - *COI*	(I)5	(I)8	(I)14	(I)13	(I)13
COI - *trnL$_2$*			(O)5	(O)5	(O)5
trnL$_2$ - *COII*					
COII - *trnK*	(I)2	(I)3	(I)6	(I)11	(I)11
trnK - *trnD*		(I)1			
trnD - *ATP8*					
ATP8- *ATP6*	(O)7	(O)7	(O)22	(O)19	(O)19
ATP6- *COIII*					
COIII - *trnG*					
trnG - *ND3*			(I)101	(I)101	(I)101
ND3 - *trnA*	(I)5	(O)2	(I)8	(I)5	(I)5
trnA - *trnR*		(I)4	(I)4	(I)4	(I)4
trnR - *trnN*	(I)1	(I)3	(O)1	(O)1	(O)1
trnN - *trnS$_1$*	(O)1	(O)1	(O)1	(O)1	(O)1
trnS$_1$- *trnE*	(O)1	(O)1	(O)1	(O)1	(O)1
trnE - *trnF*	(I)4				
trnF - *ND5*					
ND5 - *trnH*			(O)1	(O)1	(O)1
trnH - *ND4*	(I)3				
ND4 - *ND4L*	(O)7	(O)7	(O)10	(O)10	(O)10
ND4L – *ND6*	(I)49	(I)45	(I)34	(I)33	(I)33
ND6 - *trnP*	(I)64	(I)146	(I)70	(I)48	(I)48
trnP - *trnT*	(I)1				
trnT - *CytB*	(I)4	(I)5	(I)8	(I)7	(I)7
CytB - *trnS$_2$*	(I)1	(I)2	(O)10	(O)10	(O)10
trnS$_2$ - *ND1*	(I)15	(I)217	(I)20	(I)20	(I)20
ND1 - *trnL$_1$*	(I)1	(I)1	(I)1	(I)1	(I)1
trnL$_1$ - *lrRNA*					
lrRNA - *trnV*					
trnV - *srRNA*			(I)4	(I)4	(I)4
srRNA - A+T-rich region					
Total nucleotides	(O)19<br>(I)178	(O)19<br>(I)486	(O)52<br>(I)305	(O)49<br>(I)278	(O)49<br>(I)277

Table S9

Continued (7/11).

Region	*Nilaparvata lugens* (Hainan, China)	*Nilaparvata lugens* (Hadong-gun, Korea)[‡]	*Nilaparvata lugens* (Guangdong, China)
trnI - *trnQ*	(I)3	(O)3	(I)3
trnQ - *trnM*	(O)1	(O)1	(O)1
trnM - *ND2*			
ND2 - *trnC*		(O)57	
trnC^{-} *trnC*	(I)348	(I)349	(I)345
trnC^{-} *trnC*	(I)348	(I)346	(I)346
trnC - *trnW*	(I)10	(I)11	(I)10
trnW - *trnY*	(I)9	(I)8	(I)9
trnY - *COI*	(I)13	(I)13	(I)13
COI - *trnL*$_2$			
trnL$_2$ - *COII*			
COII - *trnK*	(I)11	(I)11	(I)11
trnK - *trnD*			
trnD - *ATP8*			
ATP8- *ATP6*	(O)1	(O)1	(O)1
ATP6- *COIII*			
COIII - *trnG*			
trnG - *ND3*			
ND3 - *trnA*	(I)1	(I)1	(I)1
trnA - *trnR*	(I)4	(I)4	(I)4
trnR - *trnN*	(O)1	(O)1	(O)1
trnN - *trnS*$_1$			
trnS$_1$- *trnE*			
trnE - *trnF*			
trnF - *ND5*			
ND5 - *trnH*			
trnH - *ND4*			(O)1
ND4 - *ND4L*	(O)7	(O)7	(O)7
ND4L – *ND6*	(I)33	(I)33	(I)33
ND6 - *trnP*	(I)48	(I)48	(I)48
trnP - *trnT*			
trnT - *CytB*	(I)7	(I)7	(I)7
CytB - *trnS*$_2$	(O)10	(O)10	(O)10
trnS$_2$ - *ND1*	(I)20	(O)15	(I)20
ND1 - *trnL*$_1$	(I)1	(I)1	(I)1
trnL$_1$ - *lrRNA*			
lrRNA - *trnV*	(O)1	(O)1	(O)1
trnV - *srRNA*	(O)1	(O)1	(O)1
srRNA - A+T-rich region			
Total nucleotides	(O)22<br>(I)856	(O)97<br>(I)832	(O)23<br>(I)851

Table S9

Continued (8/11).

Region	*Nilaparvata lugens* (biotype 1, Zhejiang, China)	*Nilaparvata lugens* (biotype 2, Zhejiang, China)	*Nilaparvata lugens* (biotype 3, Zhejiang, China)	*Nilaparvata muiri*	*Nilaparvata* sp.‡
trnI - *trnQ*	(I)3	(I)3	(I)3	(I)2	(I)16
trnQ - *trnM*	(O)1	(O)1	(O)1	(O)1	(O)1
trnM - *ND2*					
ND2 - *trnC*					(O)2
trnC - *trnW*	(I)8	(I)8	(I)8	(I)7	(I)19
trnW - *trnY*	(I)20	(I)20	(I)20	(I)20	(I)3
trnY - *COI*	(I)13	(I)13	(I)13	(I)14	(I)9
COI - *trnL*$_2$	(O)5	(O)5	(O)5	(O)5	
trnL$_2$ - *COII*					
COII - *trnK*	(I)11	(I)11	(I)11	(I)6	(I)1
trnK - *trnD*					
trnD - *ATP8*					(I)1
ATP8- *ATP6*	(O)19	(O)19	(O)19	(O)19	(O)7
ATP6- *COIII*					
COIII - *trnG*					(I)1
trnG - *ND3*	(I)101	(I)101	(I)101	(I)101	
ND3 - *trnA*	(I)5	(I)5	(I)5	(I)7	(O)2
trnA - *trnR*	(I)4	(I)4	(I)4	(I)4	(I)4
trnR - *trnN*	(O)1	(O)1	(O)1	(O)1	(O)1
trnN - *trnS*$_1$	(O)1	(O)1	(O)1	(O)1	
trnS$_1$- *trnE*	(O)1	(O)1	(O)1	(O)1	(O)1
trnE - *trnF*					(I)1
trnF - *ND5*					(O)1
ND5 - *trnH*	(O)4	(O)4	(O)3	(O)3	(I)2
trnH - *ND4*			(O)1	(O)1	(I)1
ND4 - *ND4L*	(O)10	(O)10	(O)10	(O)10	(I)11
ND4L – *ND6*	(I)33	(I)33	(I)33	(I)34	(I)39
ND6 - *trnP*	(I)47	(I)48	(I)48	(I)55	(I)219
trnP - *trnT*					(I)1
trnT - *CytB*	(I)7	(I)7	(I)7	(I)10	(I)4
CytB - *trnS*$_2$	(O)10	(O)10	(O)10	(O)12	(O)1
trnS$_2$ - *ND1*	(I)20	(I)20	(I)20	(I)22	(I)17
ND1 - *trnL*$_1$	(I)1	(I)1	(I)1	(O)1	(I)1
trnL$_1$ - *lrRNA*					
lrRNA - *trnV*					
trnV - *srRNA*	(I)4	(I)4	(I)4	(I)2	
srRNA - A+T-rich region					
Total nucleotides	(O)52<br>(I)277	(O)52<br>(I)278	(O)52<br>(I)278	(O)55<br>(I)284	(O)16<br>(I)350

Table S9

Continued (9/11).

Region	*Peregrinus maidis*	*Sogatella furcifera* (Hainan, China)	*Sogatella furcifera* (Yunnan, China)	*Sogatella vibix*	*Laodelphax striatellus* (Jiangsu, China)
trnI - trnQ	(I)16	(I)2	(I)2	(I)2	(I)2
trnQ - trnM	(O)1	(O)1	(O)1	(O)1	(O)1
trnM - ND2					
ND2 - trnC	(O)2	(O)2	(O)2	(O)2	(I)3
trnC - trnW	(I)40	(I)18	(I)18	(I)16	(I)34
trnW - trnY	(I)1	(I)10	(I)10	(I)9	(I)3
trnY - COI	(I)8	(I)1	(I)1	(I)1	(I)4
COI - trnL$_2$					
trnL$_2$ - COII					
COII - trnK	(I)1			(I)1	
trnK - trnD					
trnD - ATP8		(I)2	(I)2		
ATP8- ATP6	(O)4	(O)7	(O)7	(O)7	(O)7
ATP6- COIII					
COIII - trnG					
trnG - ND3					
ND3 - trnA	(I)1			(O)1	
trnA - trnR	(I)4	(I)4	(I)4	(I)4	(I)4
trnR - trnN	(O)1	(O)1	(O)1	(O)1	(O)1
trnN - trnS$_1$	(O)1			(O)1	(O)14
trnS$_1$- trnE	(O)1			(O)1	
trnE - trnF	(O)1				(O)1
trnF - ND5					
ND5 - trnH	(I)2				
trnH - ND4	(I)1				(I)2
ND4 - ND4L	(O)7	(O)7	(O)7	(O)7	(O)7
ND4L – ND6	(I)53	(I)49	(I)49	(I)49	(I)45
ND6 - trnP	(I)338	(I)93	(I)94	(I)75	(I)58
trnP - trnT	(I)1	(I)3	(I)3	(I)2	(I)4
trnT - CytB	(I)4	(I)4	(I)4	(I)4	(I)4
CytB - trnS$_2$	(O)1			(O)2	
trnS$_2$ - ND1	(I)17	(I)17	(I)17	(I)17	(I)17
ND1 - trnL$_1$	(I)1	(I)1	(I)1	(I)1	(I)1
trnL$_1$ - lrRNA					
lrRNA - trnV					
trnV - srRNA					
srRNA - A+T-rich region					
Total nucleotides	(O)19<br>(I)488	(O)18<br>(I)204	(O)18<br>(I)205	(O)23<br>(I)181	(O)31<br>(I)181

Table S9

Continued (10/11).

Region	*Laodelphax striatella* (Suwon, Korea)	*Laodelphax striatella* (Milyang, Korea)	*Laodelphax striatellus* A1 (China)	*Laodelphax striatellus* B1 (China)
trnI - *trnQ*	(I)2	(I)2	(I)2	(I)2
trnQ - *trnM*	(O)1	(O)1	(O)1	(O)1
trnM - *ND2*				
ND2 - *trnC*	(I)3	(I)3	(I)3	(I)3
trnC - *trnW*	(I)34	(I)34	(I)34	(I)34
trnW - *trnY*	(I)3	(I)3	(I)3	(I)3
trnY - *COI*	(I)4	(I)4	(I)4	(I)4
COI - *trnL*$_2$				
trnL$_2$ - *COII*				
COII - *trnK*				
trnK - *trnD*				
trnD - *ATP8*				
ATP8- *ATP6*	(O)7	(O)7	(O)7	(O)7
ATP6- *COIII*				
COIII - *trnG*				
trnG - *ND3*				
ND3 - *trnA*		(O)1		
trnA - *trnR*	(I)4	(I)4	(I)4	(I)4
trnR - *trnN*	(O)1	(O)1	(O)1	(O)1
trnN - *trnS*$_1$	(O)14	(O)14	(O)14	(O)14
trnS$_1$- *trnE*				
trnE - *trnF*	(O)1	(O)1	(O)1	(O)1
trnF - *ND5*				
ND5 - *trnH*				
trnH - *ND4*	(I)2	(I)2	(I)2	(I)2
ND4 - *ND4L*	(O)7	(O)7	(O)7	(O)7
ND4L – *ND6*	(I)45	(I)45	(I)45	(I)45
ND6 - *trnP*	(I)68	(I)68	(I)58	(I)55
trnP - *trnT*	(I)4	(I)4	(I)4	(I)4
trnT - *CytB*	(I)4	(I)4	(I)4	(I)4
CytB - *trnS*$_2$	(O)2	(O)2		
trnS$_2$ - *ND1*	(I)17	(I)17	(I)17	(I)17
ND1 - *trnL*$_1$	(I)1	(I)1	(I)1	(I)1
trnL$_1$ - *lrRNA*				
lrRNA - *trnV*				
trnV - *srRNA*				
srRNA - A+T-rich region				
Total nucleotides	(O)33<br>(I)191	(O)34<br>(I)191	(O)31<br>(I)181	(O)31<br>(I)178

Table S9

Continued (11/11).

Region	*Laodelphax striatellus* (Beijing, China)
trnI - trnQ	(I)2
trnQ - trnM	(O)1
trnM - ND2	
ND2 - trnC	(I)3
trnC - trnW	(I)34
trnW - trnY	(I)3
trnY – COI	(I)4
COI - trnL$_2$	
trnL$_2$ - COII	
COII - trnK	(O)20
trnK - trnD	
trnD - ATP8	
ATP8- ATP6	(O)7
ATP6- COIII	
COIII - trnG	
trnG - ND3	
ND3 - trnA	(O)1
trnA - trnR	(I)4
trnR - trnN	(O)1
trnN - trnS$_1$	(O)4
trnS$_1$- trnE	(O)6
trnE - trnF	(O)1
trnF - ND5	
ND5 - ND4	(O)14
ND4 - ND4L	(O)7
ND4L – trnH	(I)72
trnH – ND6	(O)1
ND6 - trnP	
trnP - trnT	(I)4
trnT - CytB	(I)4
CytB - trnS$_2$	(O)15
trnS$_2$ - ND1	(O)20
ND1 - trnL$_1$	(I)1
trnL$_1$ - lrRNA	
lrRNA - trnV	
trnV - srRNA	
srRNA - A+T-rich region	
Total nucleotides	(O)98<br>(I)131

(O), overlapping sequences; (I), intergenic spacer sequences. An empty column indicates that neighboring genes were abutting to each other.

‡, partially or fully re-annotated in this study.
